# Crystal Modifications of a Cyclic Guanosine Phosphorothioate Analogue, a Drug Candidate for Retinal Neurodegenerations

**DOI:** 10.1002/open.202300141

**Published:** 2023-10-25

**Authors:** Oswaldo Pérez, Nicolaas Schipper, Valentina Leandri, Per H. Svensson, Martin Bohlin, Thorsteinn Loftsson, Martin Bollmark

**Affiliations:** ^1^ Chemical Processes and Pharmaceutical Development Research Institutes of Sweden Forskargatan 20 J 151 36 Södertälje Sweden; ^2^ Faculty of Pharmaceutical Sciences University of Iceland Sæmundargata 2 102 Reykjavík Iceland; ^3^ Department of Chemistry Applied Physical Chemistry KTH Royal Institute of Technology 100 44 Stockholm Sweden

**Keywords:** Configuration determination, Drug design, Neurological agents, Nucleotides, Solid-state structures

## Abstract

In contribution to the pharmaceutical development of cyclic guanosine monophosphorothioate analogue **cGMPSA** as a potential active pharmaceutical ingredient (API) for the treatment of inherited retinal degenerations (IRDs), its neutral form (**cGMPSA‐H**) and salts of sodium (**‐Na**), calcium (**‐Ca**), ammonium (**‐NH_4_**), triethylammonium (**‐TEA**), tris(hydroxymethyl)aminomethane (**‐Tris**), benethamine (**‐Bnet**), and benzathine (**‐BZ**) were prepared. Their solid‐state properties were studied with differential scanning calorimetry, thermogravimetric analysis, hot‐stage microscopy, and dynamic vapor sorption, and their solubilities were measured in deionized H_2_O as well as aqueous HCl and NaOH buffers. A total of 21 crystal modifications of **cGMPSA** were found and characterized by X‐ray powder diffraction. Despite their crystalline character, no API forms featured any observable melting points during thermal analyses and instead underwent exothermic decomposition at ≥163 °C. Both the vapor sorption behavior and solubility were found to differ significantly across the API forms. **cGMPSA‐BZ** featured the lowest aqueous solubility and hygroscopicity, with 50 μg/mL and 5 % mass gain at maximum relative humidity. The synthesis and crystallization of some crystal modifications were upscaled to >10 g. Single crystal X‐ray diffraction was performed which resulted in the first crystal structure determination and absolute configuration of a cyclic guanosine monophosphorothioate, confirming the *R*_P_‐ conformation at the phosphorus atom.

## Graphical abstract

Cyclic guanosine phosphorothioate (cGMPS) analogues are emerging drug candidates for untreatable neurodegenerations of the retina, which calls for a unique case of drug development. We explore, prepare, and characterize crystalline API forms in search for a sparingly soluble crystal modification suitable for sustained‐release formulations. For the first time, the determination of the absolute configuration for a cyclic guanosine thiophosphate analogue is reported.

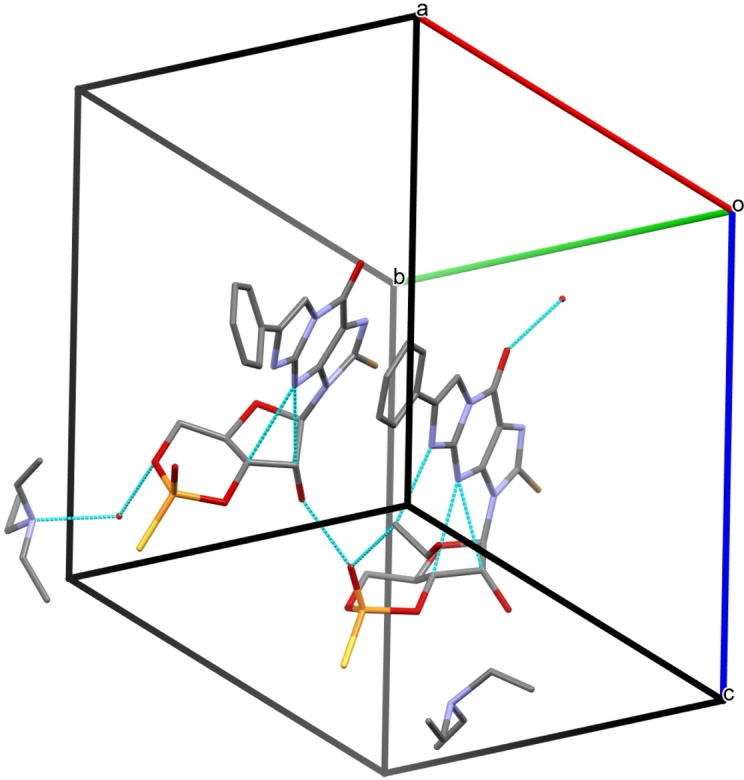

## Introduction

**Figure 1:**
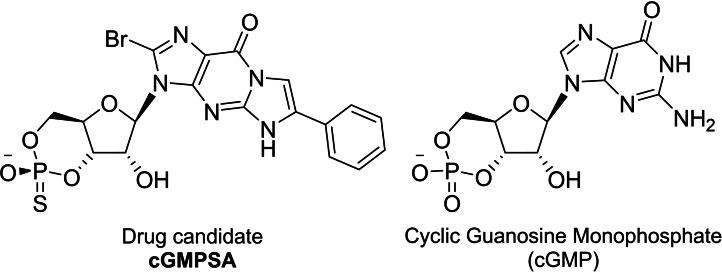
Drug candidate **cGMPSA** (left), an *R*_P_‐cyclic phosphorothioate analogue of natural cyclic guanosine monophosphate (cGMP, right).

Inherited retinal degenerations (IRDs) remain a devastating class of progressive diseases often resulting in blindness, with currently no treatment available on the market. Recent years have seen the emergence of a potential IRD drug treatment in *R*_P_‐8‐bromo‐β‐phenyl‐1,*N* ^2^‐ethenoguanosine‐3′,5′‐cyclic guanosine monophosphorothioate (**cGMPSA**),^[1]^ an analogue of natural cyclic guanosine monophosphate (cGMP). Both compounds are shown in Figure 1. Our team then undertook the first early chemical process development, scale‐up, and preparation of **cGMPSA**.^[2]^ With access to larger amounts of the active pharmaceutical ingredient (API), focus shifted to contributing to its development as a drug compound for IRDs.

Development of drug formulations targeted to the retina presents unique challenges. For instance, the most common routes of administration are intravitreal or subretinal injections,^[3]^ where reducing the frequency of injections needed for effective treatment is vital. This is to minimize patient discomfort, risk of complications, and burden on the trained medical staff required to perform the procedure. To achieve this, the drug's duration of action must be maximized, and our approach for accomplishing this was to pursue API forms with low aqueous solubilities.

Solid‐state properties of APIs including solubility can be modulated by selecting appropriate counter‐ions for use as salts of the parent compound.^[4]^ However, pharmaceutically accepted counter‐ions have mostly been developed and approved with solubility‐enhancing goals in mind.^[5]^ Furthermore, most APIs have historically been weak bases, whereas **cGMPSA** is strongly acidic.^[5b]^ The list of potential **cGMPSA** counter‐ions which are both pharmaceutically acceptable, basic, and sparingly soluble is therefore restricted to a handful.^[6]^

Developing stable crystalline forms of APIs represents a readily accessible means for lowering solubilities without altering the pharmacologically active structure of the drug molecule. In addition to ensuring robustness and purity, which is essential in the manufacture of drug products, it may also help increase physicochemical stability of the drug product, for instance against temperature and humidity changes.^[7]^ High hygroscopicity and phase transitions may affect the chemical and physical integrity of drug substances and their formulations,^[8]^ and should therefore be carefully followed during drug development. Building a library of **cGMPSA** crystal modifications and characterizing their solid‐state properties were therefore core endeavors in this project. Furthermore, scalable crystallization processes for manufacture of useful amounts of some promising API forms were developed when possible.

In this paper, we describe the preparation and characterization of a number of **cGMPSA** crystal modifications. Solubility studies for each API salt are presented, and analysis of their physicochemical properties by differential scanning calorimetry (DSC), thermogravimetric analysis (TGA), and dynamic vapor sorption (DVS) is shown. The crystal modifications forms for each **cGMPSA** salt form are characterized using X‐ray powder diffraction (XRPD) and catalogued with Roman numerals in order of discovery. The likely structures of some crystal modifications as evidenced by NMR, TGA, and DVS are discussed. Finally, the determined crystal structure and absolute configuration of **cGMPSA** by SXRD is also reported–the second for a cyclic nucleotide phosphorothioate analogue^[9]^ and, to the best of our knowledge, the first for a cGMP derivative.

## Results and Discussion

The **cGMPSA** forms ultimately prepared and studied were the free acid (**cGMPSA‐H**) and salts of sodium (**cGMPSA‐Na**), calcium (**cGMPSA‐Ca**), ammonium (**cGMPSA‐NH_4_**), triethylamine (**cGMPSA‐TEA**), tris (**cGMPSA‐Tris**), benethamine (**cGMPSA‐Bnet**), and benzathine (**cGMPSA‐BZ**). Their structures and corresponding synthetic protocols are found in the Supporting Information. Of the selected bases, only calcium, benethamine, and benzathine fulfil the criteria of precedent as sparingly‐soluble pharmaceutical salts, with the two organic amines historically only used as counterions for penicillin derivatives in sustained‐release formulations.[^6^, ^10^] Sodium, ammonium, and tris(hydroxymethyl)aminomethane are commonly used pharmaceutical counter‐ions, but often result in salts with high aqueous solubility. Triethylammonium was also studied: despite not being currently listed as a GRAS substance and having no precedent of use as a pharmaceutical counter ion, the current synthetic route naturally yields **cGMPSA** as a salt of triethylammonium. This makes it an ideal salt form from an availability perspective, whereas additional steps are required to obtain other API forms.^[2]^

### Solid‐State Characterization

#### Solubility Studies

Considering its multiple hydrogen‐bonding sites, strongly acidic phosphorothioate, and a predicted Log*P* value of approximately −0.4 (MarvinSketch Software), **cGMPSA** can be expected to be hydrophilic. However, the measured solubilities in deionized H_2_O were found to be low (see Table 1). The free acid showed a solubility of 1.7 mg/mL, whereas the solubility of the different salts varied considerably; a three order of magnitude difference is observed between the least and most soluble salts, that is, 0.01 versus 38 mg/mL for the benzathine and sodium salts, respectively. Based on their comparatively low solubility, the API salts of benzathine and benethamine may be promising candidates for sustained‐release drug formulations intended to treat IRDs.

**Table 1:** Solubilities of API forms in deionized H_2_O. All measurements made in quadruplicates.

Compound	Solubility [mg/mL]	pH at saturation solubility
**cGMPSA‐H**	1.7 (±0.1)	2.5 (±0.2)
**cGMPSA‐TEA**	8.8 (±1.7)	4.4 (±0.4)
**cGMPSA‐Na**	38.4 (±11.1)	5.5 (±0.2)
**cGMPSA‐Ca**	0.28 (±0.2)	6.9 (±0.5)
**cGMPSA‐NH_4_**	7.9 (±8.0)	3.2 (±0.1)
**cGMPSA‐Tris**	13.5 (±3.5)	8.5 (±0.1)
**cGMPSA‐Bnet**	0.05 (±0.03)	7.2 (±0.04)
**cGMPSA‐BZ**	0.01 (±0.01)	6.8 (±0.1)

**Figure 2:**
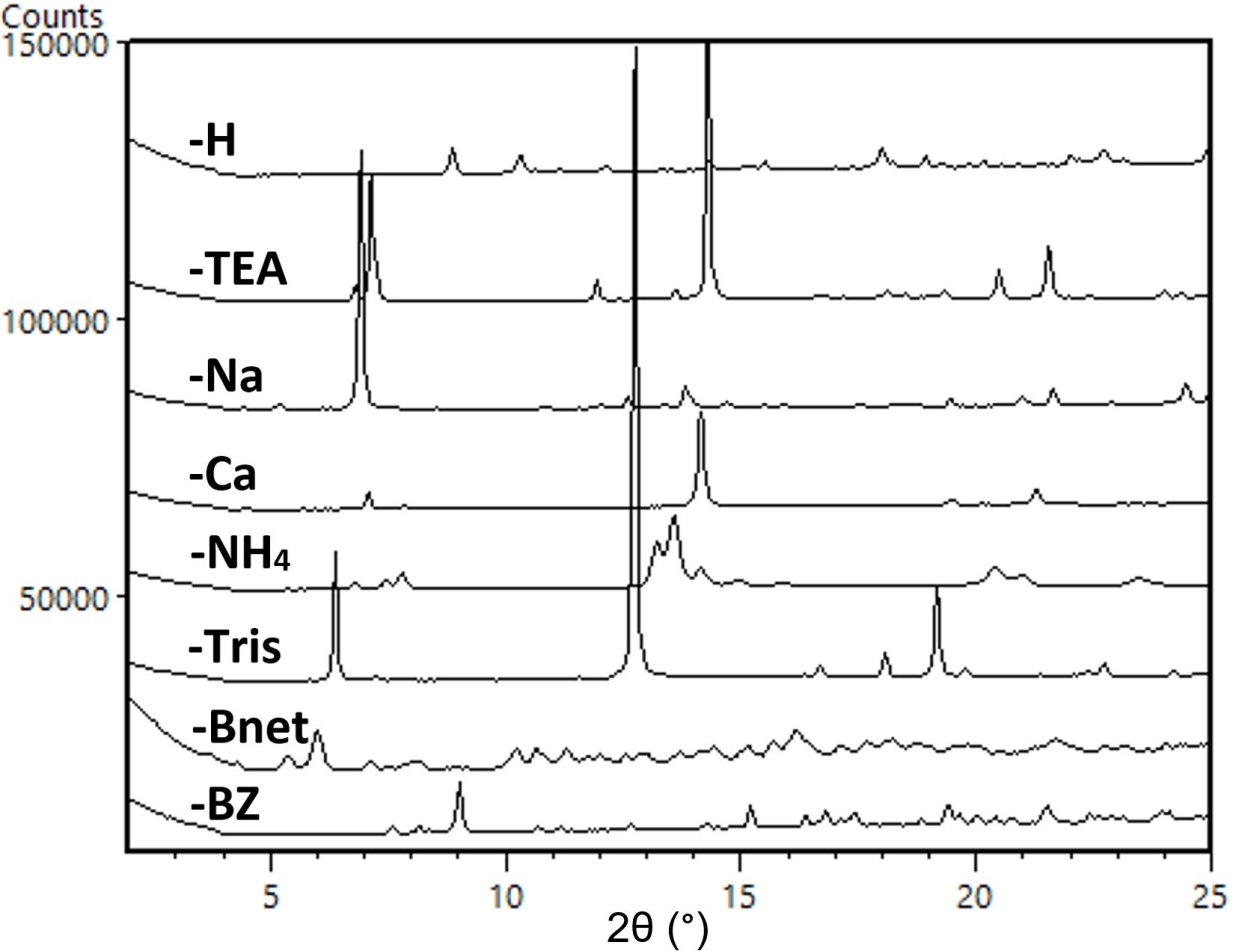
Expanded diffractograms of each API salt crystal modification obtained from water, corresponding to the aqueous solubilities in Table 1. Full diffractograms available in the SI.

XRPD analysis of the solids from solubility studies showed that all **cGMPSA** salts underwent a phase‐change from their initial crystal modification. Meanwhile, the starting crystal modification for **cGMPSA‐H** did not change. The measured solubilities in Table 1 correspond to the crystal modification shown in Figure 2 below.

The solubility of this API is expected to increase with increasing pH due to the highly acidic phosphorothiotic acid moiety (p*K*_a_=1.68 predicted by MarvinSketch software) in addition to multiple ionizable groups on the ribose and nucleobase. Measurements at low pH values should therefore reflect the solubilities of the parent acid, **cGMPSA‐H**, whereas measurements at high pH should reflect the solubility of the ionized form and salt. To study this, a solubility study was performed in 0.1 M aqueous HCl and NaOH buffers, and the corresponding solids were analyzed by XRPD. The pH‐dependent solubility data can be found in the Supporting Information (Supporting Information, p. 60).

A positive correlation between solubility and pH was found, with large differences in solubility across the pH range. Solubilities in 0.1 M HCl varied between 0.1 and 25 μg/mL for the various salts tested. For some salts (**cGMPSA‐Na**, **‐Ca**, **‐NH_4_**, **‐BZ**), XRPD analysis returned diffractograms matching that of **cGMPSA‐H**, confirming that the measured solubilities correspond to the free API form. Similarly, after treatment of mixtures of **cGMPSA‐TEA** in 0.1 M NaOH with 0.1 M HCl until neutral pH ranges, the solids were identified as **cGMPSA‐Na** by XRPD. It is worth noting the significant variance in the measured solubility data for some of the salts/systems despite equilibration for several days and the experiments being performed in quadruplicates. This could be the result of small variations in pH, particularly when close to p*K*_a_ values of functional groups in the compounds. In turn, raising the pH and repeated pH adjustments often caused samples to form thick gels which was prohibitive for analysis or further treatments, and more detailed pH‐solubility profile measurements were therefore not pursued.

#### Thermal and sorption studies

**Figure 3:**
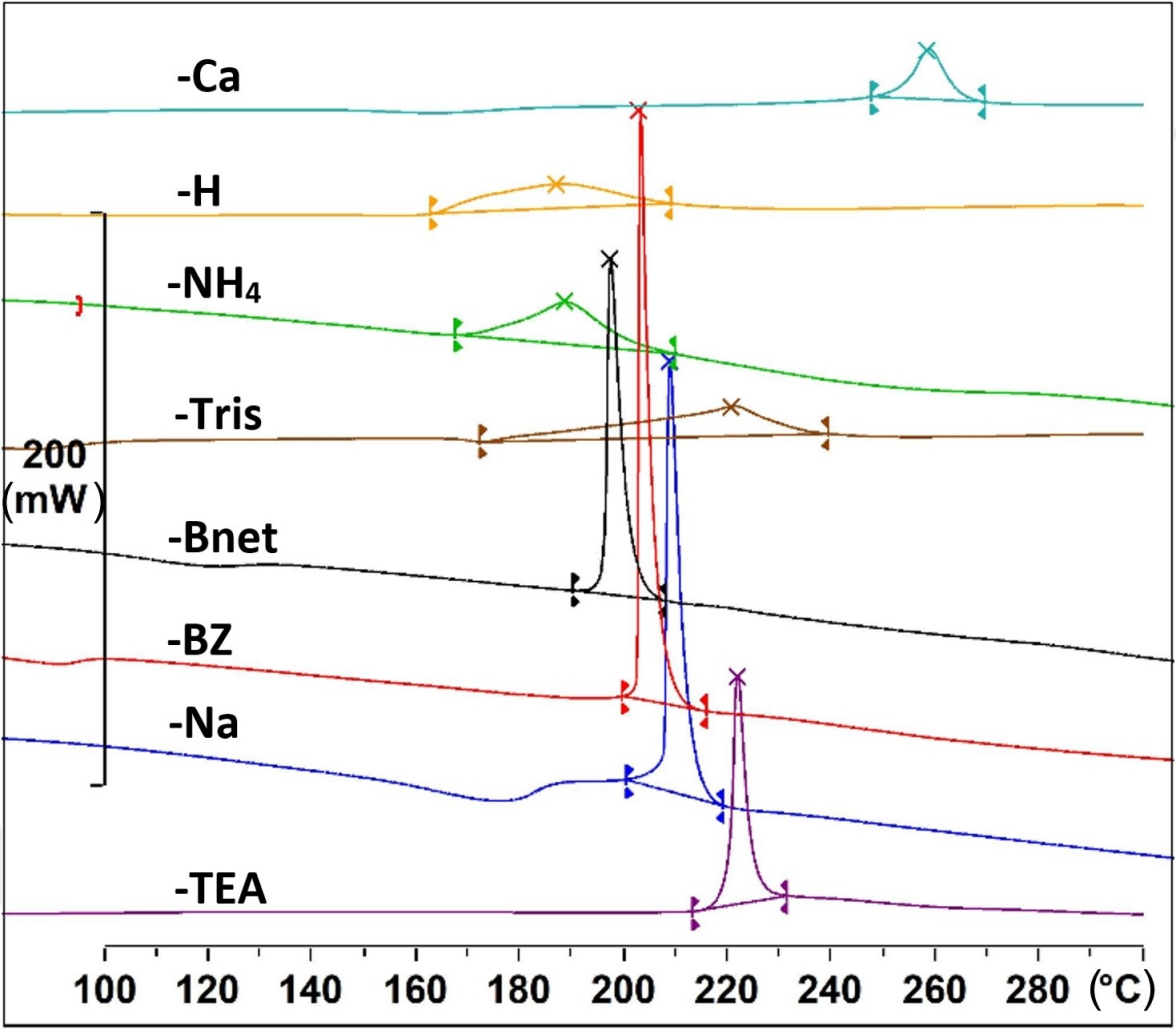
Expanded DSC curves of **cGMPSA‐H** and its salt forms, with normalized Y axis. See the Supporting Information for more detailed DSC/TGA curves.

All **cGMPSA** forms displayed similar thermal behavior on DSC and TGA, that is, no melting points could be observed despite them being crystalline, and the analyte eventually underwent decomposition. Decomposition shows as an exotherm on DSC in the 164–253 °C range, depending on the salt studied, and is associated with simultaneous onset of significant mass loss on TGA. No other thermal events were evident except for early mass loss from desolvation in some cases. **cGMPSA‐H** featured a broad decomposition exotherm with a significantly earlier onset compared to its salt forms (163.6 °C). **cGMPSA‐Ca** exhibits the highest onset as well as lowest enthalpy of decomposition (253.2 °C and 162.3 J/g, respectively). The DSC curves for all API forms are compiled in Figure 3 below (Full‐size TGA and DSC graphs can be found in the Supporting Information).

**Figure 4:**
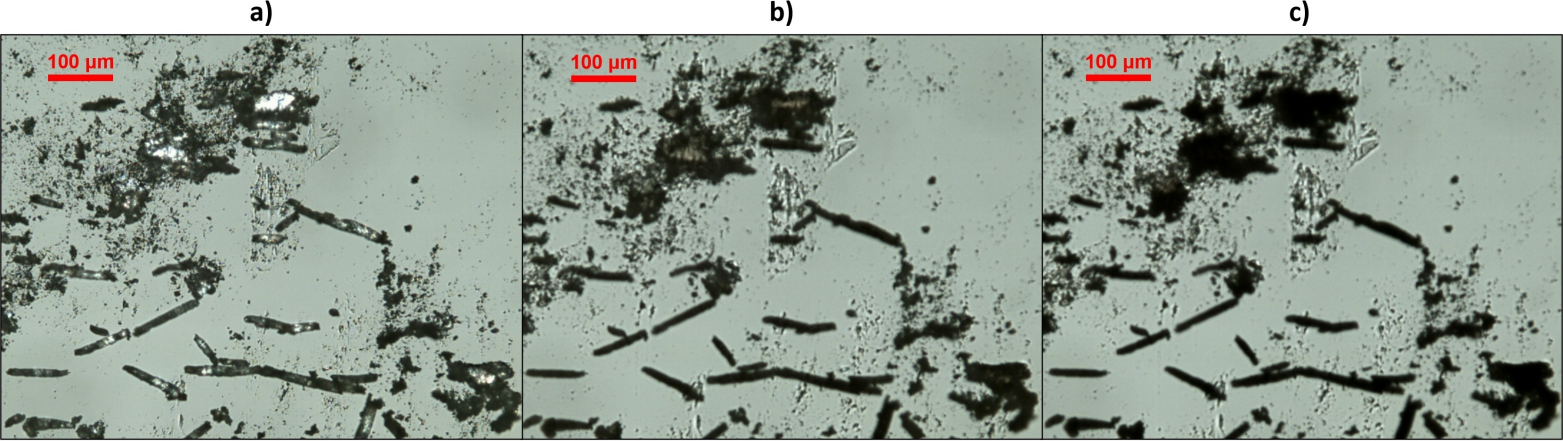
Hot‐stage microscopy of **cGMPSA‐Na**. a) Room temperature. b) 224 °C. c) 245 °C. Decomposition onset for this material according to DSC is 212 °C. Loss of crystallinity is evident by the loss of light polarization and darkening of particles.

To confirm the lack of melting points observed on DSC, a follow‐up hot‐stage microscopy experiment was performed on **cGMPSA‐Na**. In agreement with DSC/TGA results, no melting or morphological changes were seen, and a slow and gradual darkening occurred until a black char remained (Figure 4).

Higher melting points are often correlated with higher physical stability and thus lower solubility.^[11]^ However, the same is not true for decomposition temperatures. **cGMPSA‐Bnet** displayed significantly lower solubility than its **‐TEA** and **‐Na** counterparts but featured lower thermal stability. Thus, no predictions or comparisons can be made about the solubility or physical stability of these API salt forms based on their thermal characteristics.

DVS showed varying levels of hygroscopicity as well as potential crystal hydrate formation for the different **cGMPSA** salts. Other techniques often used complementary to DVS such as Karl Fischer titration and NMR spectroscopy could not give accurate determinations of water content. The former could not be used due to the reactivity of the phosphorothioate with iodine used in the titration, and NMR spectroscopy gave broad and shifting (in terms of chemical shift) signals from exchangeable protons, compounded by DMSO, itself hygroscopic, being the only viable NMR solvent.

The least hygroscopic salts were the **‐TEA** and **‐BZ** salts with about 5 % change in mass (Δ_m_) at 95 % relative humidity (RH). The most hygroscopic salts were **cGMPSA‐Tris** and **‐Ca** with a significantly higher Δ_m_ of 32 % and 22 %, respectively. Table 2 compares the hygroscopicity of various salts in at 30 %, 60 %, 95 % RH during sorption and desorption, as well as after the final desorption cycle.

**Table 2:** Water sorption of different **cGMPSA** salts measured as Δ_m_ at different RH values during sorption cycle 1 (S1) and desorption cycle 2 (D2).^[a]^

RH	30 %		60 %		95 %		0 %
Sorption cycle	S1	D2	S1	D2	S1	D2	D2
**cGMPSA‐H**	5.3	5.7	6.2	6.7	9.1	9.3	0.3
**cGMPSA‐TEA**	3.4	3.5	4.0	4.8	5.0	5.2	−1.0
**cGMPSA‐Na**	2.2	6.3	8.1	12.5	15.1	15.1	−0.3
**cGMPSA‐Ca**	5.3	12.4	7.1	15.5	21.9	22.0	1.9
**cGMPSA‐Tris**	4.9	5.5	6.0	6.8	32.8	32.4	3.3
**cGMPSA‐Bnet**	3.4	4.4	5.2	6.1	8.0	7.7	0.7
**cGMPSA‐BZ**	0.6	0.7	1.9	3.9	4.8	4.7	−0.2

[a] DVS was not performed for **cGMPSA‐NH_4_**.

**Figure 5:**
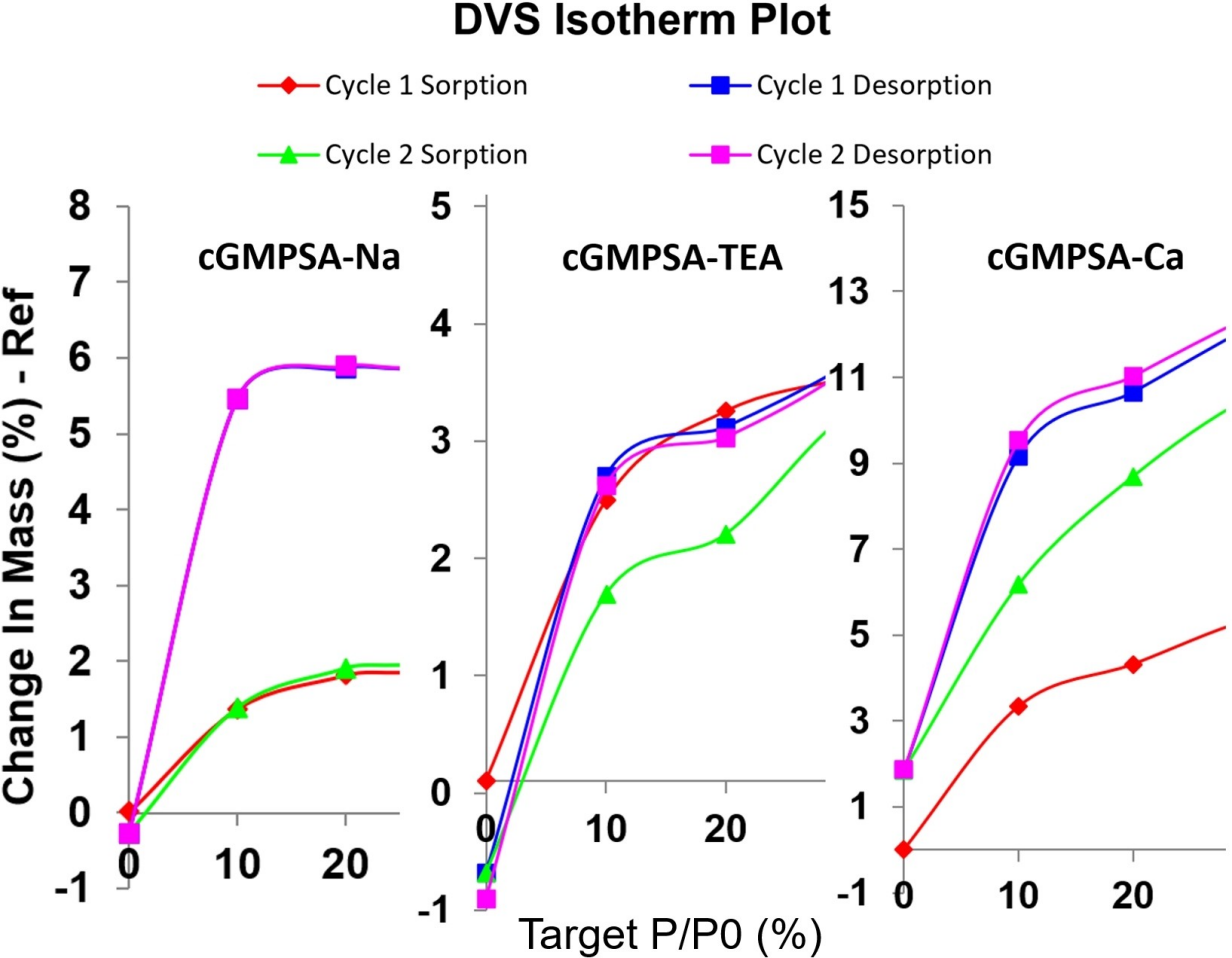
Expanded DVS isotherm plots for **cGMPSA** salts of sodium (left), triethylammonium (center), and calcium (right), highlighting differences in reversibility of sorption.

The uptake of water by some salts was not completely reversible for the **cGMPSA‐TEA**, ‐Ca, and ‐Tris salts, indicated by the Δ_m_ deviating from 0 at 0 % RH upon desorption (Table 2, Figure 5), as well as a narrowed hysteresis on the second sorption‐desorption cycle compared to the first. The negative Δ_m_ value for **cGMPSA‐TEA** after desorption may indicate equilibration as an ansolvate form after loss of solvents during the first cycle that was already present prior to analysis. In contrast, the starting **cGMPSA‐Na** shows a reversible phase transition between 0–10 % RH, with a return to ~0 Δ_m_ after desorption.

On close inspection of DVS isotherms, changes in mass were not continuous with changes in RH for most salts. Instead, sorption occurred in a stepwise fashion. This may be explained by formation of crystalline hydrates, with sharp inflection points in the isotherms owing to rapid sorption of water into the bulk triggering the phase transition. The compound then sorbs moisture on the surface, which manifests as a plateau in the isotherm from decreased Δ_m_.^[12]^ **cGMPSA‐TEA**, **‐Na**, and **‐BZ** clearly exhibited such step‐wise changes in mass on DVS (Supporting Information, p. 13, 20, and 57, respectively). However, formation of crystal solvates or hydrates on DVS should be confirmed by techniques such as XRPD. Table 3 below highlights the apparent relationship between the observed DVS data and the corresponding theoretical hydrate masses.

**Table 3:** Water sorption of **cGMPSA** salts at different %RH compared to theoretical mass changes of hydrate formation.

Compound	Theoretical Δm/H_2_O [%]	Observed Δm [%]	Closest Corresponding Hydrate	Stable %RH range
**cGMPSA‐H**	3.3	None	–	–
**cGMPSA‐TEA**	2.8	2.6^[a]^	⋅1H_2_O	10–20
		3.6^[a]^	⋅1.5H_2_O	10–70
		5.4^[a]^	⋅2H_2_O	40–95
**cGMPSA‐Na**	3.2	1.4	⋅0.5H_2_O	10–30
		5.5	⋅1.5H_2_O	10–60
		11.6	⋅4H_2_O	40–95
**cGMPSA‐Ca**	1.6	1.9	⋅1H_2_O	0
**cGMPSA‐Tris**	2.7	3.3	⋅1H_2_O	0–70
**cGMPSA‐Bnet**	2.4	None	–	–
**cGMPSA‐BZ**	1.3	1.3	⋅H_2_O	40–70
		3.9	⋅3H_2_O	60–95

[a] Corrected for equilibration at Δ_m_=−1 % after cycle 1 desorption.

**Figure 6:**
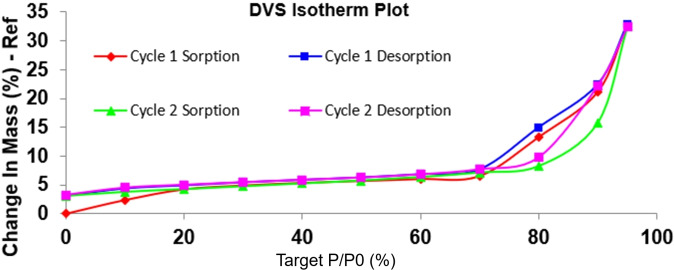
Isotherm plot showing vapor sorption profile of **cGMPSA‐Tris**.

The **cGMPSA‐Bnet** and **cGMPSA‐H** isotherms feature a continuous gradient in the Δ_m_, with no evidence of hydrate formation. Meanwhile, **cGMPSA‐Tris** featured a unique vapor sorption behavior (Figure 6). There is an initial early sorption of approximately Δ_m_=3 % which is consistent with sorption of 1 equivalent of H_2_O (Table 3), and in agreement with a 3.7 % mass loss observed on TGA (Supporting Information, p. 44). The compound then showed very little water sorption between 0–70 % RH. Above 70 % RH, the material experiences a dramatic but reversible Δ_m_ resembling deliquescence, and the compound equilibrated at Δ_m_=3.3 after both desorption cycles.

### Synthesis of cGMPSA Crystal Modifications

**Figure 7:**
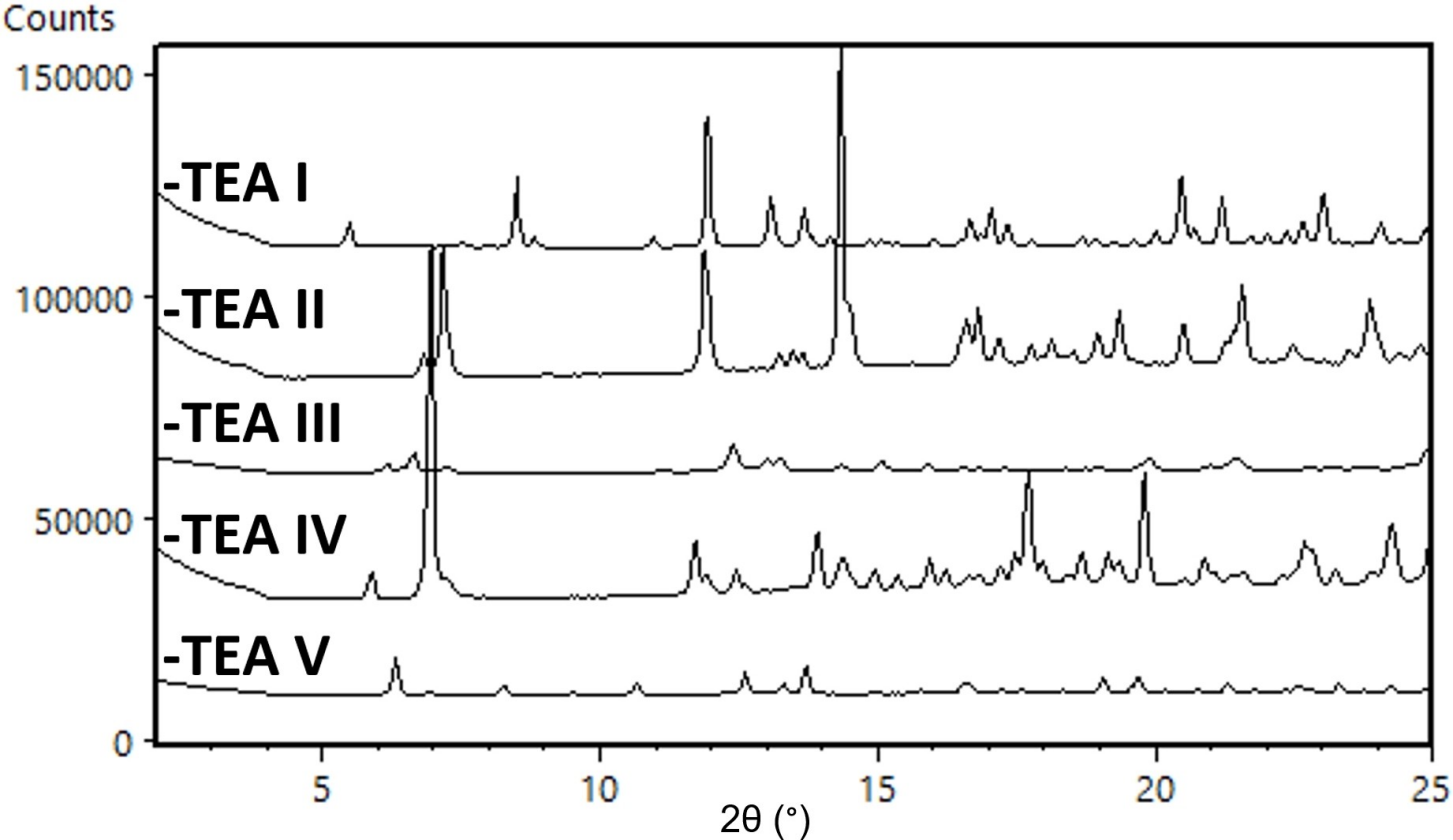
Expanded diffractograms of **cGMPSA‐TEA** crystal modifications **I**–**V**.

**cGMPSA‐TEA**. A total of five crystal modifications of the **cGMPSA‐TEA** salt have been found so far as indicated by different XRPD diffractograms and NMR. Their diffractograms are compiled in Figure 7. The first crystal modification was obtained from the synthetic process detailed in our previous report,^[2]^ and was characterized as a cocrystal with one excess equivalent of triethylammonium fluoride. This was based on a signal at −160 ppm by ^19^F NMR spectroscopy, a 2 : 1 base:API ratio from the ^1^H NMR spectrum, and its unique diffraction pattern in XRPD. HPLC showed a purity of only 96 %, and DSC/TGA analyses showed significant thermal instability (Data in Supporting Information, p. 5–12).

To increase purity, thermal stability, and to remove excess triethylammonium from the product, further recrystallization was desired, and the compound was subjected to some crystallization development work. First, a polymorph screen was undertaken as described in the experimental section, and the approximate solubility as well as phase changes to new crystal modifications were recorded. The results are summarized in Table 4.

**Table 4:** Approximate solubility of **cGMPSA‐TEA** in some solvent systems and the crystal modifications obtained thereof.

Solvent	Solubility [μg/mL]	Crystal Modification
H_2_O	Not available (NA)^[a]^	NA
MeCN	114	**cGMPSA‐TEA II**
MeCN+H_2_O^[b]^	>40000^[c]^	NA
MeOH	40	**cGMPSA‐TEA III**
MeOH+H_2_O^[b]^	>40000^[c]^	NA
EtOH	1610	**cGMPSA‐TEA IV**
EtOH+H_2_O^[b]^	>40000^[c]^	NA
EtOH+THF^[b]^	>40000^[c]^	**cGMPSA‐TEA IV**
2‐PrOH	105	Amorphous
*n*‐BuOH	210	**cGMPSA‐TEA II**
Acetone	285	**cGMPSA‐TEA II**
THF	28	**cGMPSA‐TEA II**
THF+H_2_O^[b]^	>40000^[c]^	NA
MeTHF	10	No change (NC)
Dimethoxyethane	15	NC
Dioxane	30	NC
Diethyl ether	3	NC
EtOAc	5	NC
*i*‐PrOAc	10	NC
Dichloromethane	30	NC
DMF	>40000^[c]^	NA
DMSO	>40000^[c]^	NA

[a] Gel formation. [b] 1 : 1 v/v ratio. [c] Solution.

In general, the compound was readily soluble in DMF and DMSO, formed gels or cloudy solutions in water, and was sparingly soluble in other solvents. However, it readily dissolved when equal parts of water were added to screens containing polar solvents, and vice versa. Additionally, in small amounts, water was found to encourage crystallization of **cGMPSA‐TEA I** from the reaction mixture of the final synthetic step – crystallization was found to stall when the reactor vessel was thoroughly dried and dry solvents were used, but it resumed upon addition of stoichiometric amounts of water.

No excess of triethylammonium fluoride was seen in the ^1^H and ^19^F NMR spectra of crystal modifications **II**–**IV**. HPLC purity of **cGMPSA‐TEA II** remained similar to the starting **cGMPSA‐TEA I**, whereas **cGMPSA‐TEA III** and **IV** were both >99 % pure. No solvents were observed in the NMR spectra of crystal modification **II**, but stoichiometric amounts of the corresponding alcohol were observed in that of **III** and **IV**, despite aggressive vacuum‐drying procedures at up to 60 °C overnight. This would suggest these are crystalline solvates. Furthermore, screens containing THF and EtOH, respectively, yielded crystal modifications **II** and **IV**, whereas a screen containing both solvents gave only modification **IV**, implying EtOH solvation may drive the crystallization process.

Alcohols were then selected for further recrystallization experiments. Upon upscaling and repeating recrystallizations in EtOH, a new crystal modification (**cGMPSA‐TEA V**) was obtained. DSC showed improved thermal stability for this product compared to **cGMPSA‐TEA I** (Supporting Information, p. 12). NMR analysis of crystal modification **V** showed a half‐equivalent of ethanol and a broad water peak approximately corresponding to equimolar amounts, despite vacuum‐drying at 60 °C overnight and a lack of desolvation on TGA prior to decomposition (SI, p. 12), which points to **cGMPSA‐TEA V** as a hemiethanolate monohydrate crystal modification.

**cGMPSA‐H**. As discussed earlier, the solubility for the free acid **cGMPSA‐H** was lower than those of most salt forms which makes it a potentially interesting API form for formulation. It was also considered valuable as a starting material for the synthesis of further salts due to the strong acidity of the phosphorothioate moiety, which should make salt formation with weak bases facile. Efforts to prepare the free form appeared highly sensitive to pH, with the use of TFA leading to incomplete conversion (monitored by the integral of triethylammonium signals from the starting material by ^1^H NMR spectroscopy). Stronger acids such as naphthalene‐1,5‐disulfonic acid or excess HCl resulted in inconsistencies in precipitation of the product, presumably due to over‐protonation of the compound, and it tended to deteriorate with continued handling in attempts to isolate it. A balance seems to be achieved when a slight deficit of HCl in H_2_O was used to avoid over‐protonation, but further work is required to ensure reproducibility.

During this work, two crystal modifications were observed (diffractograms in the Supporting Information, p. 25). **cGMPSA‐H I** crystallized from either aqueous or methanolic mixtures and was the predominant form. A second crystal modification **cGMPSA‐H II** was observed once in EtOH.

**cGMPSA‐Na**. While **cGMPSA‐Na** had significantly higher aqueous solubility compared to the starting **‐TEA** salt, it was less soluble in warm MeOH. This allowed for its preparation via salt exchange, as it spontaneously crystallized upon dissolution of the starting material in the hot solvent together with a sodium source.

**Figure 8:**
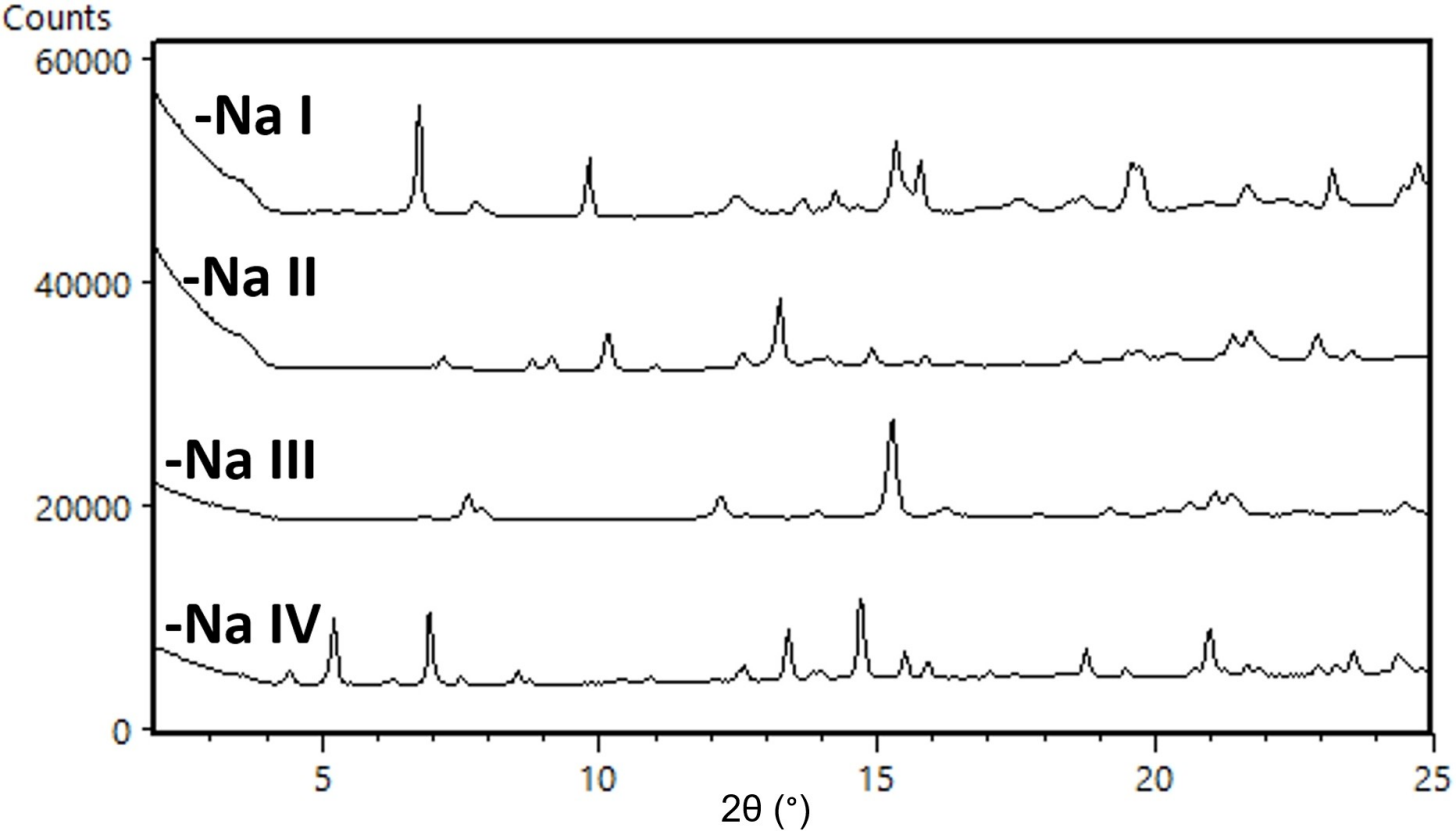
Expanded diffractograms of **cGMPSA‐Na** crystal modifications **I**–**IV**.

A total of four crystal modifications were found for this salt form and are compiled in Figure 8. Respectively, forms **I** and **II** were both obtained when air‐drying vs. vacuum‐drying precipitates from EtOH. **cGMPSA‐Na** **IV** was observed during solubility experiments in both deionized water as well as aqueous NaOH solutions at high pH. Finally, **cGMPSA‐Na III** was obtained from MeOH as mentioned above (see Supporting Information, p. 14 for experimental method). NMR showed 1.1 % w/w MeOH as the main solvent contribution after vacuum‐drying. The ~7.6 % weight loss with early onset seen on TGA (Supporting Information, p. 19) could be attributed to sorption of atmospheric moisture during handling, which is consistent with the observed vapor sorption behavior of the compound, particularly with ~6 % Δ_m_ between 10–60 % relative humidity.

**Figure 9:**
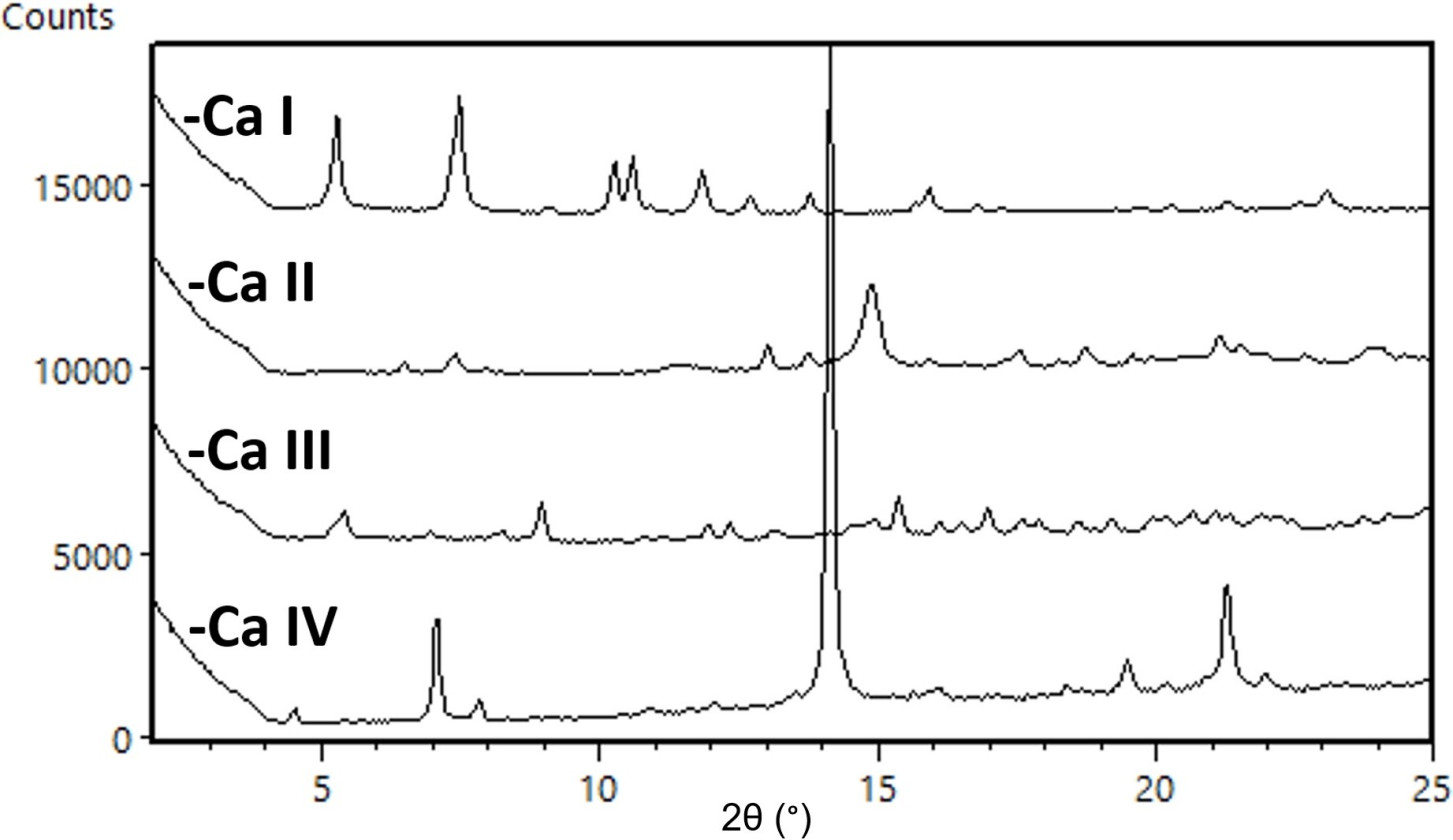
Expanded diffractograms of **cGMPSA‐Ca** crystal modifications **I**–**IV**.

**cGMPSA‐Ca**. The calcium salt of the API displayed four distinct crystal modifications during development of its preparation (Figure 9). Crystal modifications **I** and **II** were recovered when the salt was prepared using 200 mM vs. 500 mM Ca(OAc)_2_ in H_2_O, respectively. Neither the starting **cGMPSA‐TEA** nor the product were soluble in these mixtures, and slow conversions to the calcium salt were attributed to these heterogeneous reaction conditions. A homogeneous starting mixture was achieved by using warm MeOH and CaCl_2_ as the solvent and metal source, and the system gave a third crystal modification, **cGMPSA‐Ca III**. **cGMPSA‐Ca IV** was observed after slurrying the compound in deionized H_2_O during solubility studies.

**cGMPSA‐NH_4_ and cGMPSA‐Tris**. These salts were obtained as amorphous solids after treatment with the relevant base, followed by thorough concentration with MeOH and 2‐PrOH to remove neutralized triethylamine. They were both found to crystallize from deionized H_2_O (Diffractograms in the Supporting Information, p. 45) and no further screenings were performed.

**Figure 10:**
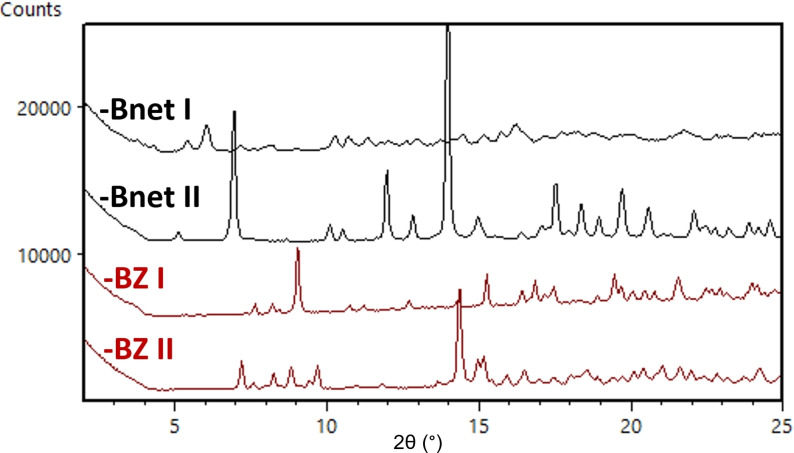
Expanded diffractograms of crystal modifications of **cGMPSA‐Bnet** (Black) and **cGMPSA‐BZ** (Red).

**cGMPSA‐Bnet and ‐BZ**. As with other salt forms, both the **‐Bnet** and **‐BZ** salts were observed to crystallize during solubility studies from deionized H_2_O, and no further solvent/polymorph screenings were performed. However, these API salts displayed the lowest overall solubilities, and there was therefore an interest in generating gram quantities of both for further pharmaceutical research. The resulting upscaling work led to discovery of a second crystal modification for each (See Figure 10).

**cGMPSA‐BZ II** was obtained by salt metathesis between **cGMPSA‐TEA** and benzathine diacetate in a 1 : 1 H_2_O/MeOH mixture. NMR spectroscopy of the dried product suggests a dihydrate polymorph, with 2 equivalents of H_2_O (2.5 % w/w) in the sample which corresponds to a 3 % weight loss on TGA.

An analogous approach was not possible for **cGMPSA‐Bnet**, as both acetate and chloride salts of benethamine were insoluble in H_2_O or MeOH. Instead, a slight excess of the free base was added to a methanolic solution of the starting **cGMPSA‐TEA**. After evaporation of the mixture, 2‐PrOH was added with the intent to remove neutral TEA by repeated co‐evaporations. However, the result was a slurry from which the desired salt was filtered and identified as **cGMPSA‐Bnet II**. NMR spectroscopy of this material suggests solvation with 1 equivalent of 2‐PrOH in agreement with the corresponding TGA desolvation mass loss of 7.3 % w/w.

### Single‐crystal X‐Ray Diffraction

The chirality of **cGMPSA** is assumed to be the *R*_P_‐configuration as evidenced by ^31^P NMR chemical shifts in agreement with configurational assignments of *R*_P_‐ and *S*_P_‐nucleotide phosphorothioates, as well as studies of binding affinity to various protein kinases.^[13]^ However, this configuration has not been confirmed for any cyclic guanosine thiophosphate analogue with single‐crystal X‐ray diffraction (SXRD) studies. To our best knowledge, *R*_P_‐uridine‐3′,5′‐cyclic phosphorothioate (cUMPS) is currently the only cyclic nucleotide thiophosphate in the literature with a solved single‐crystal structure.^[9]^

**Figure 11:**
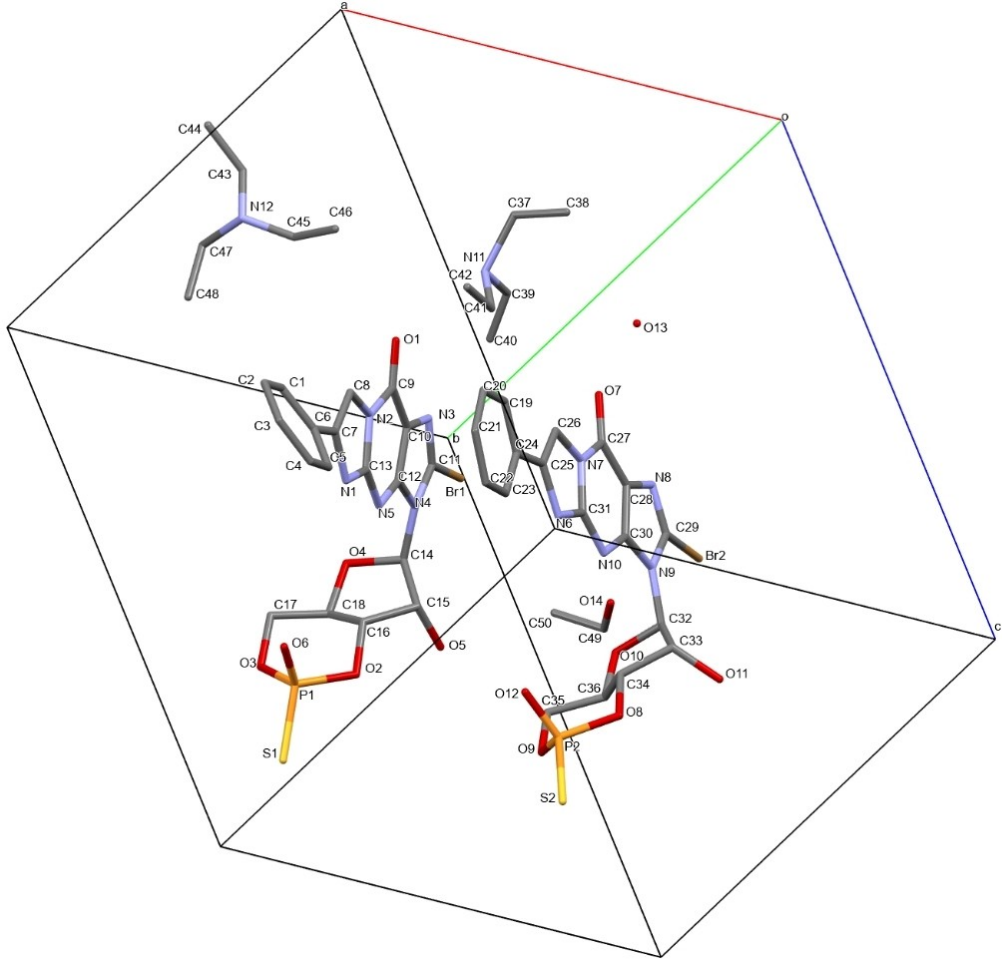
Asymmetric unit for measured crystal of **cGMPSA‐TEA** by SXRD, with labelled atoms. Hydrogen atoms have been omitted for clarity. The asymmetric unit is comprised of two symmetry‐independent **cGMPSA** molecules and counter ions. The chiral configuration at the phosphorus atoms **P1** and **P2** is *R*, determined using Flack′s X parameter.^[14]^

A crystal grown from **cGMPSA‐TEA VI**, as described in the experimental section, was analyzed by SXRD. Flack′s X chirality parameters were used to determine absolute configuration.^[14]^ In accordance with the calculated Flack parameter and the Cahn–Ingold–Prelog sequence rules,^[15]^ the stereochemistry of the phosphorus centers (P1 and P2) was determined as *R*. All other API forms feature the same chirality, as observed in the mass and NMR spectra as well as the chromatograms, where the relevant signals remain unchanged (See Supporting Information). The asymmetric unit of the solved crystal structure is shown in Figure 11 (Full‐size images and CIF file found in the Supporting Information, p. 61–64).

**Figure 12:**
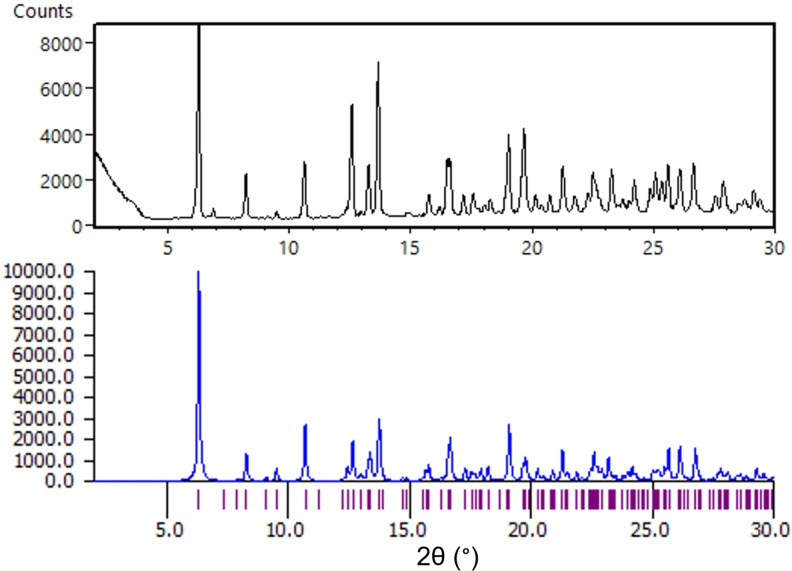
Experimental powder diffraction of **cGMPSA‐TEA IV** (top) and the simulated diffractogram from the SXRD structure using Mercury Software (bottom).

A diffraction pattern was predicted from the solved crystal structure using Mercury software, which closely matched the experimental data for **cGMPSA‐TEAH V** (Figure 12). The asymmetric unit is indeed comprised of two API molecules and a single molecule of ethanol, in agreement with the corresponding ^1^H NMR spectrum (Supporting Information, p. 10). Only one water molecule was found, compared to the two found by NMR spectroscopy. This could either be explained by the second water molecule being more loosely bound and freely evaporating without affecting the lattice, or by the hygroscopic NMR solvent absorbing moisture during sample preparation, causing overrepresentation of water in the signal.

**Figure 13:**
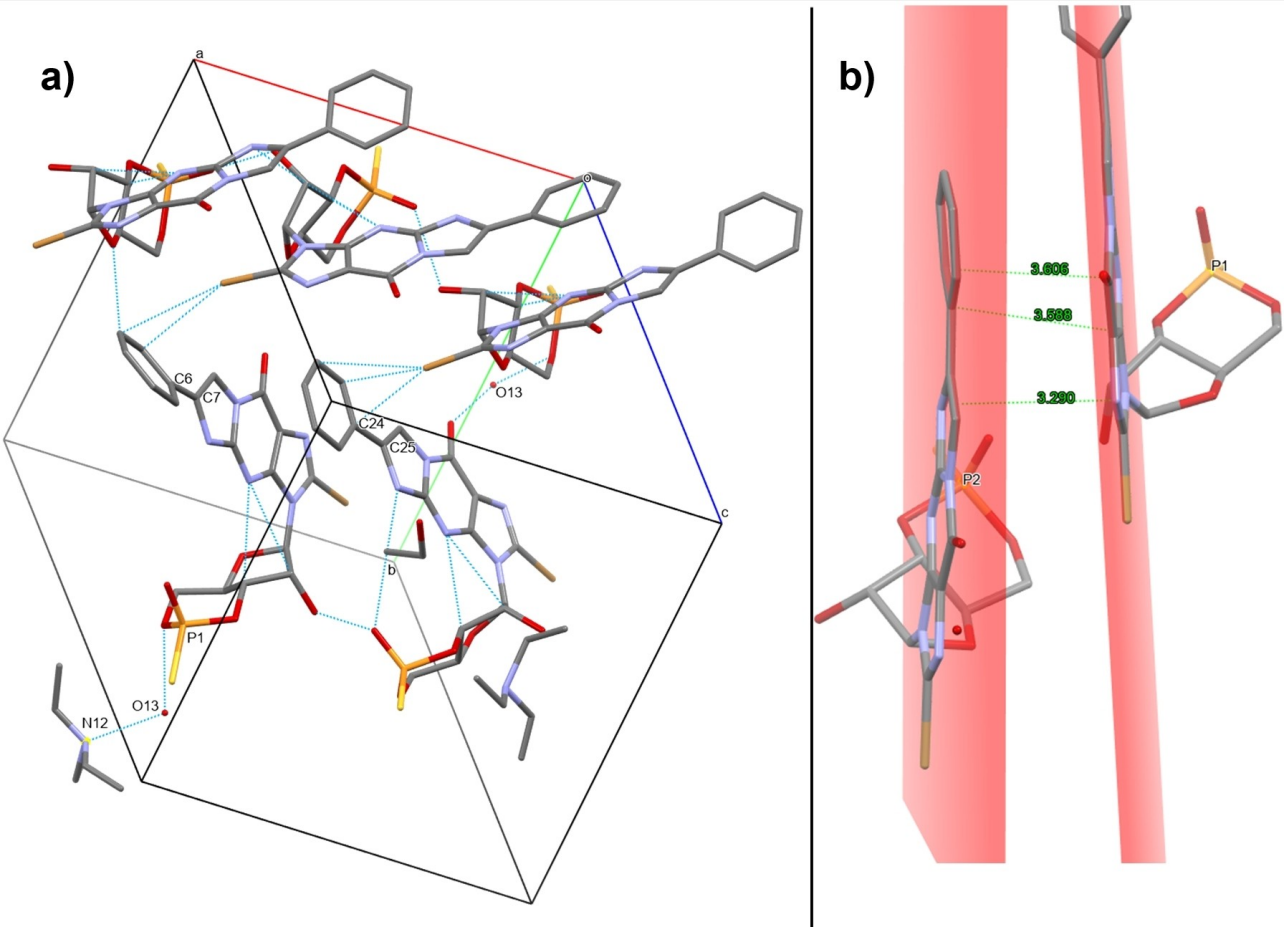
a) Unit cell view with some key molecular interactions. The ionic bonding mediated by hydrogen‐bonded water (bottom left), torsion of along the C6−C7 bonds, and packing with adjacent cell units are highlighted. b) View showing π–π interaction distances along the plane of the nucleobases within the asymmetric unit.

The ribose in both API units adopts the north conformation.^[16]^ Intermolecular H‐bonding is seen between the secondary amines, 2′‐hydroxyls, ethanol, and phosphorothioates (Figure 13). These likely stabilize the crystalline lattice, suggesting higher melting points. Indeed, the thermal analysis for **cGMPSA‐TEA V** showed no melting below 220 °C. It is also notable that the precursor to **cGMPSA**, which features a bulky silyl protecting group on the 2′‐hydroxyl H‐bonding site and therefore cannot support the observed H‐bonds, eluded all crystallization attempts during process development and could only be obtained as amorphous material.^[2]^

It had been hypothesized that the larger, planar phenylethenyl (PET) modification could facilitate crystallization due to increased π–π stacking. A distance of approximately 3.5 Å between the planes of the parallel‐displaced nucleobase pair suggests such π–π stacking is present (Figure 13).^[17]^ This interaction is not observed in cUMPS, which features a much smaller nucleobase. Yet, a departure from planarity is seen in a ~36° torsion along the C6−C7 bond of one PET group, which appears to facilitate a short contact interaction with the bromine of a neighboring molecule, related to the asymmetric unit through a 2‐fold screw axis symmetry. The interaction is satisfied in the adjacent molecules without any torsion in the corresponding C24−C25 bond.

A water molecule (O13) bridges the ionic bonding for one phosphorothioate‐triethylammonium pair (P1−N12) via hydrogen bonding. The bond lengths across the phosphorothioate, and therefore the charge distribution, closely match those observed in cUMPS (1.5 and 1.9 Å for the exocyclic P−O and P−S bonds, respectively).

## Conclusions

A crystal structure and absolute configuration for **cGMPSA‐TEA** was determined by SXRD, confirming the stereochemistry as an *R*_P_‐cyclic phosphorothioate, as well as hydrogen bonding, π–π stacking, and solvation by ethanol and water in the **cGMPSA‐TEA** crystal modification **V**. Six API salts (**cGMPSA‐Na**, **‐Ca**, **‐NH_4_**, **‐Tris**, **‐Bnet**, **‐BZ**) as well as the free acid form (**cGMPSA‐H**) were prepared from **cGMPSA‐TEA**. A total of 21 crystal modifications were found and characterized by XRPD and catalogued. According to all thermal analyses, no API forms exhibit any melting points, and instead a temperature of decomposition is reached before melting. All API forms were relatively thermally stable, with the lowest onset of decomposition observed for **cGMPSA‐H** at ~163 °C. Aqueous solubility studies were undertaken, and **cGMPSA‐Ca**, **‐Bnet**, and **‐BZ** were found to have the lowest aqueous solubilities at 280, 50, and 10 μg/mL, respectively, which could help in sustaining drug release in drug formulations. The highest solubilities were observed for the **‐Na** and **‐Tris** salts, at approximately 40 and 15 mg/mL, respectively. **cGMPSA** hygroscopicity as measured by DVS could be modified by selection of different salts and polymorphs. A number of hydrates and solvates were identified for several salts studied. The preparations and crystallizations for four API crystal modifications, **cGMPSA‐Ca III**, **‐Na III**, **‐Bnet II**, and **‐BZ II**, were developed and upscaled to approximately 10 g quantities to enable future formulation studies.

## Materials and Methods

### Materials

All **cGMPSA** forms were prepared from the triethylammonium salt according to the protocols described in the Supporting Information. The starting triethylammonium salt was obtained according to the procedure reported in the literature.^[2]^

### X‐Ray Powder Diffraction (XRPD)

XRPD analyses were performed at 20 °C on a PANalytical X′Pert PRO instrument, equipped with a Cu X‐ray tube and a PIXcel detector. Automatic divergence and antiscatter slits were used together with 0.02 rad Soller slits and a Ni filter. Solid samples were analyzed on cut silicon zero background holders (ZBH). Slurry samples were dripped on porous alumina substrates, which produce peaks at 2θ=25.6°, 35.0°, and 37.7°. The randomness of the analysis was increased by spinning them during the analysis. Samples were analyzed between 2θ=2 and 40° over 17 min.

### Single‐Crystal X‐Ray Diffraction (SXRD)

A suitable crystal for single‐crystal X‐Ray Diffraction (SXRD) was obtained from a solvent‐antisolvent layering experiment using **cGMPSA‐TEA** as the starting material and EtOH and Et_2_O as the solvent and antisolvent, respectively. The starting solution (approximately 3.5 mM) was pipetted into an NMR vial, followed by carefully layering 2 parts of Et_2_O on top, and allowing it to passively diffuse into the lower phase. The crystal was then mounted on a cryoloop using Paratone oil. Single‐crystal X‐ray data was collected at 296 K using a Bruker APEXII diffractometer (Mo Kα radiation), equipped with a CCD detector. ω Scans and φ scans were used to record the data sets, which subsequently were integrated with the Bruker SAINT software package. Absorption correction (Bruker SADABS) was based on the fitting of a function to the empirical transmission surface, as sampled by multiple equivalent measurements. SHELXS and SHELXL (within the Bruker program package) were used for solution and refinement of the crystal structures. Structure solution by direct methods resolved positions of all atoms except hydrogens. Hydrogen atoms were placed at calculated positions, except hydrogen atoms of the water which are unassigned. Depending on cation disorder some carbon atoms are refined isotropic and their hydrogen atoms are unassigned. Deposition Number 2164547 contains the supplementary crystallographic data for this paper. These data are provided free of charge by the joint Cambridge Crystallographic Data Centre and Fachinformationszentrum Karlsruhe Access Structures service. Crystal data: M_r_=1329.89, monoclinic, space group *P*2_1_ (no. 4), a=12.8018(10) Å, b=16.5214(13) Å, c=14.8692(12) Å, β=110.072(5)°, V=2953.9(4) Å^3^, Z=2, D_calc_=1.495 g×cm^−3^, μ(MoKα)=1.570 mm^−1^, R=0.0494, wR_2_=0.1208, GooF=1.015, Flack x=0.023(3).

### Thermal analysis

Differential scanning calorimetry (DSC) analyses were carried out on a Mettler DSC822e, equipped with a Thermo Haake EK45/MT cooler. The samples were weighed into a 40 μL Al‐cup, which were then closed with a pierced lid. The sample was scanned from 25 to 300 °C, with a scan rate of 10 °C/min.

Thermogravimetric analysis (TGA) was performed on a Mettler TGA/SDTA 851e, equipped with a Haake C50P cooler. Alternatively, thermogravimetric and DSC analyses were carried out simultaneously in a Mettler Toledo TGA/DSC 3+. The samples were weighed into a 100 μL Al‐cup and this was flushed with dry nitrogen gas during the analysis. The sample was scanned from 25 to 300 °C, with a scan rate of 10 °C/min.

Hot‐stage optical microscopy was performed on a Mettler Toledo HS82 hot‐stage system with an internal furnace and a HS1 Hot Stage control unit. The sample was heated from 25 to 300 °C at a rate of 10 K/min and monitored under an Olympus BX51 light‐polarizing microscope coupled with a PixeLINK PL‐A662 digital camera.

### Dynamic Vapor Sorption (DVS)

The samples were analyzed at 25 °C with a Surface Measurements Systems DVS Advantage. A sample was weighted into a weighing pan and its mass was monitored while it was exposed to different relative humidities (%RH=%P/P0). Before the analysis, the weighing pan was subject to an antistatic program.

The analysis program consisted of two cycles. In each cycle, the relative humidity was increased stepwise up to 95 % RH (starting from 0 % RH, 9 steps with 10 % and one step with 5 %) and then decreased stepwise to 0 % RH again. In both sorption/desorption cycles, at each step, the sample was kept at the set relative humidity until dm/dt <0.002 %, over 15 min.

### Nuclear Magnetic Resonance (NMR) Spectroscopy

All NMR spectra were recorded on a Bruker AV 500 MHz (500.13 MHz in ^1^H, 125.76 MHz in ^13^C, and 202.47 MHz in ^31^P) spectrometer using the given deuterated solvent as an internal standard. Chemical shifts (δ scale) are reported in parts per million (ppm), and coupling constants (*J* values) are reported in Hertz (Hz).

### Solubility studies and polymorph screening

The pH‐solubility profile of **cGMPSA** salts and free acid was determined by phase solubility techniques.^[18]^ Briefly, saturated mixtures of the compound were prepared by adding excess amount to 4 mL vials containing 2 mL deionized water, an acidic buffer (0.1 M HCl_(aq)_, pH 1), or a basic buffer (0.1 M NaOH_(aq)_, pH 13). They were allowed to equilibrate for 24 h using mild agitation before recording the pH. The clear solution was separated from the solids by filtration with a Cytiva Whatman™ Mini‐Uniprep™ polypropylene 0.45 μm filter device and diluted to an appropriate analytical concentration where needed. The sample was then analyzed by HPLC, and the solubility determined by integrating the area of the peak of interest against a standard curve. The solids were also analyzed by XRPD to record the crystal modification obtained.

A polymorph screen was done for **cGMPSA‐TEA** with a similar approach. An excess of the compound was slurried in a range of solvents, typically 20–40 mg in 1 mL. For screens where enough solid material was obtained, it was analyzed by XRPD to record the crystal modification. An approximate solubility was obtained by sampling the clear solution on UHPLC and comparing the peak area against that of a 40 mg/mL reference solution of the compound in DMSO.

### Ultra‐High Performance Liquid Chromatography (UHPLC)

UHPLC analysis was carried out on a Thermo Fisher Scientific Vanquish VH‐10‐A UHPLC equipped with a VH‐D10‐A photo diode array detector and an ISQ™ EM Single Quadrupole Mass Spectrometer. UV detection was taken at 210 nm, and mass detection was between 100–1000 in both negative and positive mode. An Acquity Premiere BEH C18 column (50×2 mm, 1.7 μm particle size) was used with a 0.4 mL/min flow rate at 40 °C, and a linear gradient of 2–100 % of buffer B in buffer A over 3.8 minutes at 40 °C. The buffers were: 5 mM ammonium acetate (A); and MeCN (B).

## Supporting Information

Synthetic methods and analytical data for API salts and some of their crystal modifications, including ^1^H NMR, ^31^P NMR, ^31^P{^1^H} NMR, and ^19^F{^1^H} NMR spectra, XRPD, DSC‐TGA, DVS, and pH‐dependent solubility data.

## Conflict of interest

The authors declare no conflict of interest.

1

## Supporting information

As a service to our authors and readers, this journal provides supporting information supplied by the authors. Such materials are peer reviewed and may be re‐organized for online delivery, but are not copy‐edited or typeset. Technical support issues arising from supporting information (other than missing files) should be addressed to the authors.

Supporting Information

## Data Availability

The data that support the findings of this study are available in the supplementary material of this article.
