# Supplementary material for: Crystal Modifications of a Cyclic Guanosine Phosphorothioate Analogue, a Drug Candidate for Retinal Neurodegenerations
Source: ChemistryOpen. 2023 Oct 25;12(12):e202300141. doi: 10.1002/open.202300141 (PMC10695737; doi:10.1002/open.202300141)
Supplement: Supplementary file 1 — Supporting Information [file OPEN-12-e202300141-s001.pdf]

ChemistryOpen

Supporting Information

Crystal Modifications of a Cyclic Guanosine Phosphorothioate Analogue, a Drug Candidate for Retinal Neurodegenerations

Oswaldo Pérez,* Nicolaas Schipper,* Valentina Leandri, Per H. Svensson, Martin Bohlin, Thorsteinn Loftsson, and Martin Bollmark

Table of Contents

Triethylammonium R_P-8-Bromo-β-phenyl-1,N^2-ethenoguanosine-3′,5′-cyclicmonophosphorothioate (cGMPSA-TEA) 3
HPLC-UV-MS 4
NMR 5
XRPD 10
DSC & TGA 11
DVS 12
cGMPSA-salt formations 13
Sodium R_P-8-Bromo-β-phenyl-1,N^2-ethenoguanosine-3′,5′-cyclicmonophosphorothioate (cGMPSA-Na) 13
HPLC-UV-MS 14
NMR 15
XRPD 17
DSC & TGA 18
DVS 19
R_P-8-Bromo-β-phenyl-1,N^2-ethenoguanosine-3′,5′-cyclicmonophosphorothiotic acid (cGMPSA-H) 20
HPLC-UV-MS 21
NMR 22
XRPD 24
DSC & TGA 24
DVS 25
Calcium R_P-8-Bromo-β-phenyl-1,N^2-ethenoguanosine-3′,5′-cyclicmonophosphorothioate (1:2) (cGMPSA-Ca) 26
HPLC-UV-MS 27
NMR 28
XRPD 30
DSC & TGA 31
DVS 32
Ammonium R_P-8-Bromo-β-phenyl-1,N^2-ethenoguanosine-3′,5′-cyclicmonophosphorothioate (cGMPSA-NH_4) 33
HPLC-UV-MS 34
NMR 35
DSC & TGA 37

Tris(hydroxymethyl)aminomethane R_P-8-Bromo-β-phenyl-1,N^2-ethenoguanosine-3′,5′-cyclicmonophosphorothiotic acid (cGMPSA-Tris) 38
HPLC-UV-MS 39
NMR 40
DSC & TGA 42
DVS 43
XRPD (cGMPSA-NH_4 and cGMPSA-Tris) 44
N-(Phenylmethyl)benzeneethanammonium R_P-8-Bromo-β-phenyl-1,N^2-ethenoguanosine-3′,5′-cyclicmonophosphorothioate (cGMPSA-Bnet) 45
HPLC-UV-MS 46
NMR 47
DSC & TGA 49
DVS 50
N,N′-Dibenzylethylenediammonium R_P-8-Bromo-β-phenyl-1,N^2-ethenoguanosine-3′,5′-cyclicmonophosphorothioate (1:2) (cGMPSA-BZ) 51
HPLC-UV-MS 52
NMR 53
DSC & TGA 55
DVS 56
XRPD (cGMPSA-Bnet and cGMPSA-BZ) 57
Aqueous solubility measurements 58
pH-Dependent aqueous solubility measurements. 59
Single Crystal X-ray Diffraction 60
References 64

Triethylammonium *R*P-8-Bromo-β-phenyl-1,*N*²-ethenoguanosine-3′,5′-cyclicmonophosphorothioate (cGMPSA-TEA)

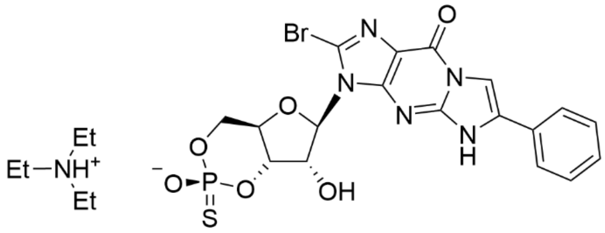

Preparation and characterization of the parent compound **cGMPSA-TEA** is found in work previously published by some of the authors.[1]

HPLC-UV-MS

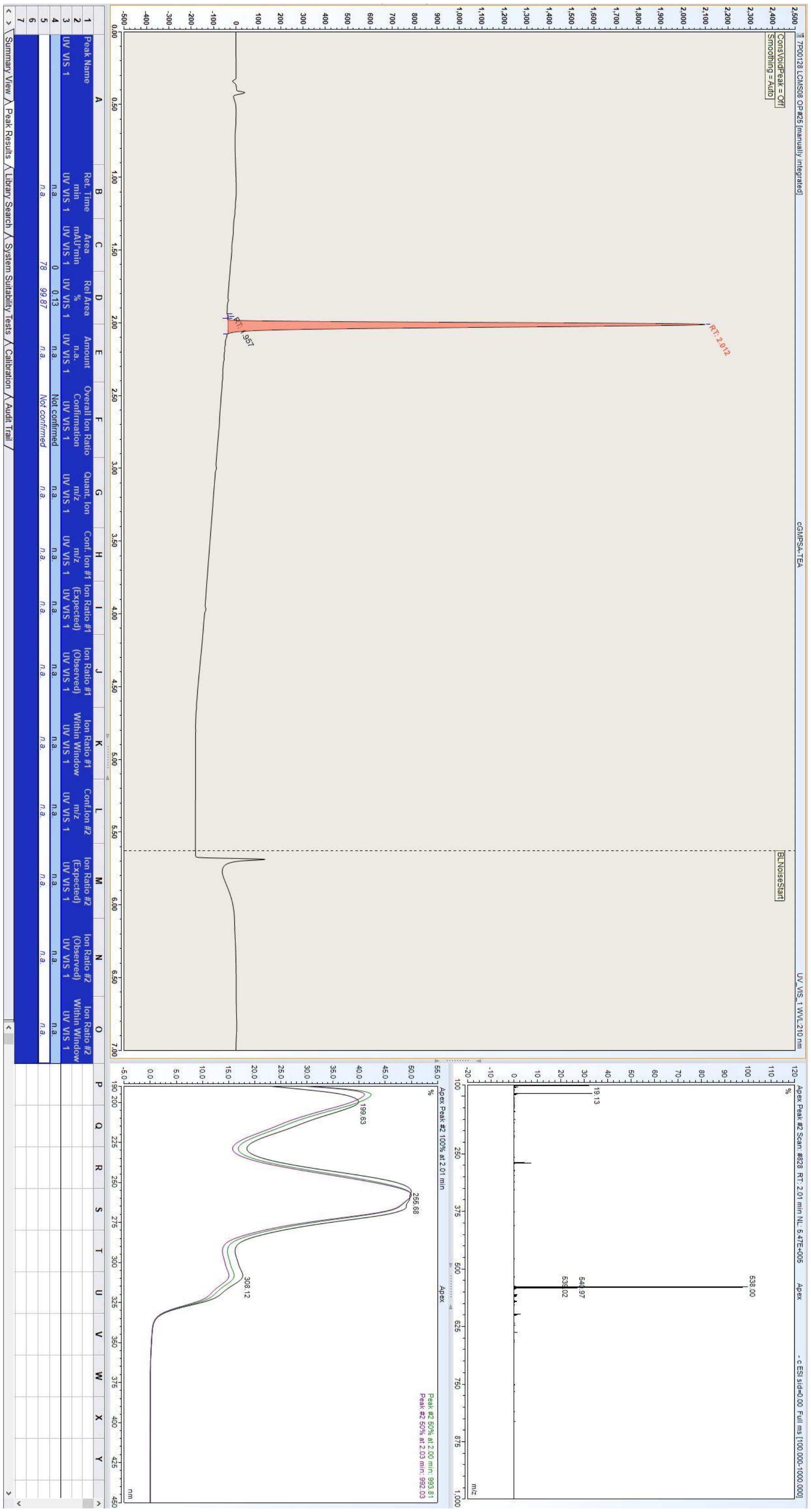

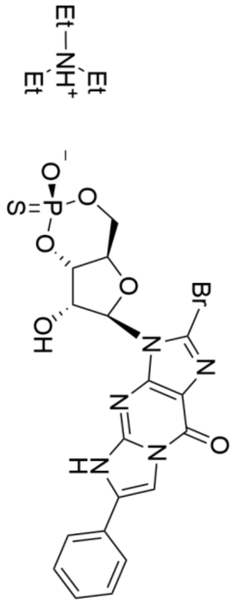

NMR

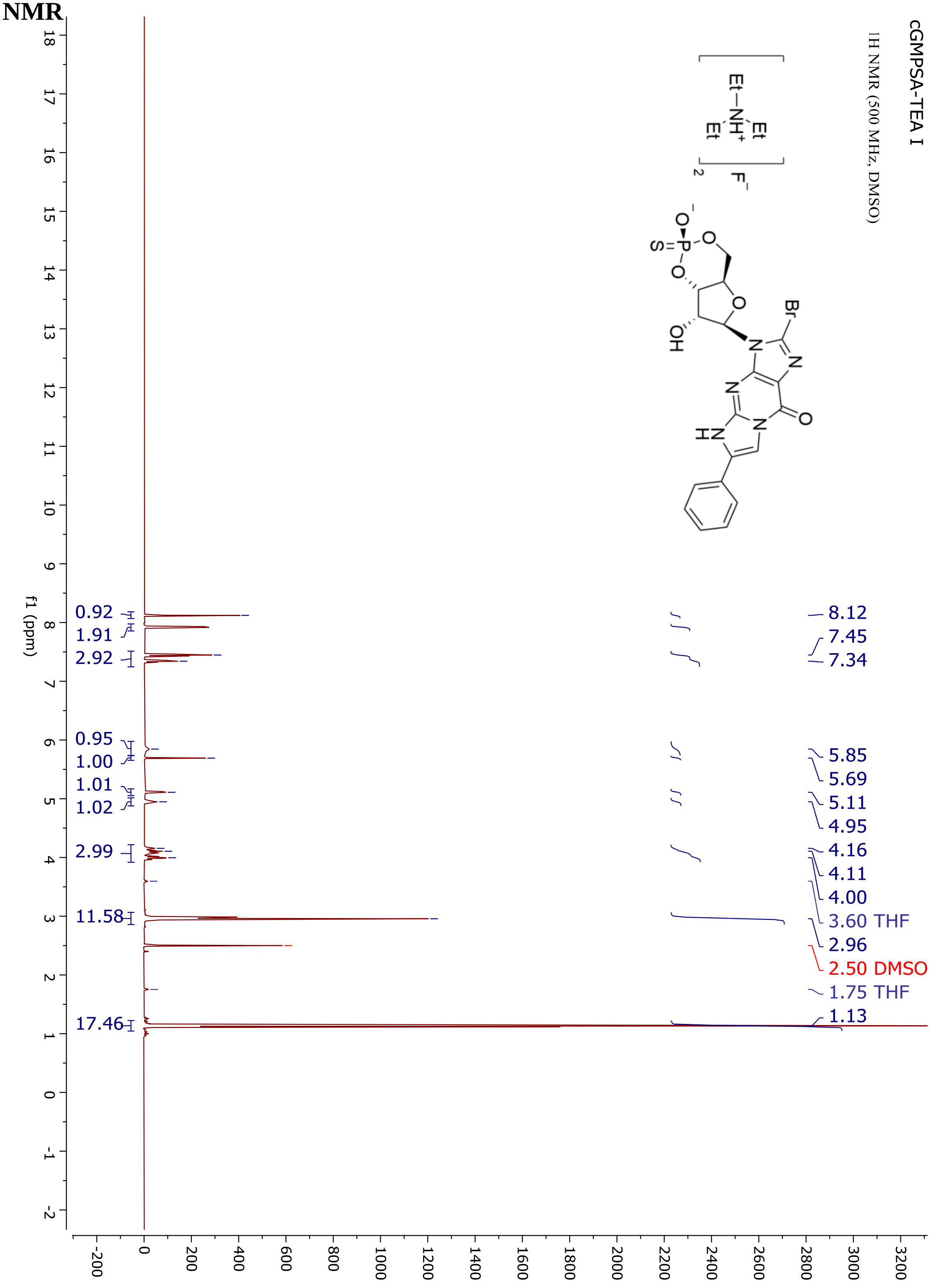
cGMPSA-TEA I
1H NMR (500 MHz, DMSO)
8.12
7.45
7.34
5.85
5.69
5.11
4.95
4.16
4.11
4.00
3.60 THF
2.96
2.50 DMSO
1.75 THF
1.13
0.92
1.91
2.92
0.95
1.00
1.01
1.02
2.99
11.58
17.46
f1 (ppm)

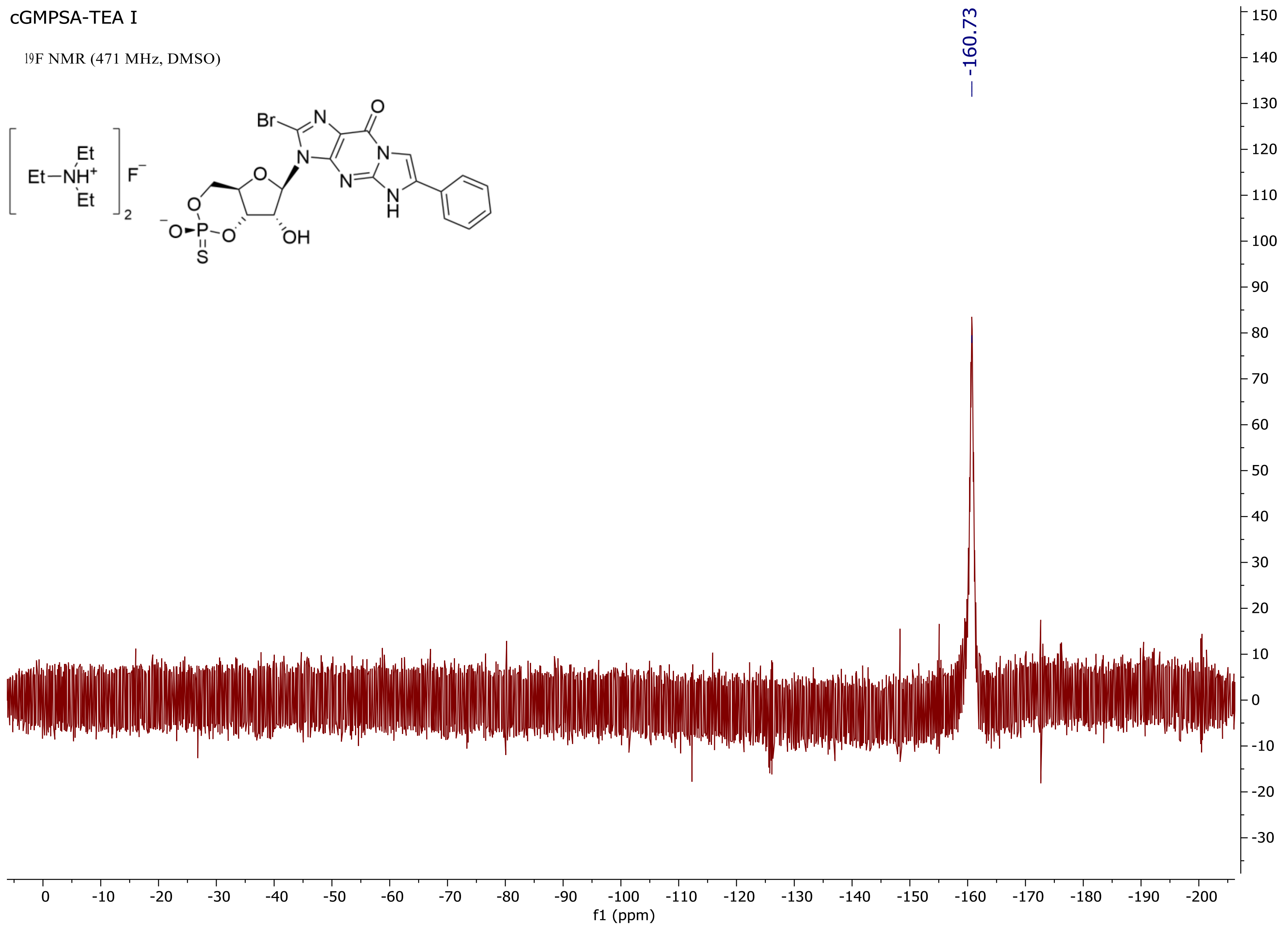
cGMPSA-TEA I
19F NMR (471 MHz, DMSO)
-160.73
f1 (ppm)

cGMPSA-TEA II

1H NMR (500 MHz, DMSO)

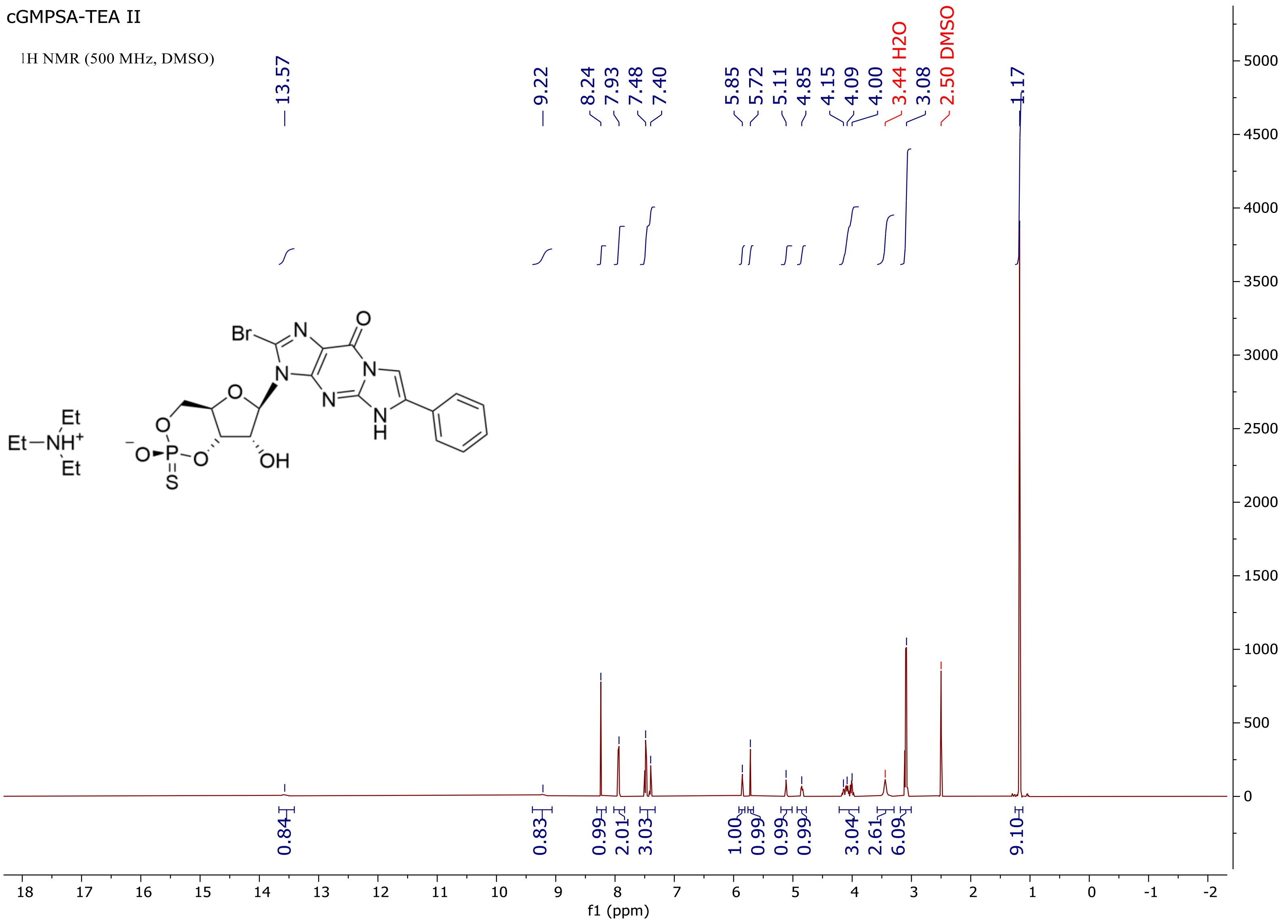

cGMPSA-TEA II

19F NMR (471 MHz, DMSO)

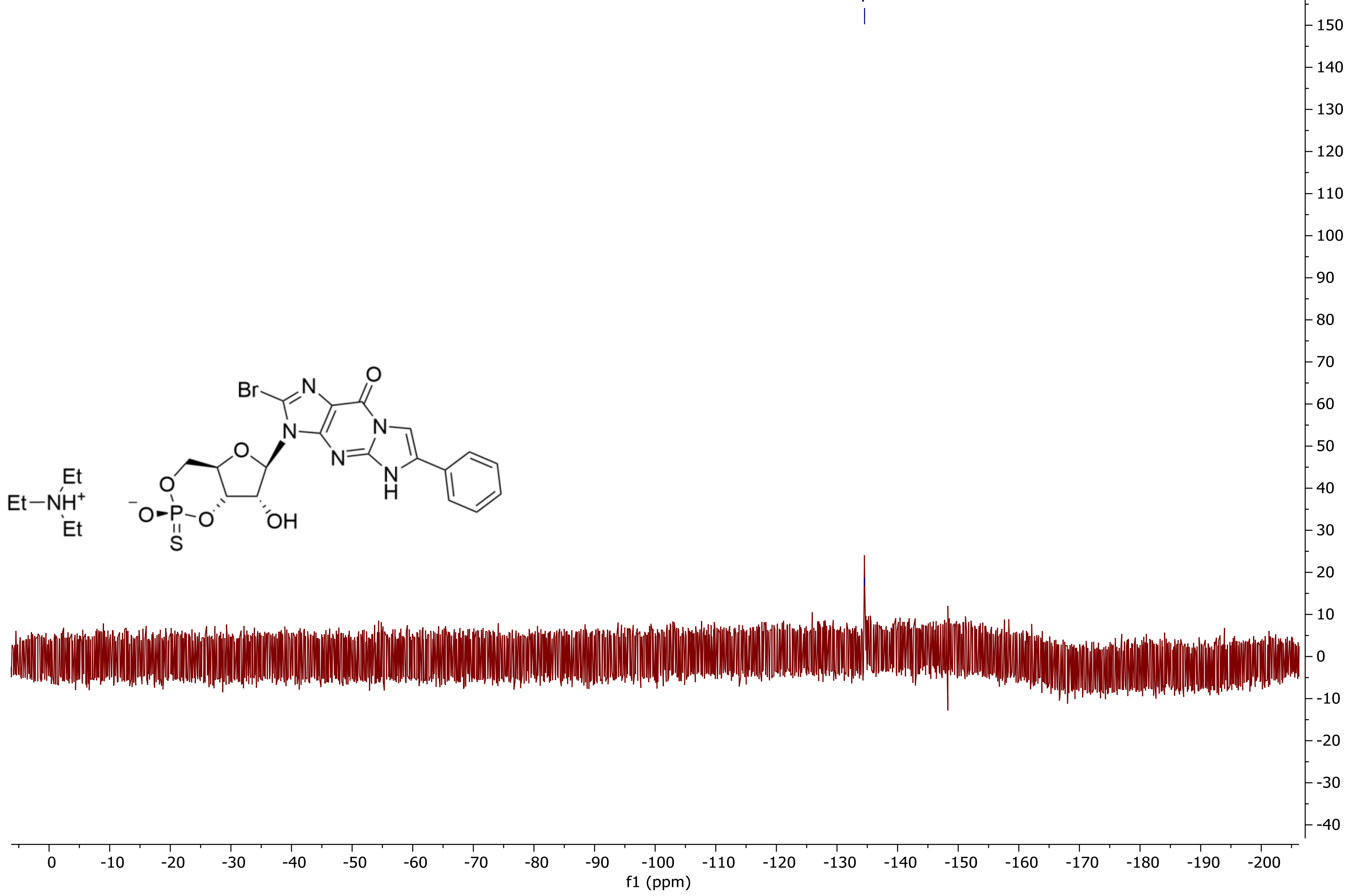

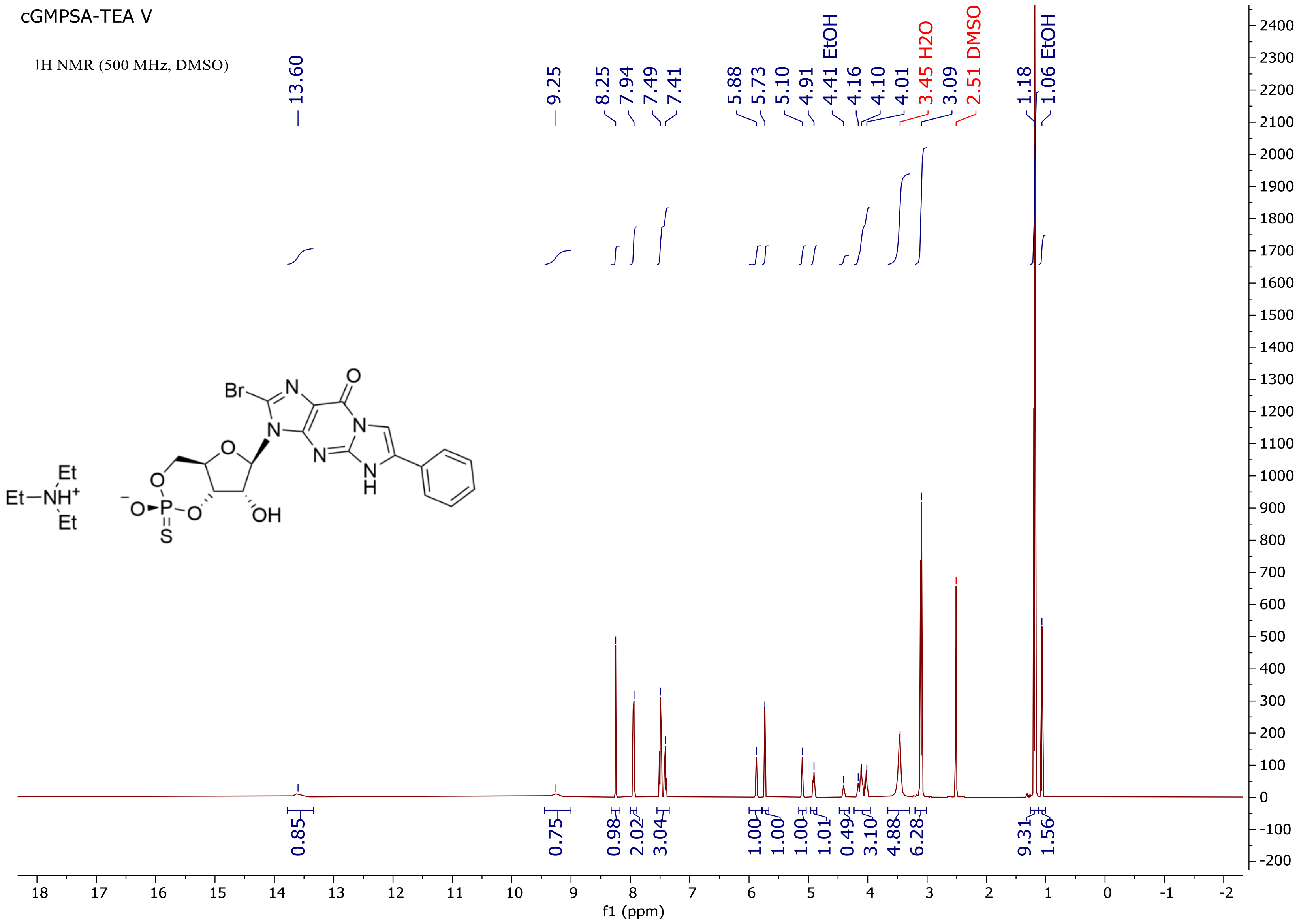
cGMPSA-TEA V
1H NMR (500 MHz, DMSO)
13.60
9.25
8.25
7.94
7.49
7.41
5.88
5.73
5.10
4.91
4.41 EtOH
4.16
4.10
4.01
3.45 H2O
3.09
2.51 DMSO
1.18
1.06 EtOH
0.85
0.75
0.98
2.02
3.04
1.00
1.00
1.00
1.01
0.49
3.10
4.88
6.28
9.31
1.56
Br
N
N
O
N
N
N
H
O
O
P
O
S
OH
Et
Et—NH+
Et
f1 (ppm)

XRPD

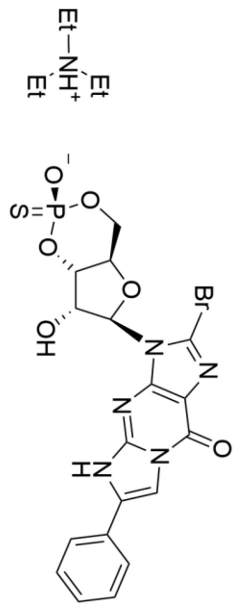

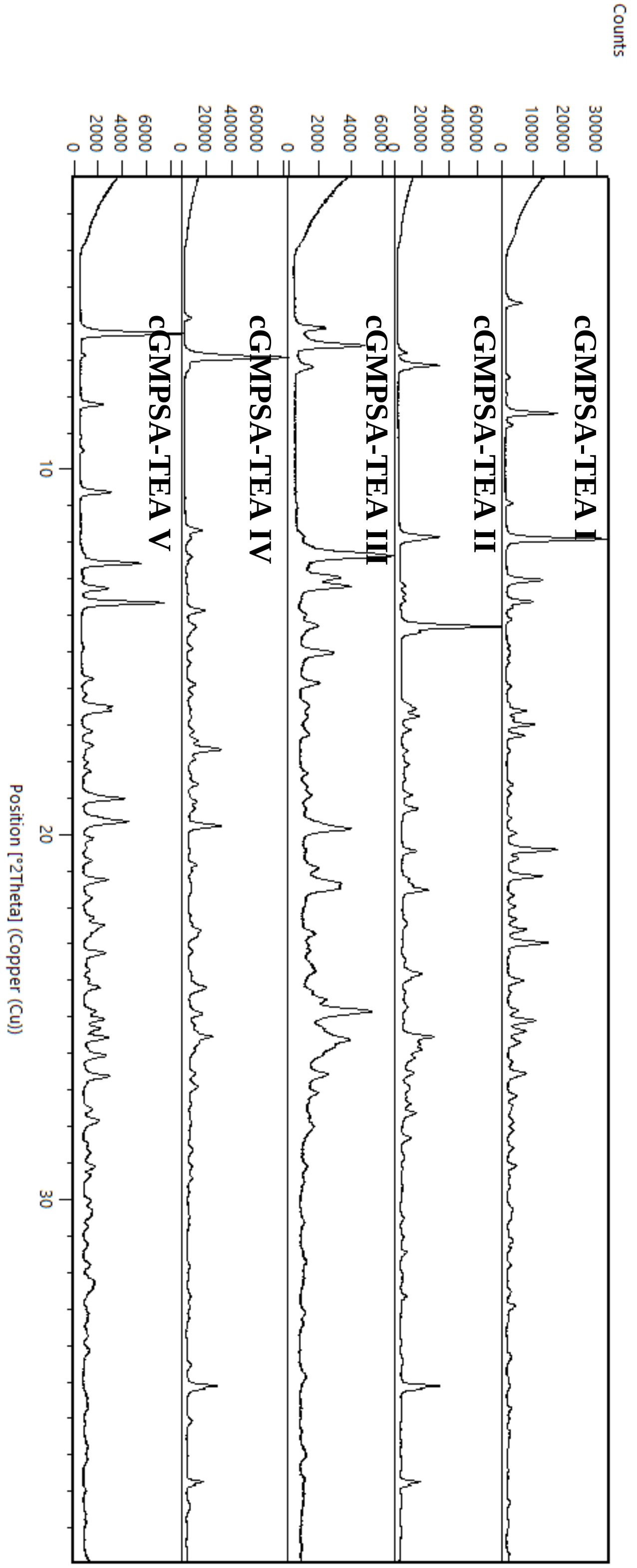
Counts
cGMPSA-TEA I
cGMPSA-TEA II
cGMPSA-TEA III
cGMPSA-TEA IV
cGMPSA-TEA V
Position [°2Theta] (Copper (Cu))

DSC & TGA

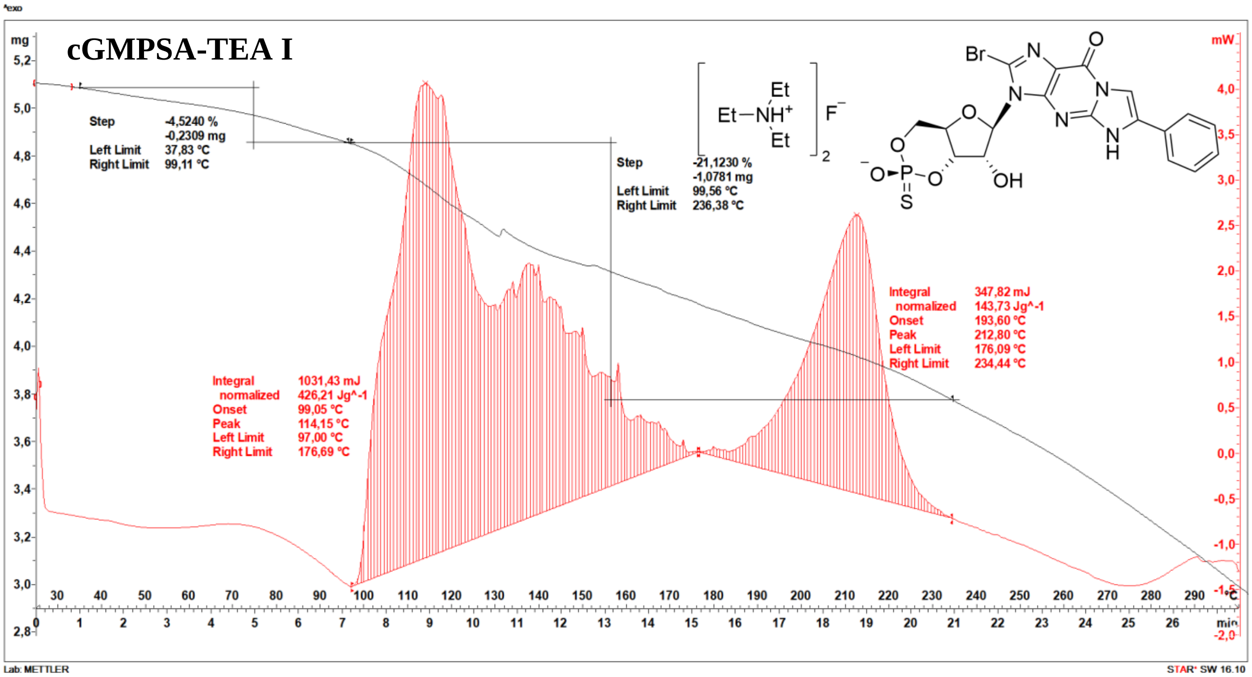
cGMPSA-TEA I
Step -4,5240 % -0,2309 mg
Left Limit 37,83 °C
Right Limit 99,11 °C
Step -21,1230 % -1,0781 mg
Left Limit 99,56 °C
Right Limit 236,38 °C
Integral 1031,43 mJ
normalized 426,21 Jg^-1
Onset 99,05 °C
Peak 114,15 °C
Left Limit 97,00 °C
Right Limit 176,69 °C
Integral 347,82 mJ
normalized 143,73 Jg^-1
Onset 193,60 °C
Peak 212,80 °C
Left Limit 176,09 °C
Right Limit 234,44 °C
Lab: METTLER
STARe SW 16.10

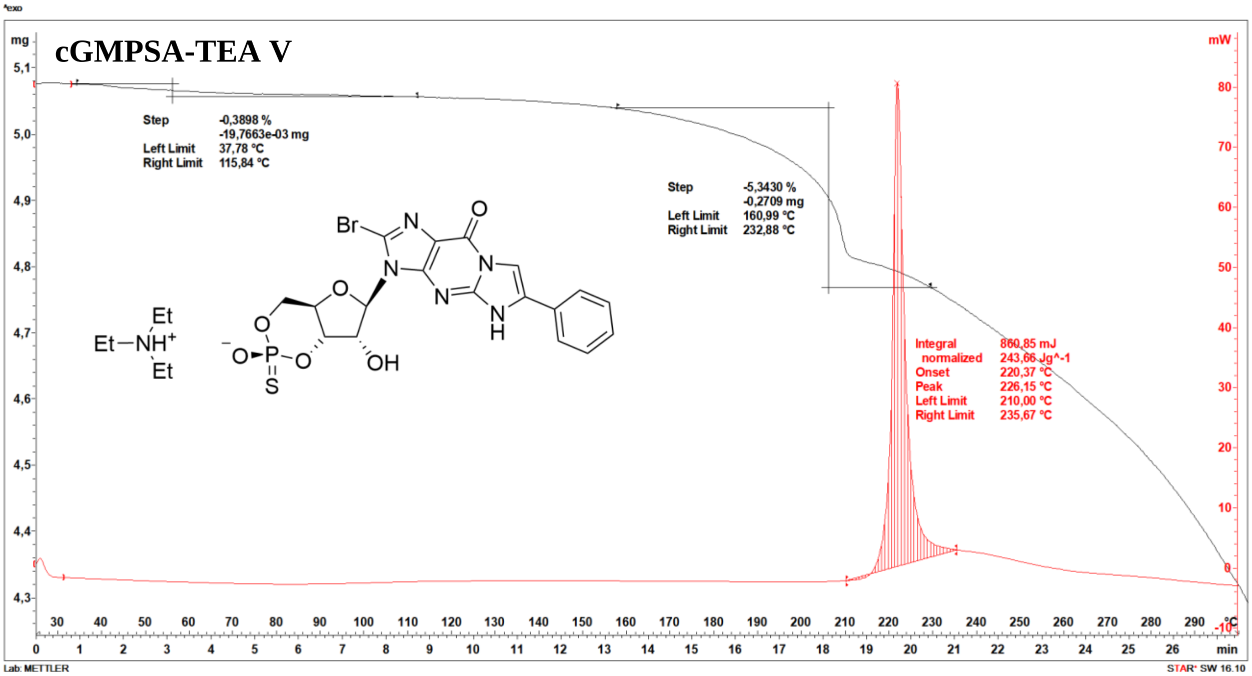
cGMPSA-TEA V
Step -0,3898 % -19,7663e-03 mg
Left Limit 37,78 °C
Right Limit 115,84 °C
Step -5,3430 % -0,2709 mg
Left Limit 160,99 °C
Right Limit 232,88 °C
Integral 860,85 mJ
normalized 243,66 Jg^-1
Onset 220,37 °C
Peak 226,15 °C
Left Limit 210,00 °C
Right Limit 235,67 °C
Lab: METTLER
STARe SW 16.10

DVS

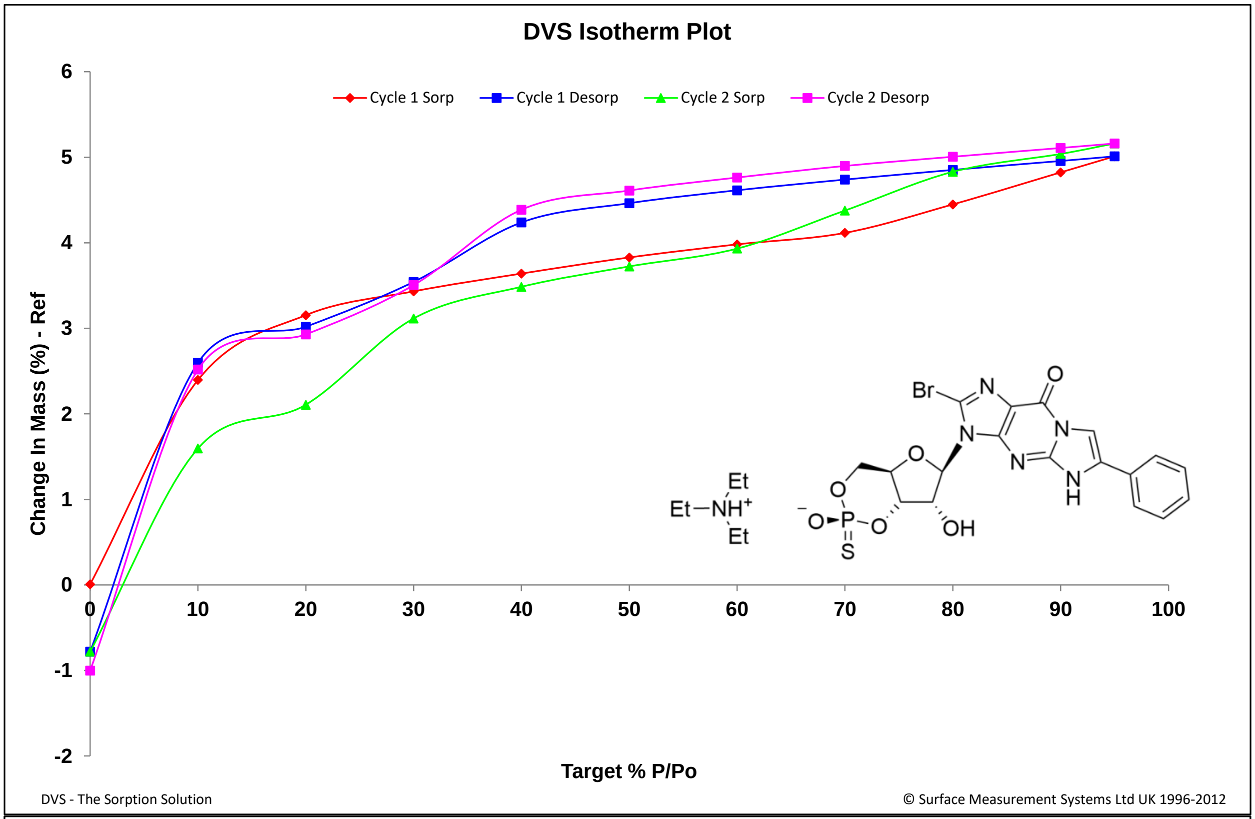
DVS Isotherm Plot
Cycle 1 Sorp
Cycle 1 Desorp
Cycle 2 Sorp
Cycle 2 Desorp
Change In Mass (%) - Ref
Target % P/Po
DVS - The Sorption Solution
© Surface Measurement Systems Ltd UK 1996-2012

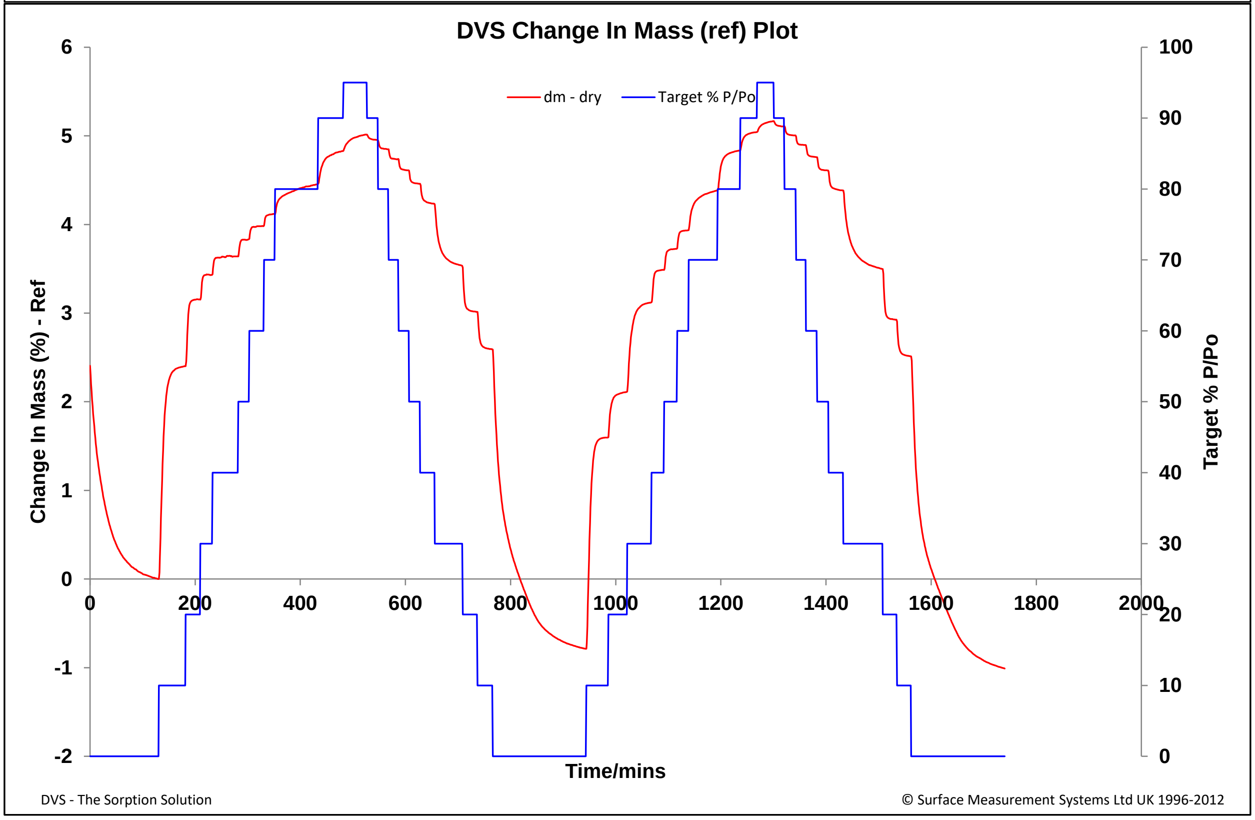
DVS Change In Mass (ref) Plot
dm - dry
Target % P/Po
Change In Mass (%) - Ref
Target % P/Po
Time/mins
DVS - The Sorption Solution
© Surface Measurement Systems Ltd UK 1996-2012

cGMPSA-salt formations

The starting triethylammonium counter-ion was changed to the target counter-ion according to the following procedures.

Sodium *R*P-8-Bromo-β-phenyl-1,*N*²-ethenoguanosine-3′,5′-cyclicmonophosphorothioate (cGMPSA-Na)

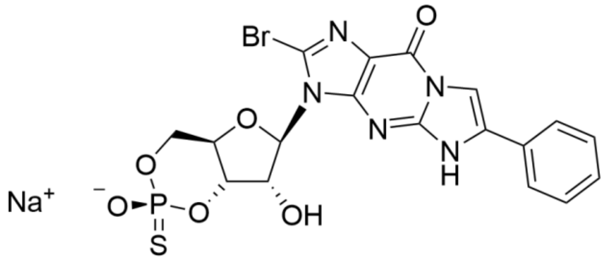

cGMPSA-TEA (15 g, 23.4 mmol) was stirred in refluxing MeOH (150 mL, 10 volumes) until all or most of the starting material was dissolved. Sodium 2-ethylhexanoate (2 equiv., 7.8 g, 46.8 mmol) was charged into the vessel in one portion, which shortly dissolved. A white slurry quickly formed. The mixture was stirred for 1 hour before acclimating to RT and stirred for 1 further hour at RT. The white solid was filtered and washed with MeOH (2 x 10 mL), followed by drying at 50°C under a vacuum. Yield: 84% (11.0 g, 19.7 mmol). HPLC purity: >99.9%. ^{1}H NMR (500 MHz, DMSO-d_6): δ 13.58 (br s, 1H), 8.23 (s, 1H), 7.98–7.90 (m, 2H), 7.52–7.45 (m, 2H), 7.43–7.36 (m, 1H), 5.83 (d, J = 4.9 Hz, 1H), 5.71 (d, J = 1.6 Hz, 1H), 5.12–5.07 (m, 1H), 4.91–4.84 (m, 1H), 4.20–4.07 (m, 2H), 4.02–3.95 (m, 1H). ^{13}C NMR (126 MHz, DMSO-d_6): δ 150.4, 150.0, 145.9, 129.6, 129.0, 128.8, 127.8, 125.2, 122.8, 116.1, 103.6, 93.4, 75.1 (d, J_{PC} = 6.4 Hz, 1C), 71.6 (d, J_{PC} = 5.3 Hz, 1C), 69.6 (d, J_{PC} = 7.1 Hz, 1C), 65.32 (d, J_{PC} = 9.0 Hz, 1C). MS (M – Na^{+}) *m*/*z*: 537.96 calcd for $C_{18}H_{14}BrN_5O_6PS^-$; found, 537.97 (ES^{-}). DSC (exotherm, onset): 211.8 °C.

HPLC-UV-MS

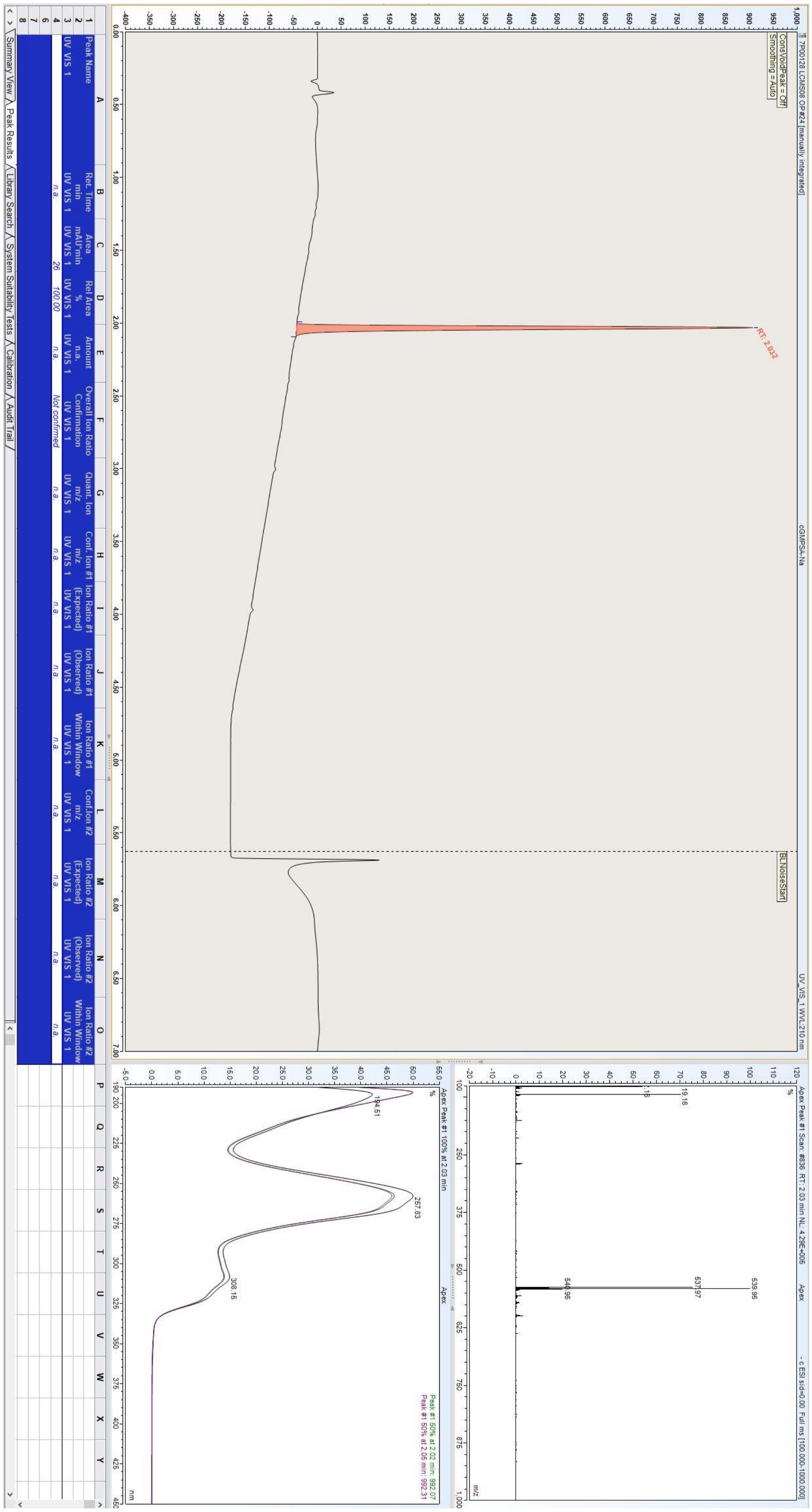

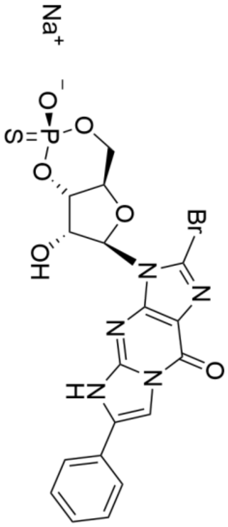

NMR

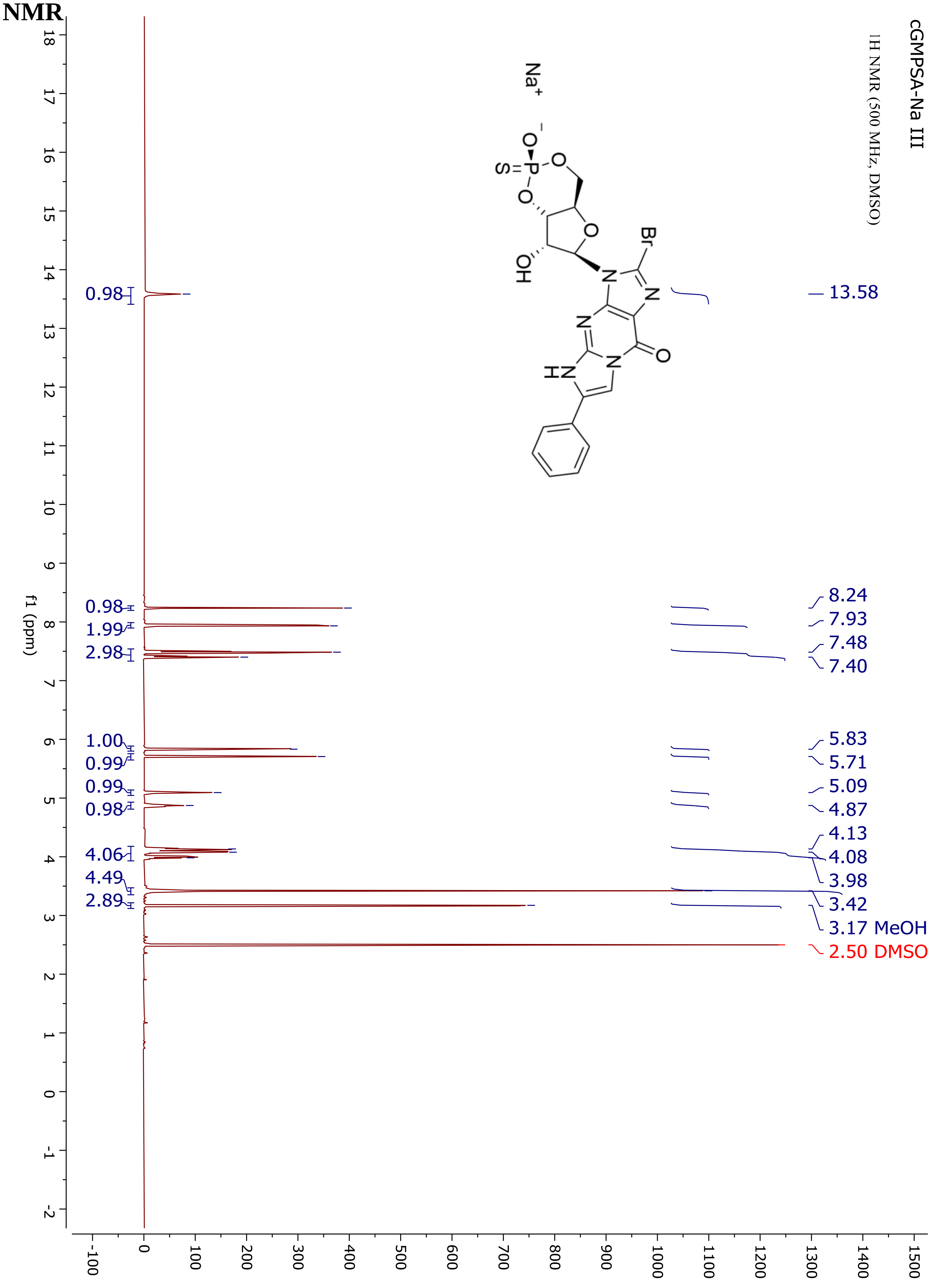

cGMPSA-Na III
1H NMR (500 MHz, DMSO)
Na+
13.58
8.24
7.93
7.48
7.40
5.83
5.71
5.09
4.87
4.13
4.08
3.98
3.42
3.17 MeOH
2.50 DMSO
0.98
0.98
1.99
2.98
1.00
0.99
0.99
0.98
4.06
4.49
2.89
f1 (ppm)

cGMPSA-Na

13C NMR (126 MHz, DMSO)

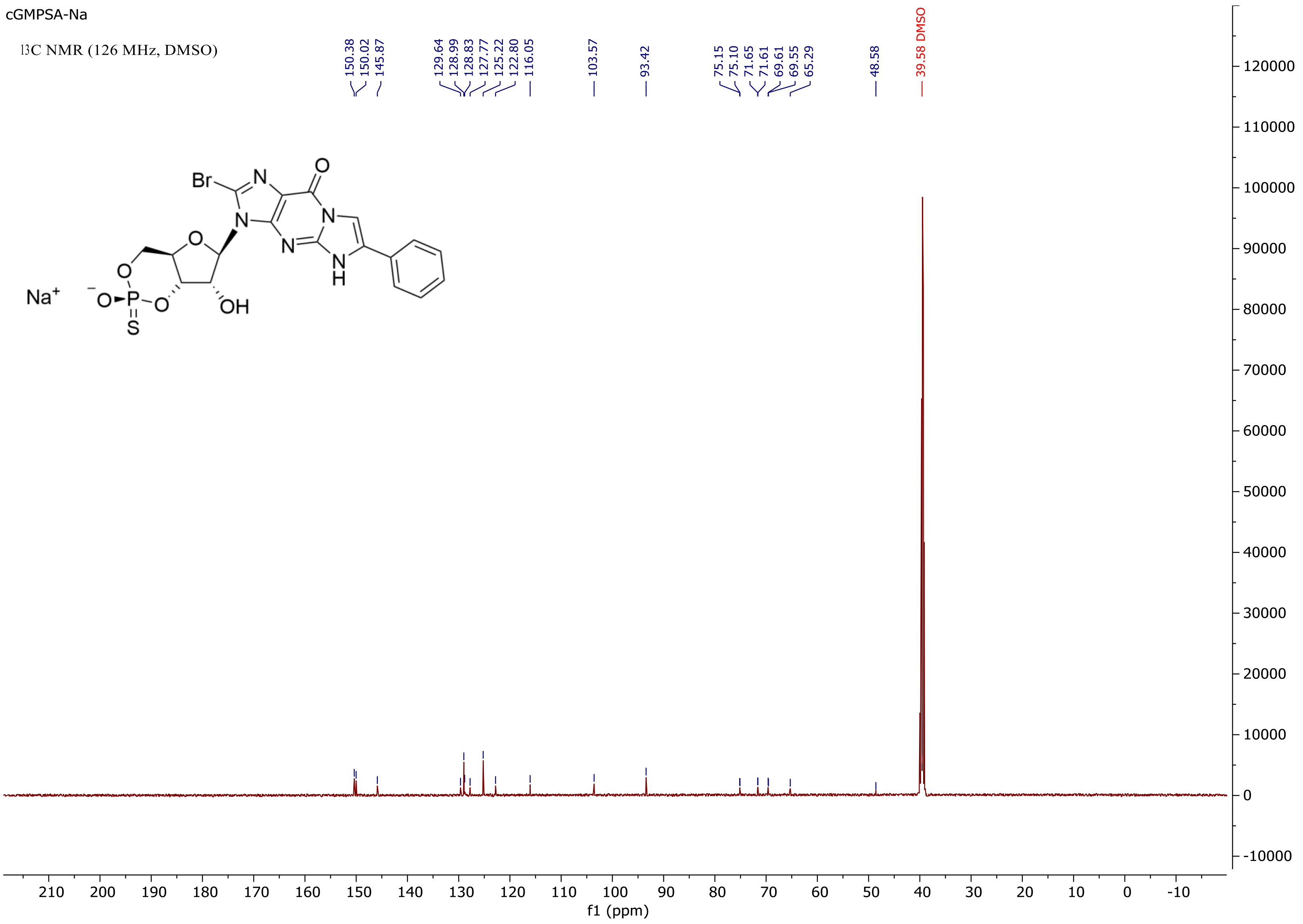

XRPD

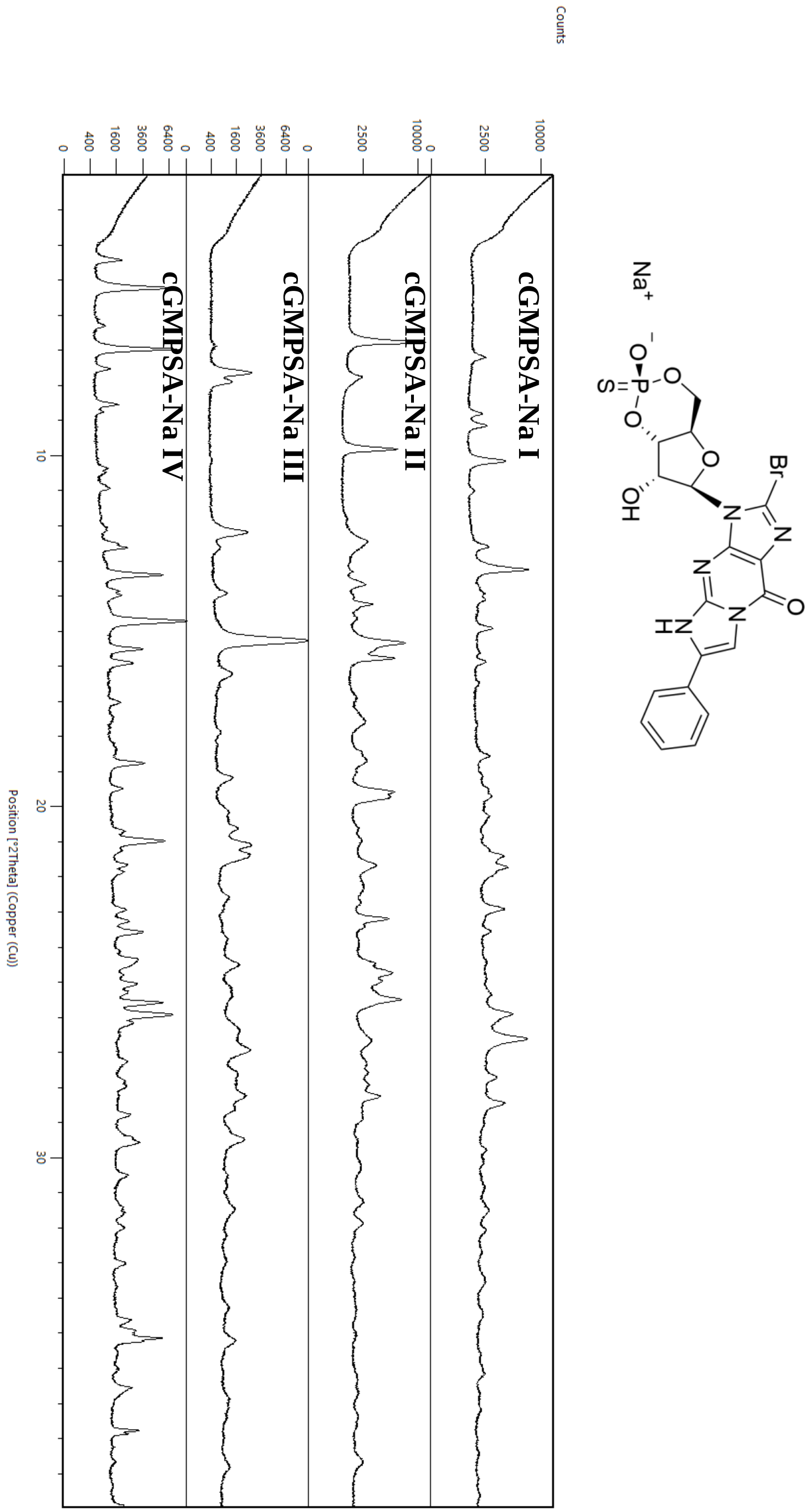
Counts
cGMPSA-Na I
cGMPSA-Na II
cGMPSA-Na III
cGMPSA-Na IV
Position [°2Theta] (Copper (Cu))
10
20
30
Na+
Br
OH

DSC & TGA

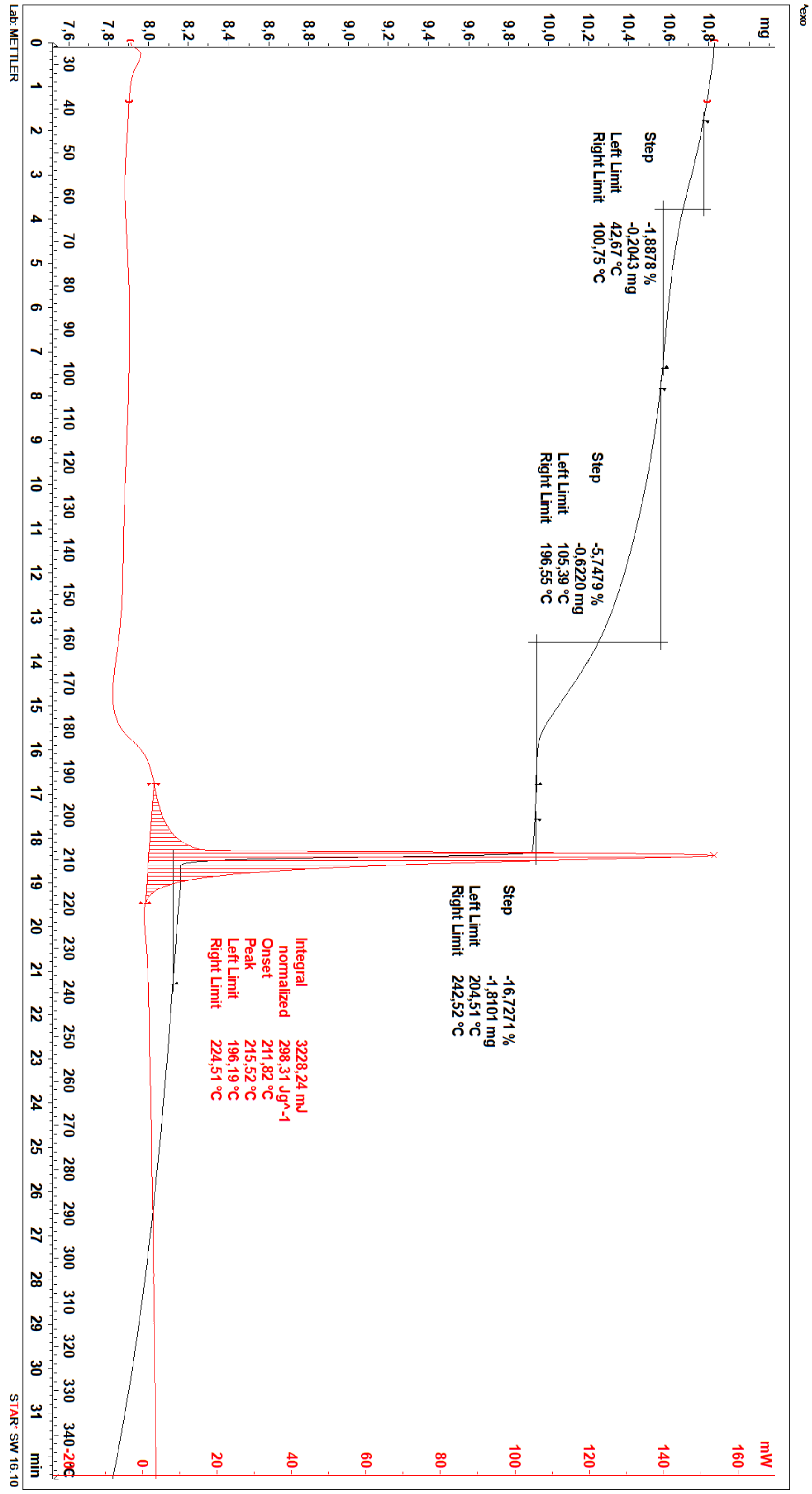
Step
-1,8878 %
-0,2043 mg
Left Limit 42,67 °C
Right Limit 100,75 °C
Step
-5,7479 %
-0,6220 mg
Left Limit 105,39 °C
Right Limit 196,55 °C
Step
-16,7271 %
-1,8101 mg
Left Limit 204,51 °C
Right Limit 242,52 °C
Integral 3228,24 mJ
normalized 298,31 Jg^-1
Onset 211,82 °C
Peak 215,52 °C
Left Limit 196,19 °C
Right Limit 224,51 °C
Lab: METTLER
STARe SW 16.10

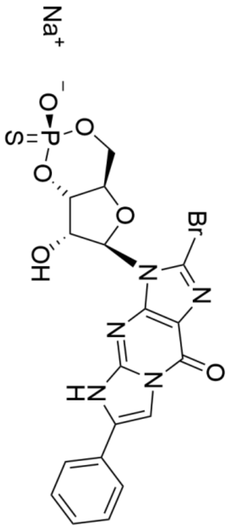

DVS

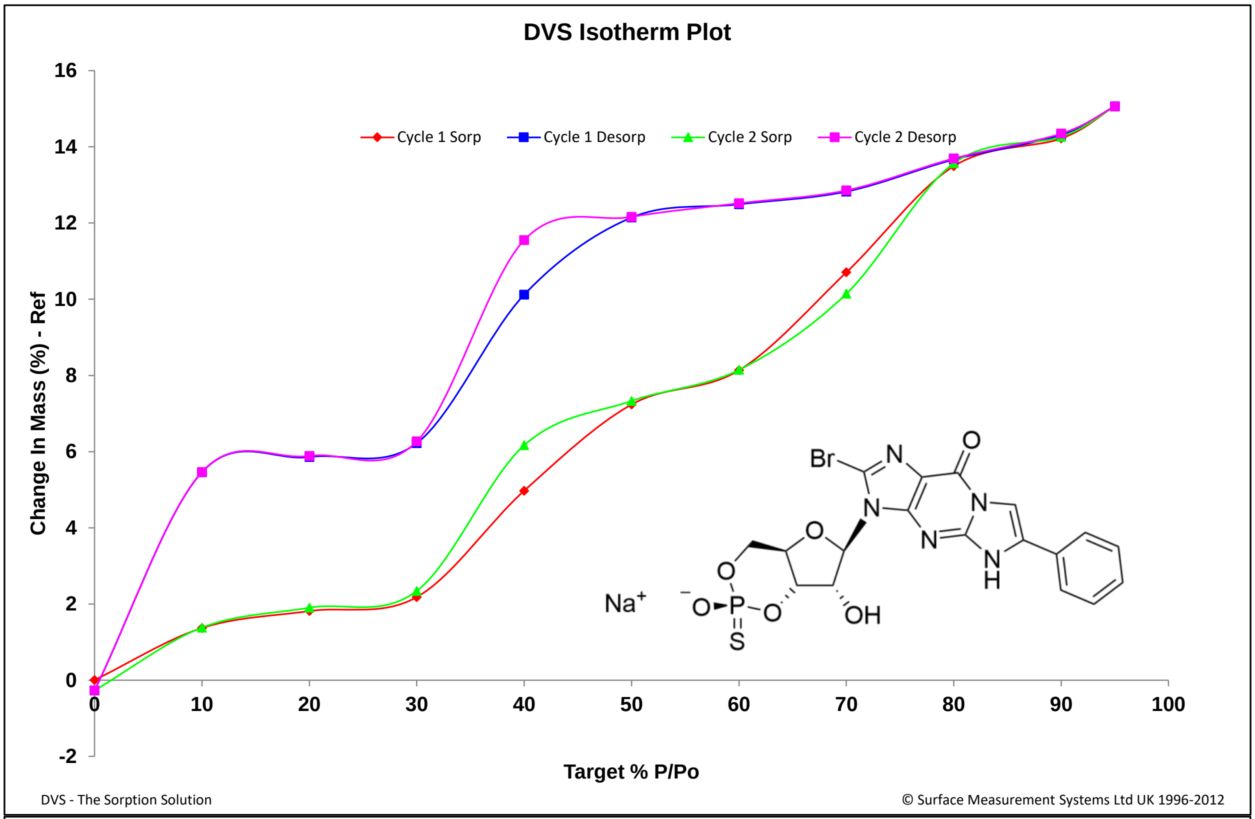

DVS Isotherm Plot
Cycle 1 Sorp
Cycle 1 Desorp
Cycle 2 Sorp
Cycle 2 Desorp
Change In Mass (%) - Ref
Target % P/Po
Br
N
O
Na+
OH
S
H
DVS - The Sorption Solution
© Surface Measurement Systems Ltd UK 1996-2012

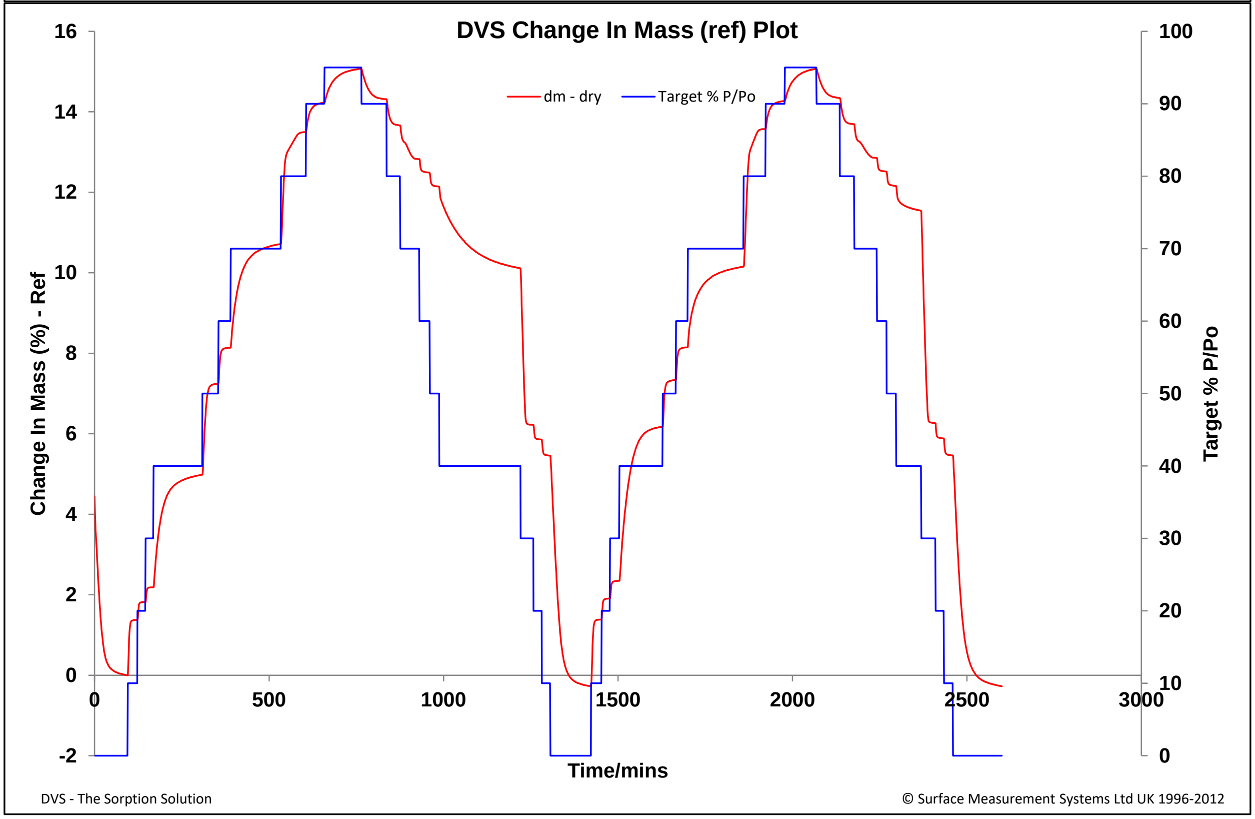

DVS Change In Mass (ref) Plot
dm - dry
Target % P/Po
Change In Mass (%) - Ref
Time/mins
DVS - The Sorption Solution
© Surface Measurement Systems Ltd UK 1996-2012

R_P-8-Bromo-β-phenyl-1,N^2-ethenoguanosine-3′,5′-cyclicmonophosphorothiotic acid (cGMPSA-H)

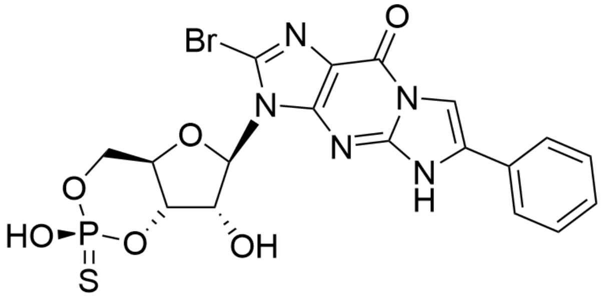

cGMPSA-TEA (1000 mg, 1.56 mmol) was stirred in deionized H_2O (30 mL, 30 volumes) at reflux until all or most of the starting material was dissolved. 2 M $HCl_{(aq)}$ (0.95 equiv., 740 µL, 1.5 mmol) was added dropwise into the vessel, which shortly caused a white slurry to form. After complete addition, the mixture was stirred for 1 hour before acclimating to RT and stirred for 1 further hour at RT. The white solid was filtered and washed with deionized H_2O (2 x 10 mL), followed by drying at 50°C under a vacuum. Yield: 80% (740 mg, 1.25 mmol). HPLC purity: 99.2%. ^{1}H NMR (500 MHz, DMSO-d_6): δ 13.28 (br s, 1H), 8.25 (s, 1H), 7.96–7.90 (m, 2H), 7.53–7.45 (m, 2H), 7.44–7.38 (m, 1H), 5.81 (d, J = 1.4 Hz, 1H), 5.17–5.14 (m, 1H), 5.01–4.95 (m, 1H), 4.55–4.45 (m, 1H), 4.32–4.25 (m, 1H), 4.20–4.11 (m, 1H). ^{13}C NMR (126 MHz, DMSO-d_6): δ 150.4, 150.0, 145.9, 129.6, 129.0, 128.9, 127.7, 125.3, 122.5, 116.0, 103.7, 93.2, 76.8 (d, J_{PC} = 7.4 Hz, 1C), 70.3 (d, J_{PC} = 5.7 Hz, 1C), 69.2 (d, J_{PC} = 7.4 Hz, 1C), 68.4 (d, J_{PC} = 11.0 Hz, 1C). MS (M – H^+) m/z: 537.96 calcd for $C_{18}H_{14}BrN_5O_6PS^-$; found, 537.95 (ES$^-$). DSC (exotherm, onset): 162.7 °C.

HPLC-UV-MS

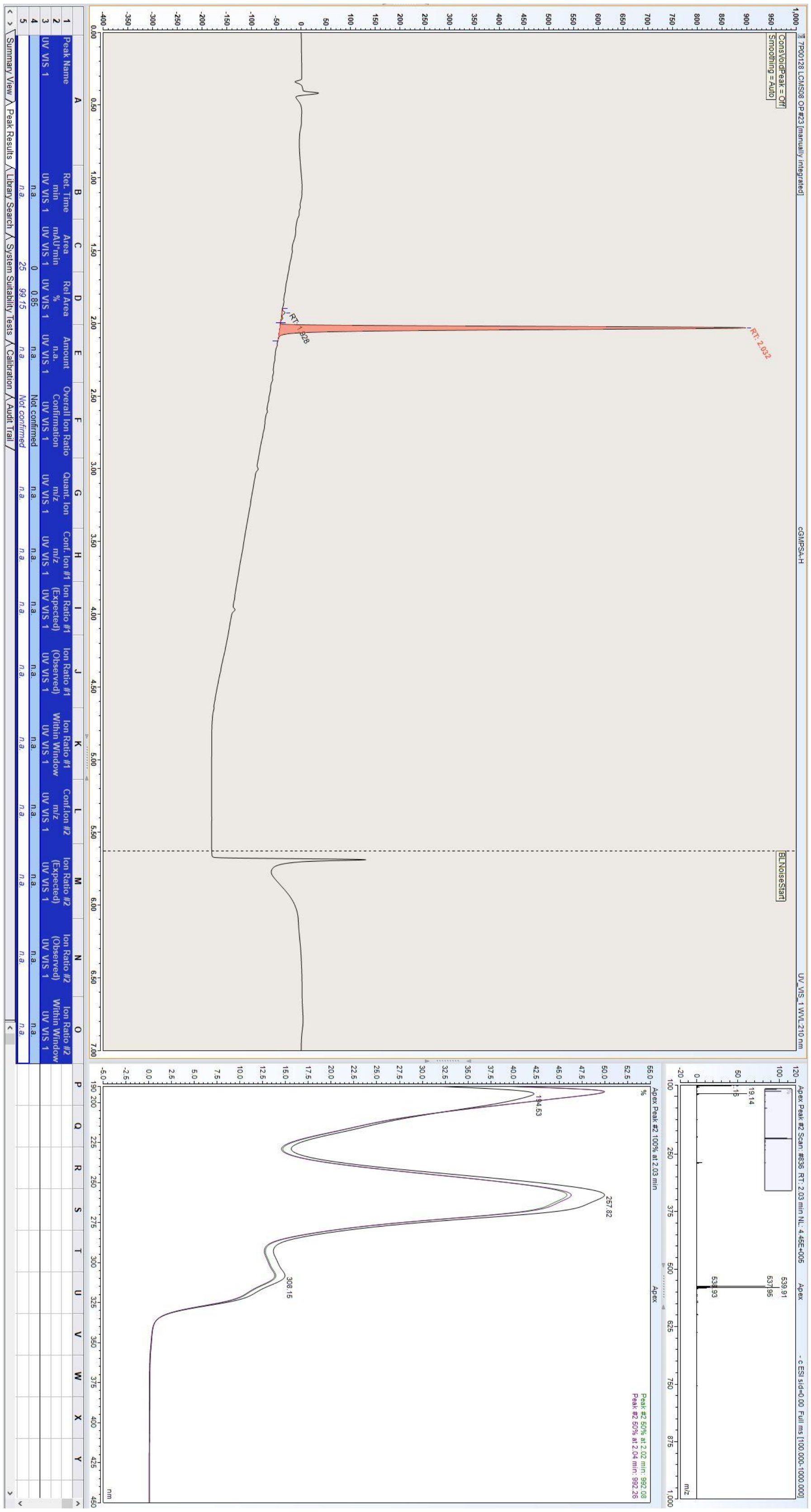

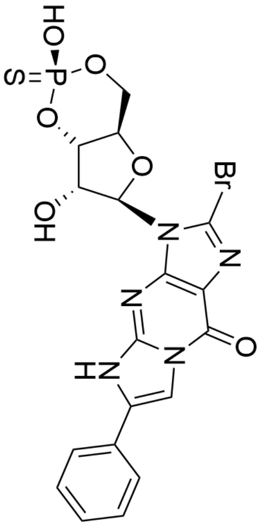

NMR

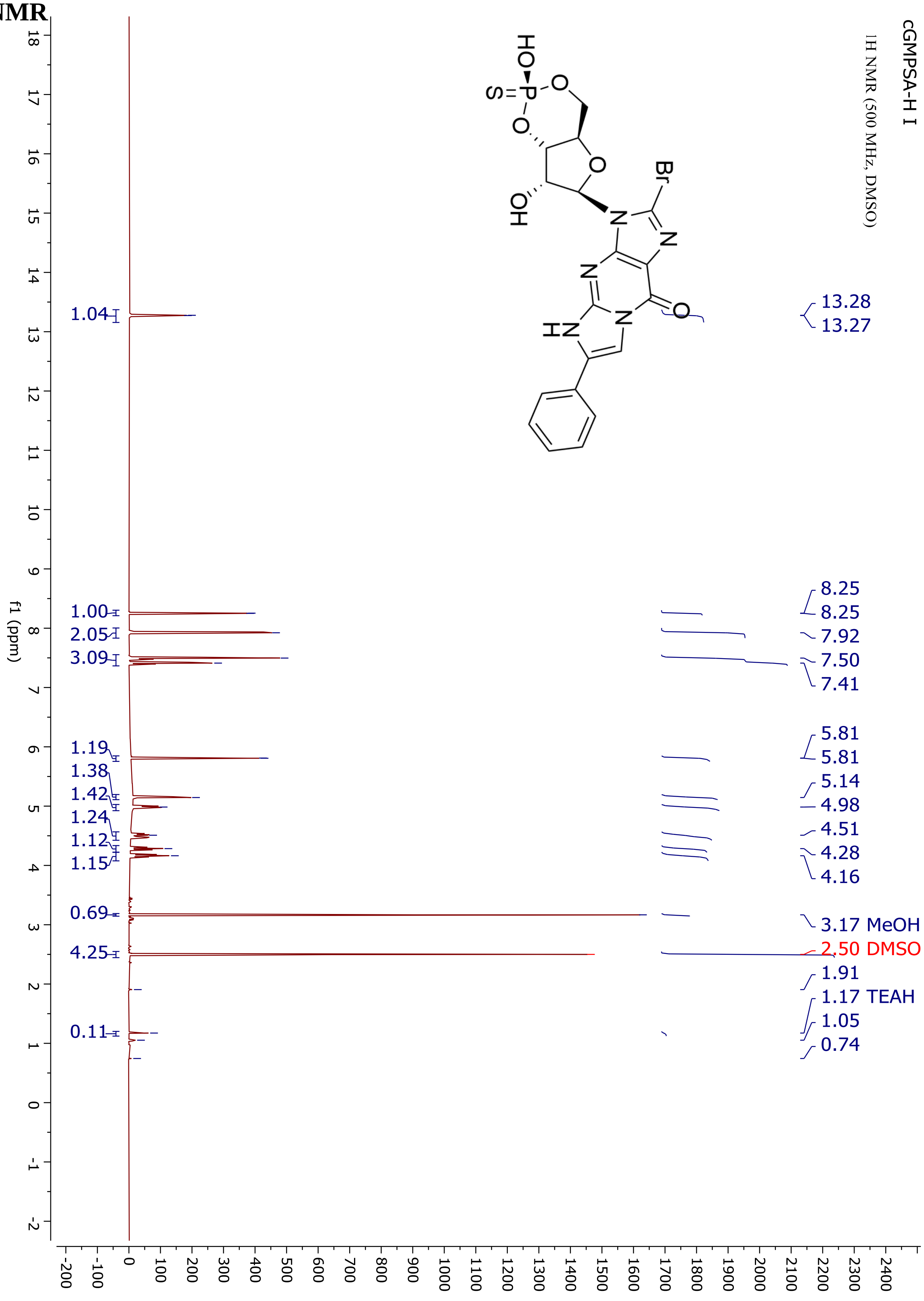
cGMPSA-H I
1H NMR (500 MHz, DMSO)
13.28
13.27
8.25
8.25
7.92
7.50
7.41
5.81
5.81
5.14
4.98
4.51
4.28
4.16
3.17 MeOH
2.50 DMSO
1.91
1.17 TEAH
1.05
0.74
1.04
1.00
2.05
3.09
1.19
1.38
1.42
1.24
1.12
1.15
0.69
4.25
0.11
f1 (ppm)

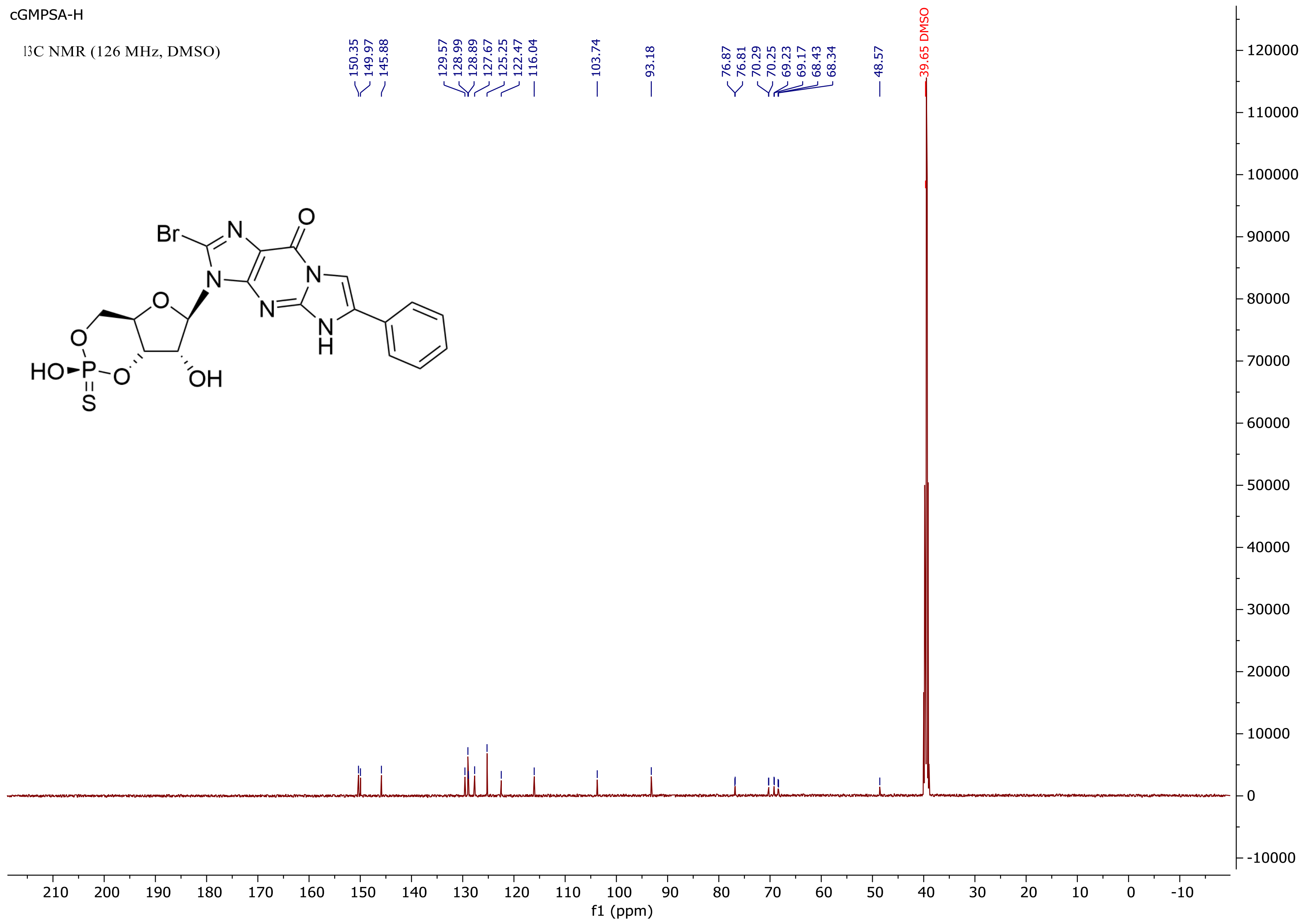
cGMPSA-H
13C NMR (126 MHz, DMSO)
150.35
149.97
145.88
129.57
128.99
128.89
127.67
125.25
122.47
116.04
103.74
93.18
76.87
76.81
70.29
70.25
69.23
69.17
68.43
68.34
48.57
39.65 DMSO
f1 (ppm)

XRPD

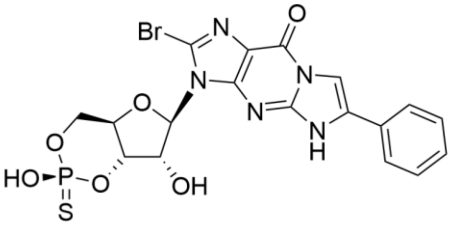

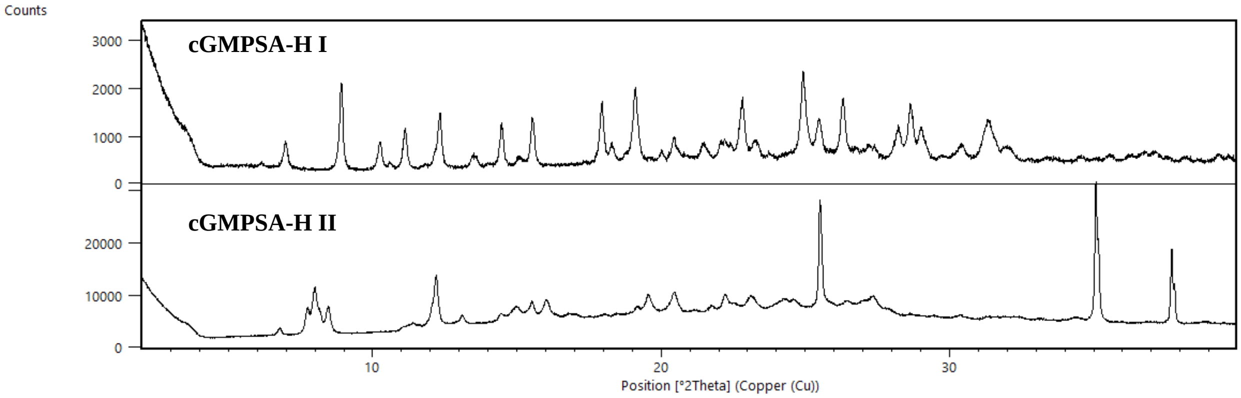
Counts
cGMPSA-H I
cGMPSA-H II
Position [°2Theta] (Copper (Cu))

DSC & TGA

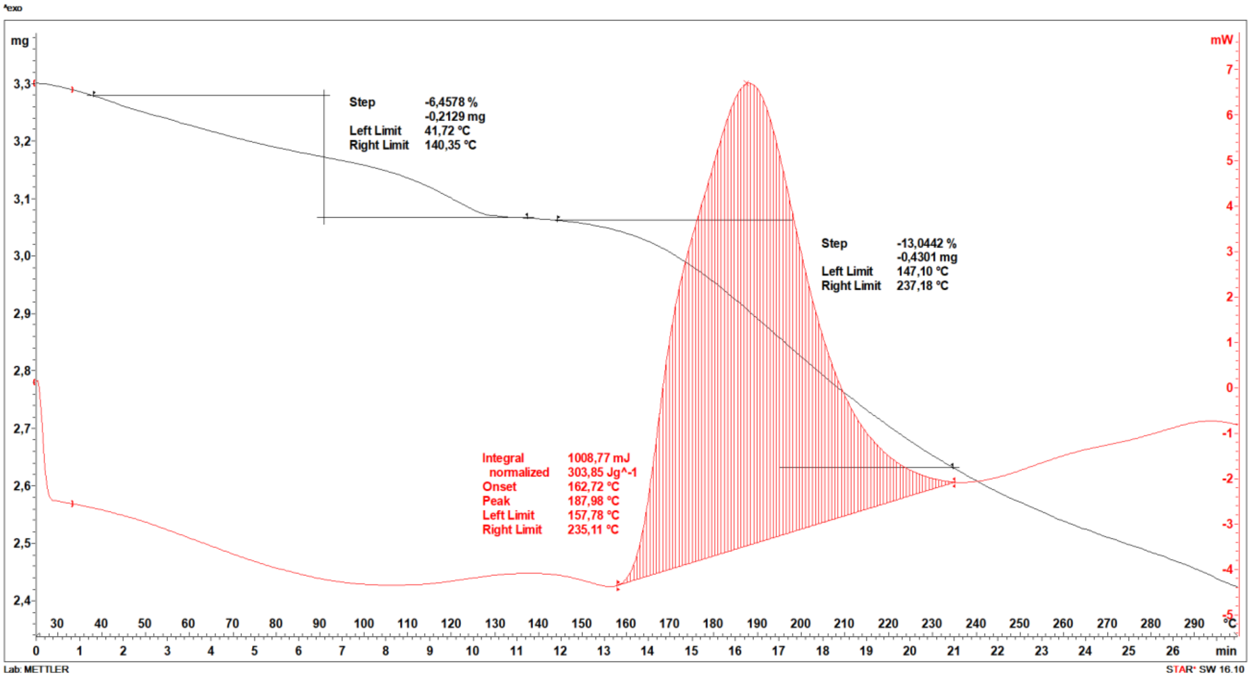
Step -6,4578 % -0,2129 mg
Left Limit 41,72 °C
Right Limit 140,35 °C
Step -13,0442 % -0,4301 mg
Left Limit 147,10 °C
Right Limit 237,18 °C
Integral 1008,77 mJ
normalized 303,85 Jg^-1
Onset 162,72 °C
Peak 187,98 °C
Left Limit 157,78 °C
Right Limit 235,11 °C
Lab: METTLER
STARe SW 16.10

DVS

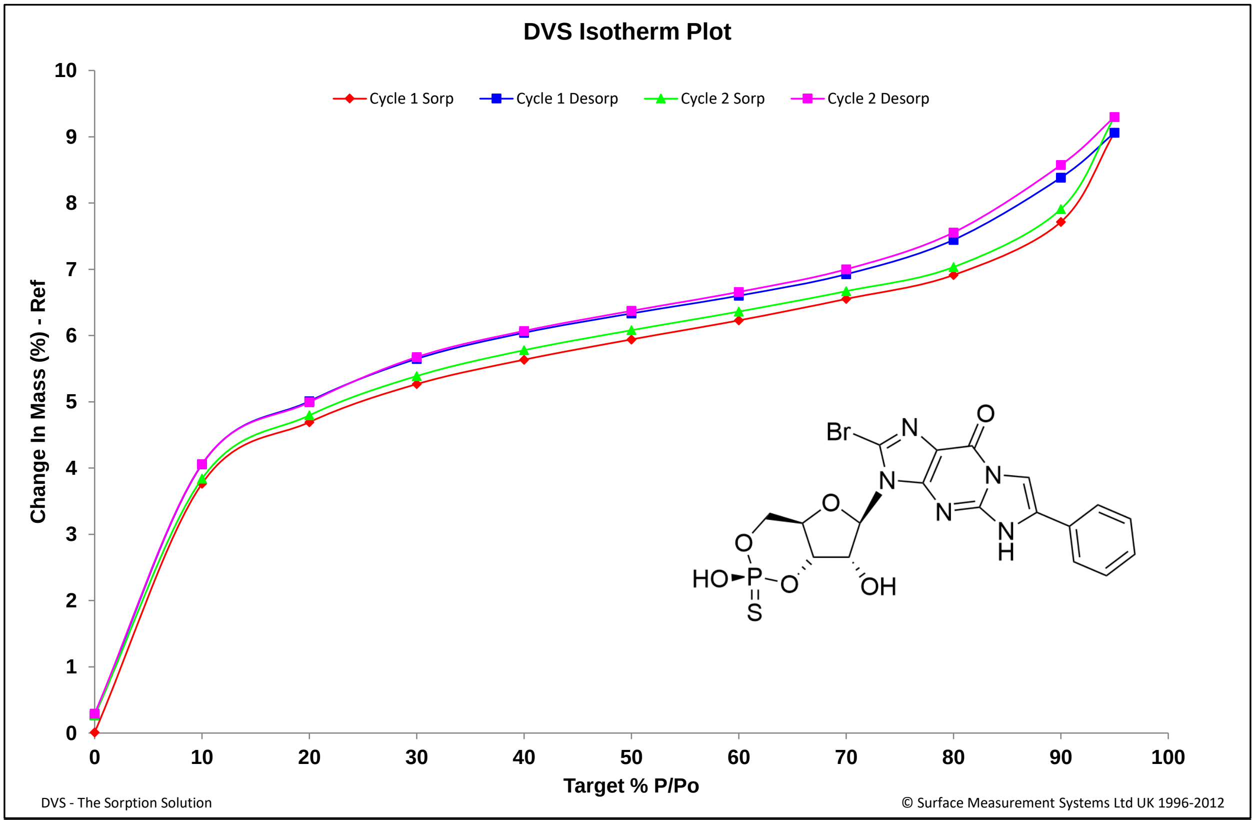
DVS Isotherm Plot
Cycle 1 Sorp
Cycle 1 Desorp
Cycle 2 Sorp
Cycle 2 Desorp
Change In Mass (%) - Ref
Target % P/Po
Br
N
O
HO
P
S
OH
H
DVS - The Sorption Solution
© Surface Measurement Systems Ltd UK 1996-2012

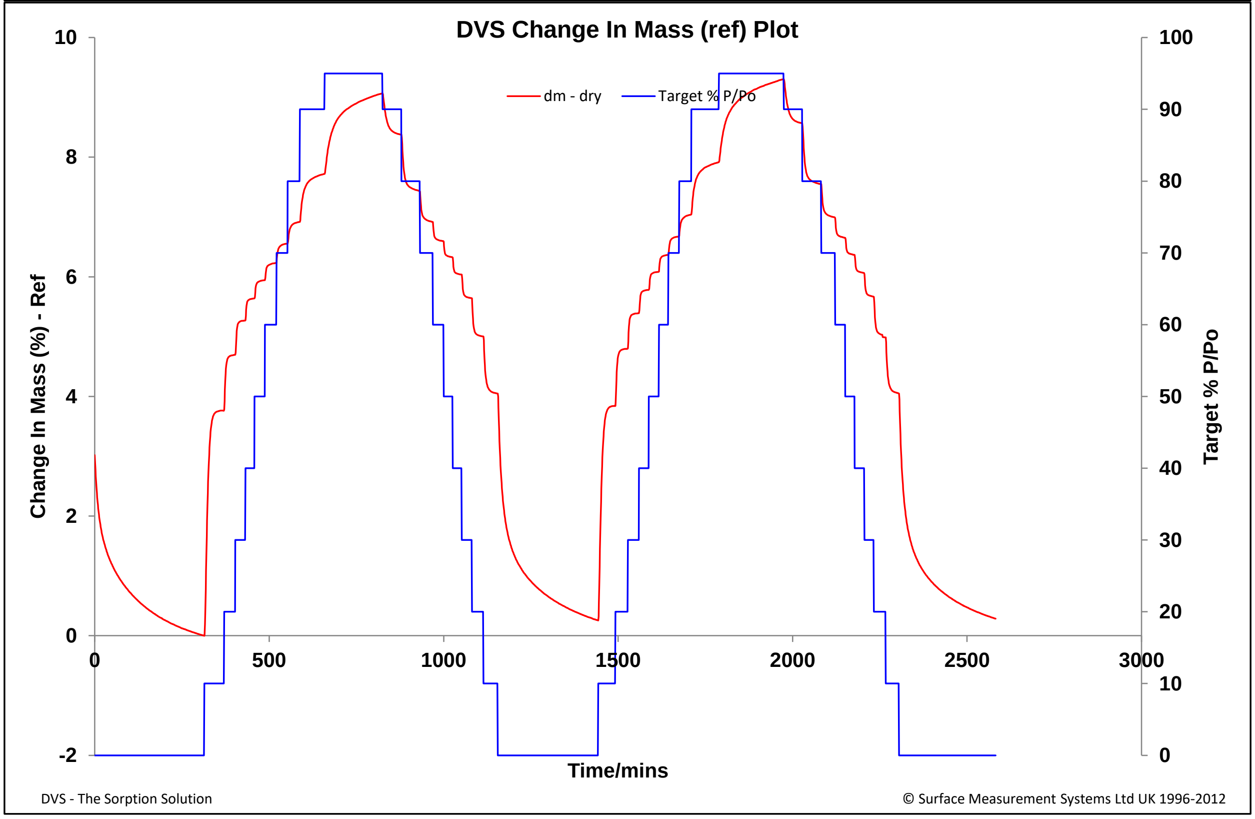
DVS Change In Mass (ref) Plot
dm - dry
Target % P/Po
Change In Mass (%) - Ref
Target % P/Po
Time/mins
DVS - The Sorption Solution
© Surface Measurement Systems Ltd UK 1996-2012

Calcium R_P-8-Bromo-β-phenyl-1,N^2-ethenoguanosine-3′,5′-cyclicmonophosphorothioate (1:2) (cGMPSA-Ca)

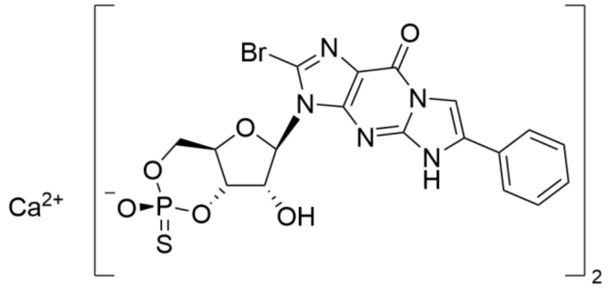

cGMPSA-TEA (15.5 g, 24.16 mmol) was stirred in refluxing MeOH (310 mL, 20 volumes) in a jacketed reactor. $CaCl_2$•$6H_2O$ (0.55 equiv., 1.48 g, 13.29 mmol) was dissolved in MeOH (53 mL, 15 volumes) and injected into the reactor over 2 h. The reactor bath temperature was slowly decreased to RT over 54 h. The white solid was filtered and washed with MeOH (2 x 15 mL). ^{1}H-NMR analysis of the product revealed some residual starting triethylamine. The product was subjected to the same procedure once more, and ^{1}H-NMR of the resulting solid showed only traces left of the triethylammonium starting material. The product was then dried at 50°C under a vacuum. Yield: 81% (11 g, 9.83 mmol). HPLC purity: >98.8%. ^{1}H NMR (500 MHz, DMSO-d_6): δ 13.27 (br s, 1H), 8.25 (s, 1H), 7.95–7.89 (m, 2H), 7.52–7.46 (m, 2H), 7.43–7.38 (m, 1H), 5.90 (d, J = 4.9 Hz, 1H), 5.77 (d, J = 1.4 Hz, 1H), 5.04–4.99 (m, 1H), 4.99–4.93 (m, 1H), 4.24–4.10 (m, 2H), 4.05–3.97 (m, 1H). ^{13}C NMR (126 MHz, DMScO-d_6): δ 150.4, 150.0, 145.8, 129.5, 129.0, 128.9, 127.6, 125.2, 122.3, 116.0, 103.6, 93.2, 75.2 (d, J_{PC} = 6.4 Hz, 1C), 71.6 (d, J_{PC} = 5.3 Hz, 1C), 70.1 (d, J_{PC} = 7.2 Hz, 1C), 65.8 (d, J_{PC} = 9.4 Hz, 1C). MS (M + H^+) m/z: 539.97 calcd for $C_{18}H_{16}BrN_5O_6PS^+$; found, 539.95 (ES^+). DSC (exotherm, onset): 252.5 °C.

HPLC-UV-MS

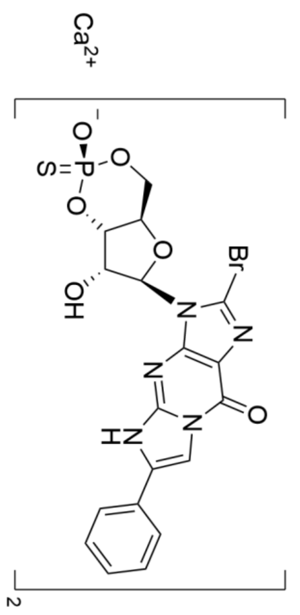

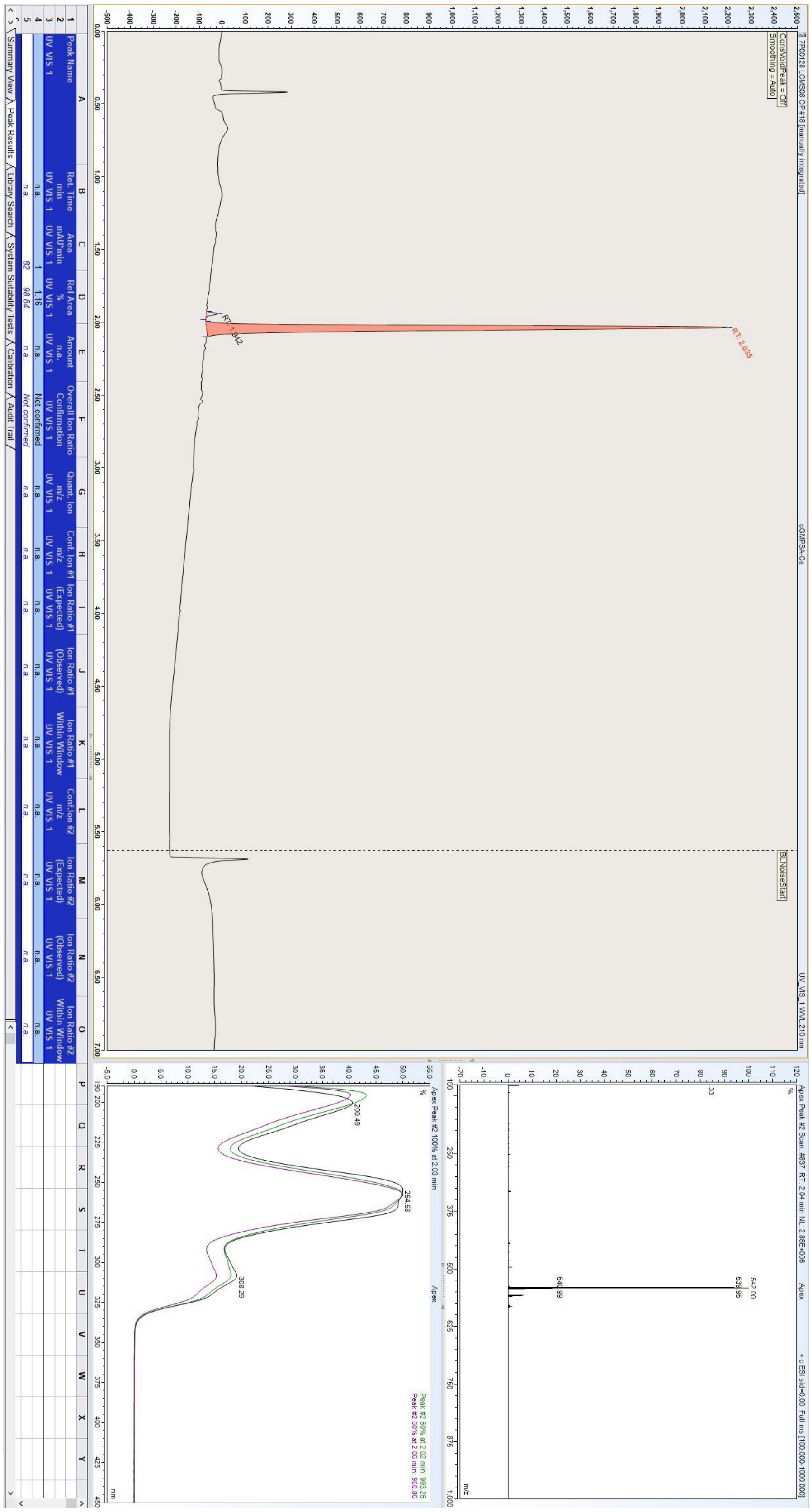

NMR

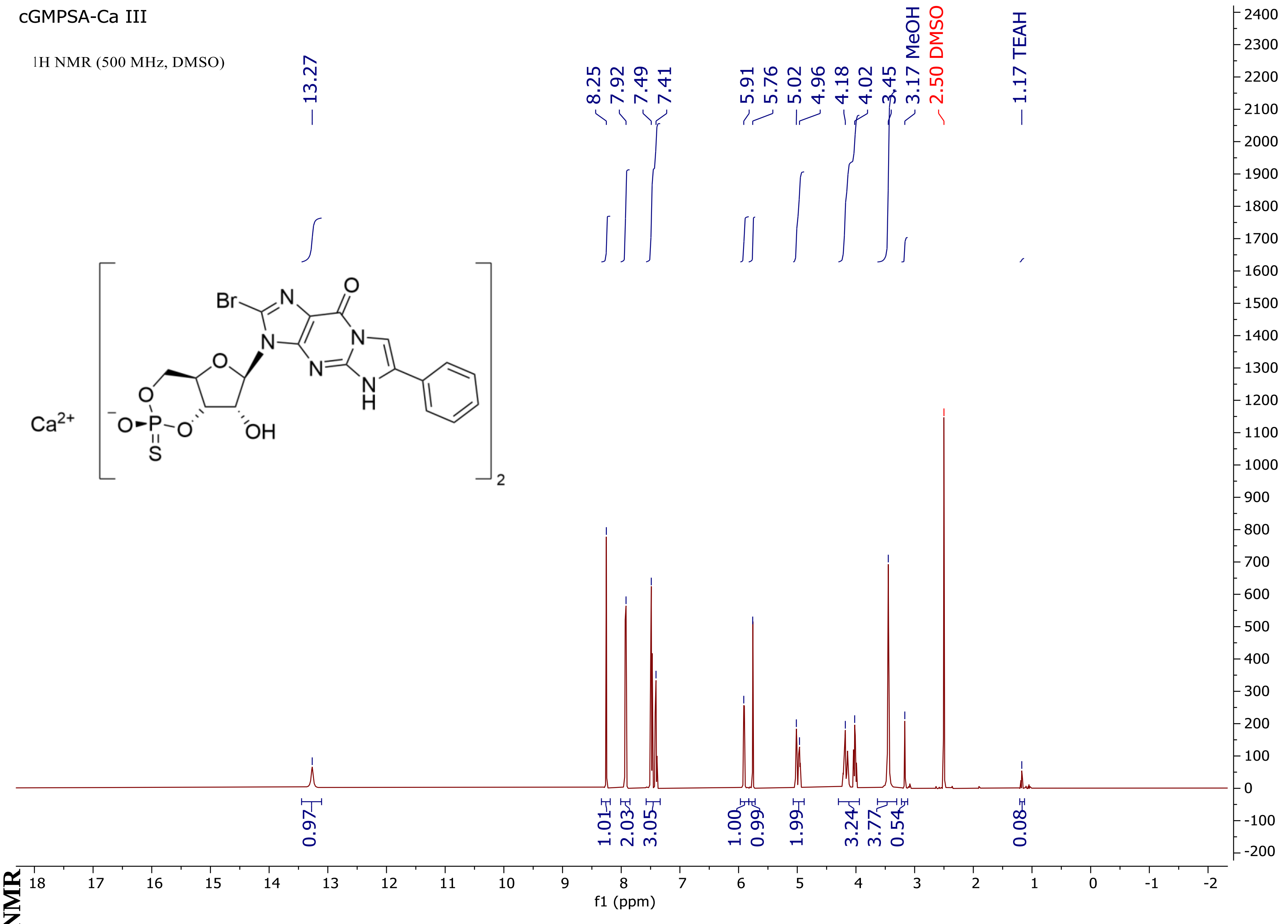

cGMPSA-Ca III
1H NMR (500 MHz, DMSO)
13.27
8.25
7.92
7.49
7.41
5.91
5.76
5.02
4.96
4.18
4.02
3.45
3.17 MeOH
2.50 DMSO
1.17 TEAH
0.97
1.01
2.03
3.05
1.00
0.99
1.99
3.24
3.77
0.54
0.08
Ca2+
f1 (ppm)

cGMPSA-Ca

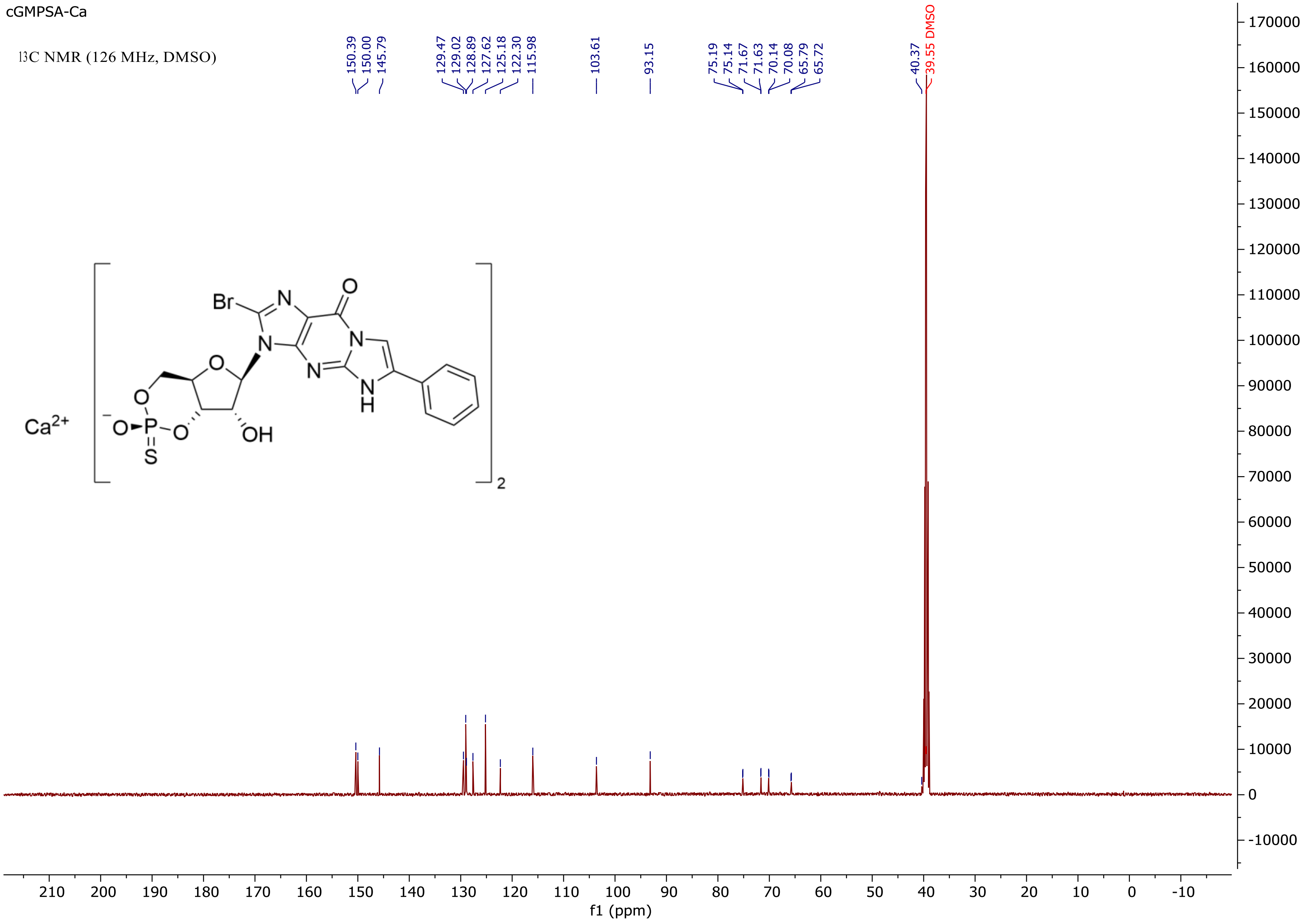

13C NMR (126 MHz, DMSO)
150.39
150.00
145.79
129.47
129.02
128.89
127.62
125.18
122.30
115.98
103.61
93.15
75.19
75.14
71.67
71.63
70.14
70.08
65.79
65.72
40.37
39.55 DMSO
Br
N
O
OH
H
S
P
Ca2+
2
f1 (ppm)

XRPD

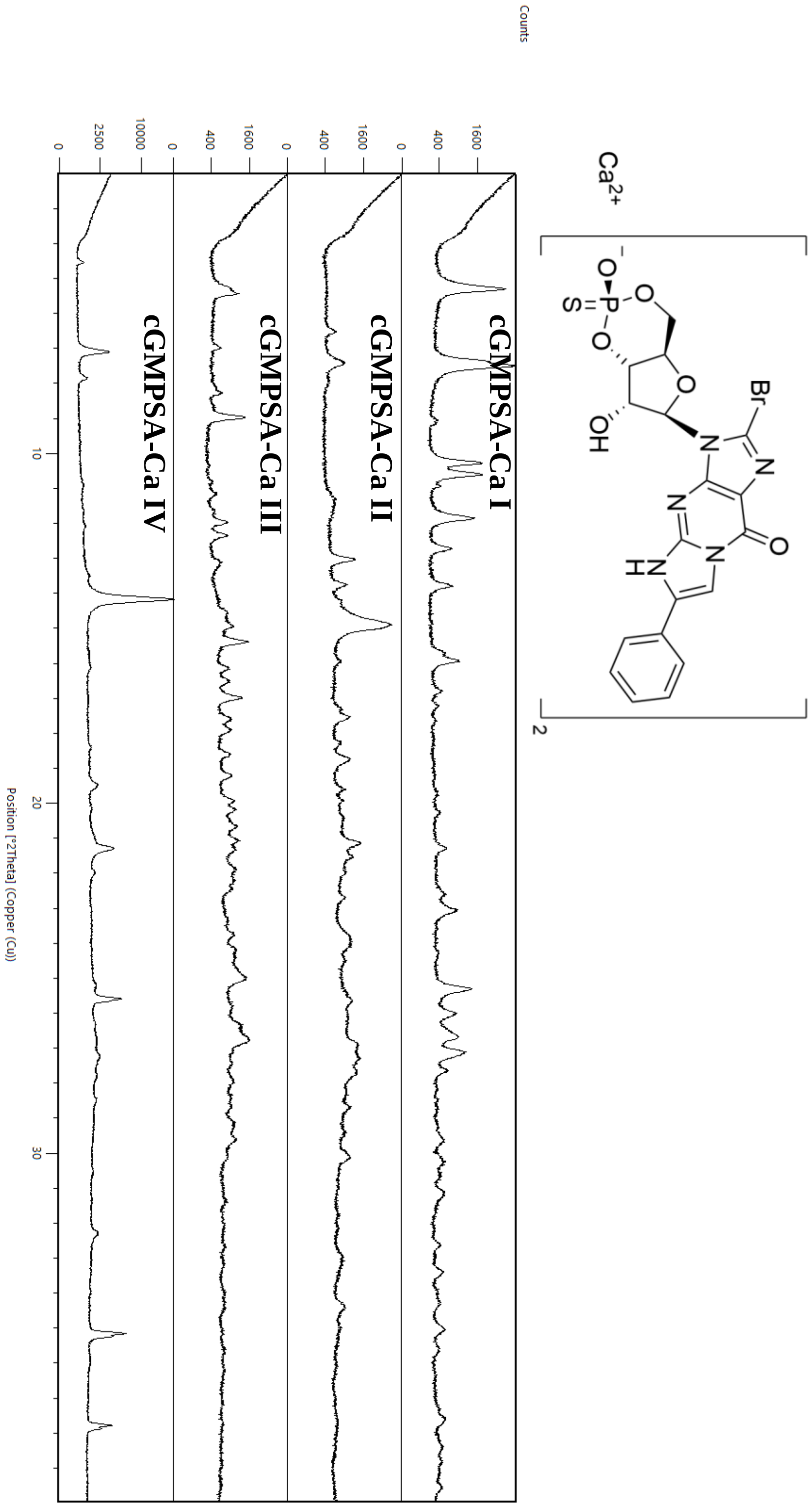
Counts
cGMPSA-Ca I
cGMPSA-Ca II
cGMPSA-Ca III
cGMPSA-Ca IV
Position [°2Theta] (Copper (Cu))
Ca2+
Br
OH
2

DSC & TGA

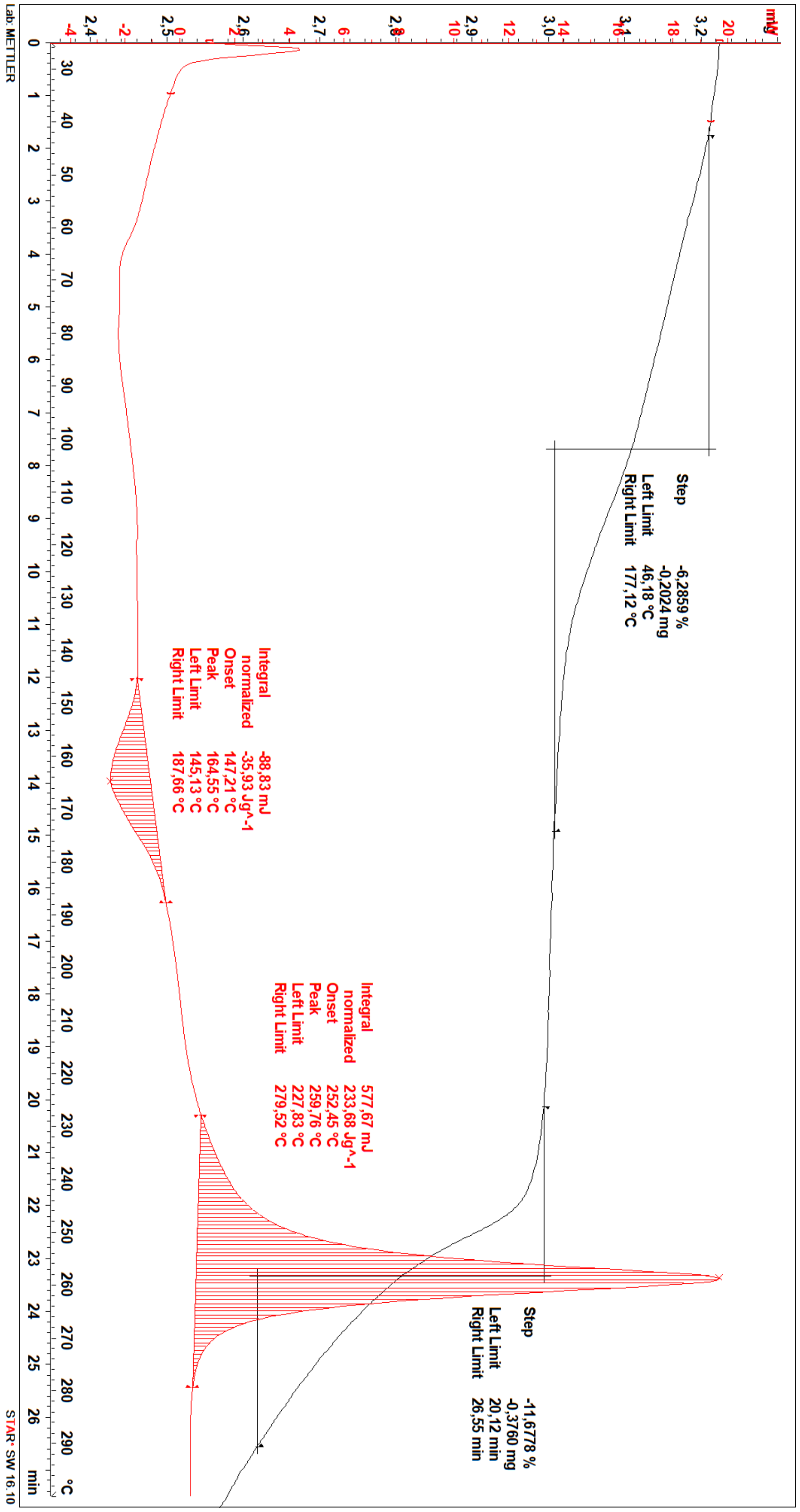
^exo
mg
mW
Step -6,2859 %
-0,2024 mg
Left Limit 46,18 °C
Right Limit 177,12 °C
Integral -88,83 mJ
normalized -35,93 Jg^-1
Onset 147,21 °C
Peak 164,55 °C
Left Limit 145,13 °C
Right Limit 187,66 °C
Integral 577,67 mJ
normalized 233,68 Jg^-1
Onset 252,45 °C
Peak 259,76 °C
Left Limit 227,83 °C
Right Limit 279,52 °C
Step -11,6778 %
-0,3760 mg
Left Limit 20,12 min
Right Limit 26,55 min
min
°C
Lab: METTLER
STARe SW 16.10

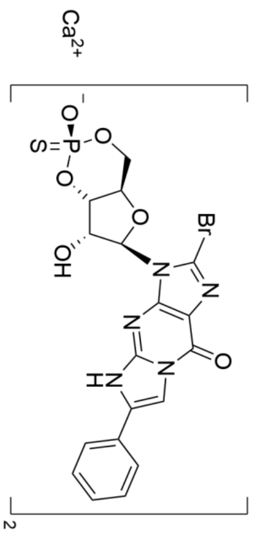

DVS

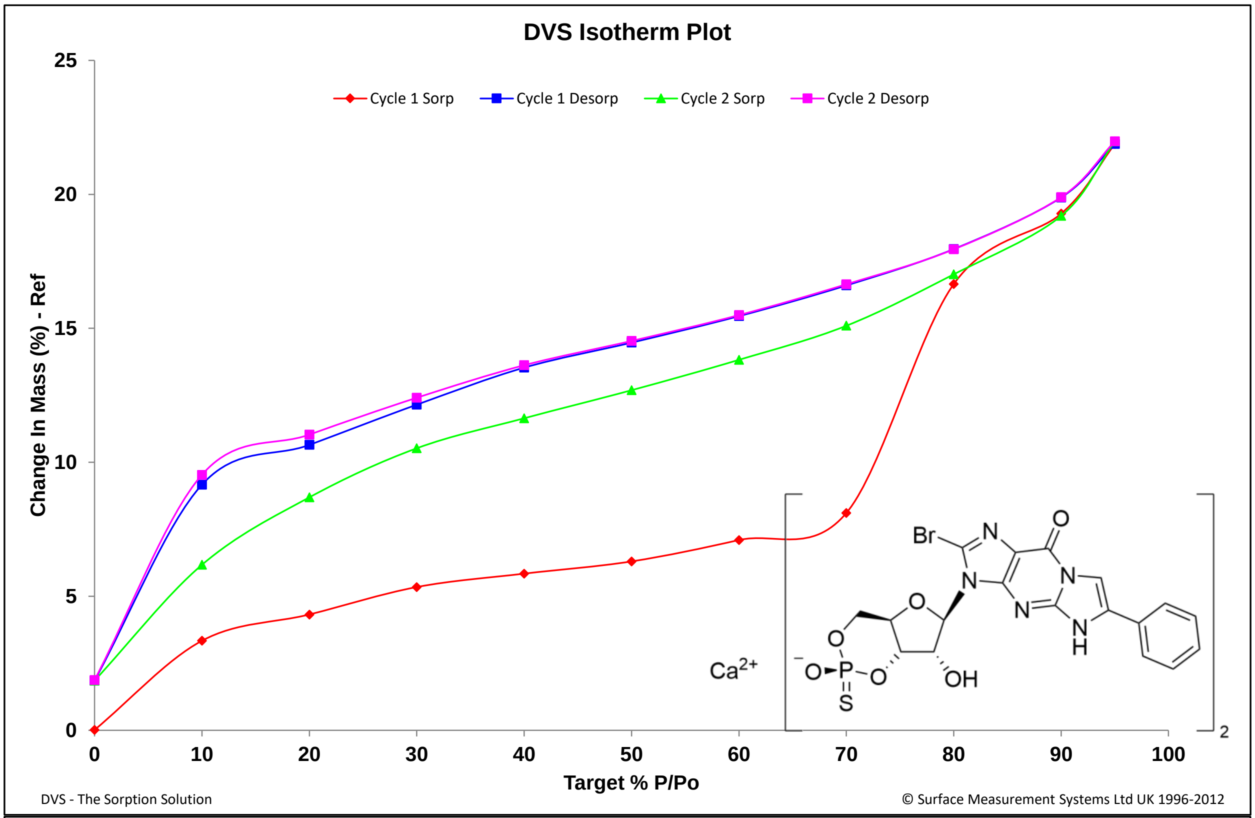

DVS Isotherm Plot
Cycle 1 Sorp
Cycle 1 Desorp
Cycle 2 Sorp
Cycle 2 Desorp
Change In Mass (%) - Ref
Target % P/Po
0
5
10
15
20
25
0
10
20
30
40
50
60
70
80
90
100
Ca2+
Br
N
O
OH
P
S
H
2
DVS - The Sorption Solution
© Surface Measurement Systems Ltd UK 1996-2012

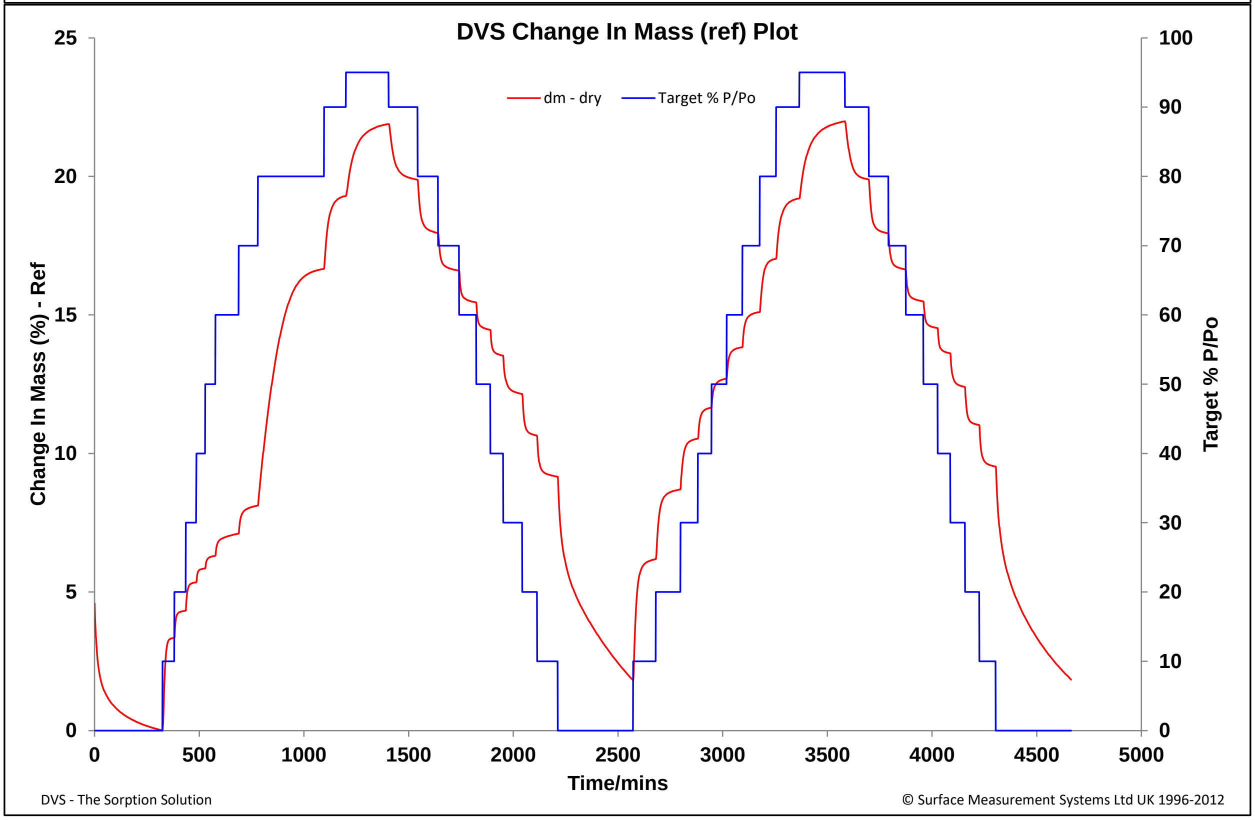

DVS Change In Mass (ref) Plot
dm - dry
Target % P/Po
Change In Mass (%) - Ref
Target % P/Po
Time/mins
0
500
1000
1500
2000
2500
3000
3500
4000
4500
5000
0
5
10
15
20
25
0
10
20
30
40
50
60
70
80
90
100
DVS - The Sorption Solution
© Surface Measurement Systems Ltd UK 1996-2012

Ammonium R_P-8-Bromo-β-phenyl-1,N^2-ethenoguanosine-3′,5′-cyclicmonophosphorothioate (cGMPSA-NH_4)

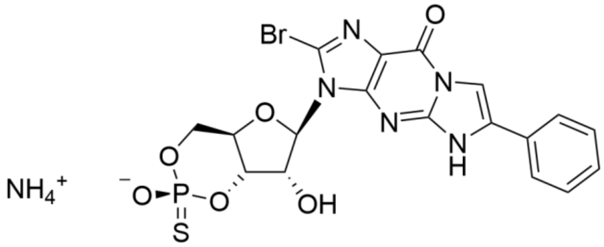

cGMPSA-TEA (200 mg, 3.12 mmol) was suspended in an excess of 30% $NH_4OH_{(aq)}$ and MeOH (1:1 v/v) in a round bottom flask. The mixture was evaporated in a rotary evaporator under reduced pressure. The residue was then suspended in an excess of 30% w/w $NH_4OH_{(aq)}$ and 2-PrOH (2:1 v/v) and evaporated once more, and the residue analysed by ^{1}H-NMR in DMSO-d_6. This was repeated until no triethylamine was observable by ^{1}H-NMR. Yield not available due to material losses to NMR and XRPD analyses. HPLC purity: 90.6%. ^{1}H NMR (500 MHz, DMSO-d_6): δ 13.63 (br s, 1H), 8.23 (s, 1H), 7.96–7.91 (m, 2H), 7.52–7.46 (m, 2H), 7.43–7.38 (m, 1H), 7.13 (br t, J_{NH} = 44.1 Hz, 4H), 5.85 (d, J = 4.9 Hz, 1H), 5.72 (d, J = 1.6 Hz, 1H), 5.10–5.05 (m, 1H), 4.93–4.88 (m, 1H), 4.17–4.05 (m, 2H), 4.03–3.96 (m, 1H). ^{13}C NMR (126 MHz, DMScO-d_6): δ 150.4, 150.0, 145.9, 129.6, 129.0, 128.8, 127.8, 125.2, 122.7, 116.0, 103.6, 93.4, 75.3 (d, J_{PC} = 6.2 Hz, 1C), 71.5 (d, J_{PC} = 5.5 Hz, 1C), 69.6 (d, J_{PC} = 6.9 Hz, 1C), 65.6 (d, J_{PC} = 9.2 Hz, 1C). MS (M – NH_4^+) m/z: 537.96 calcd for $C_{18}H_{14}BrN_5O_6PS^-$; found, 537.97 (ES$^-$). DSC (exotherm, onset): 177.4 °C.

HPLC-UV-MS

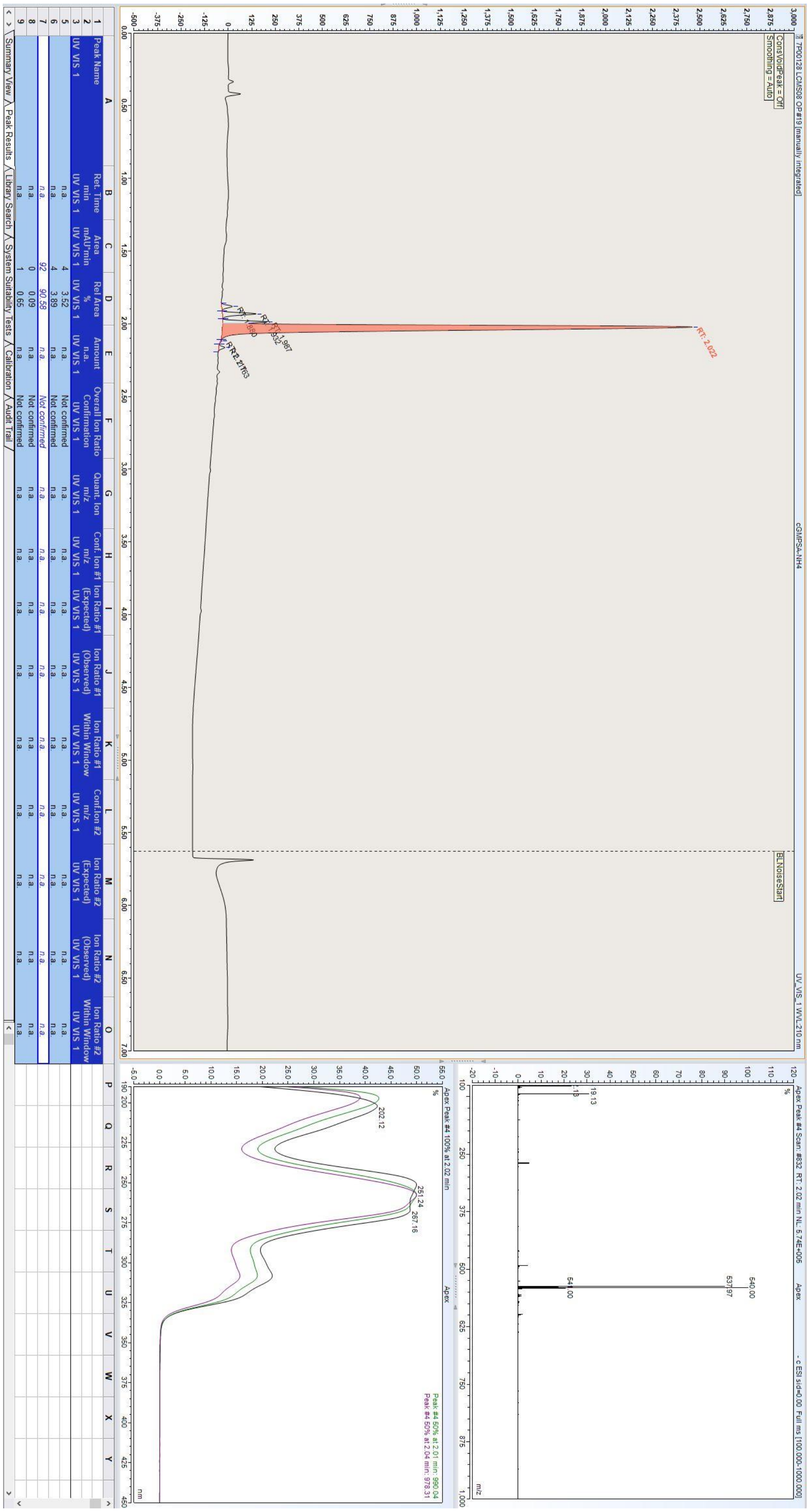

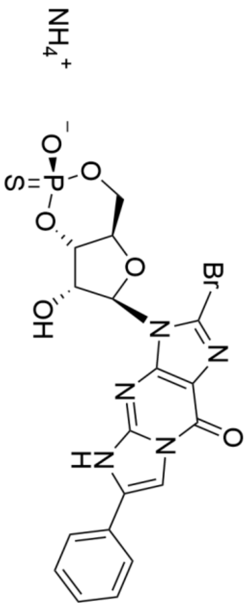

NMR

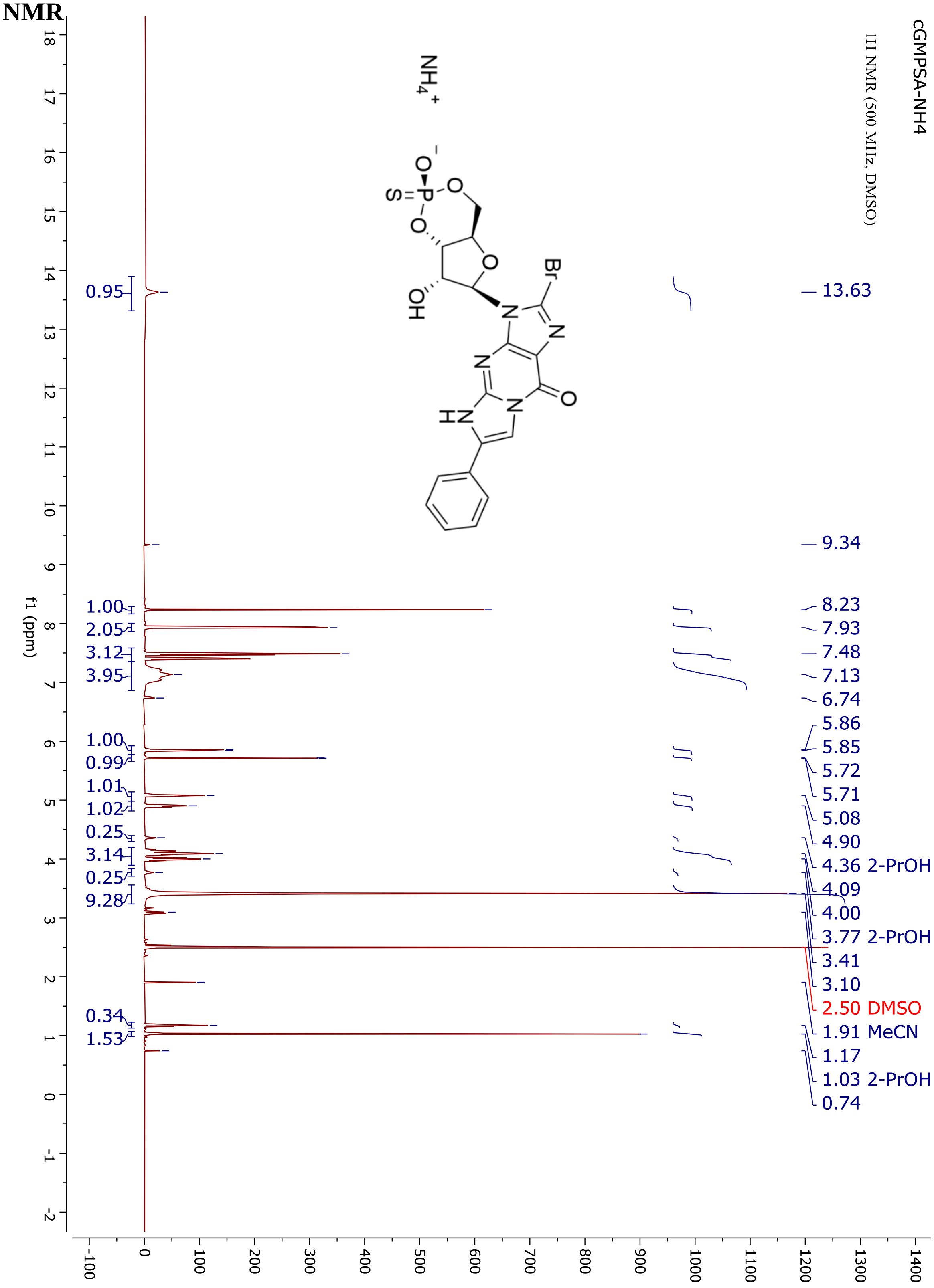
cGMPSA-NH4
1H NMR (500 MHz, DMSO)
13.63
9.34
8.23
7.93
7.48
7.13
6.74
5.86
5.85
5.72
5.71
5.08
4.90
4.36 2-PrOH
4.09
4.00
3.77 2-PrOH
3.41
3.10
2.50 DMSO
1.91 MeCN
1.17
1.03 2-PrOH
0.74
0.95
1.00
2.05
3.12
3.95
1.00
0.99
1.01
1.02
0.25
3.14
0.25
9.28
0.34
1.53
f1 (ppm)
Br
OH
NH₄⁺

cGMPSA-NH4

13C NMR (126 MHz, DMSO)

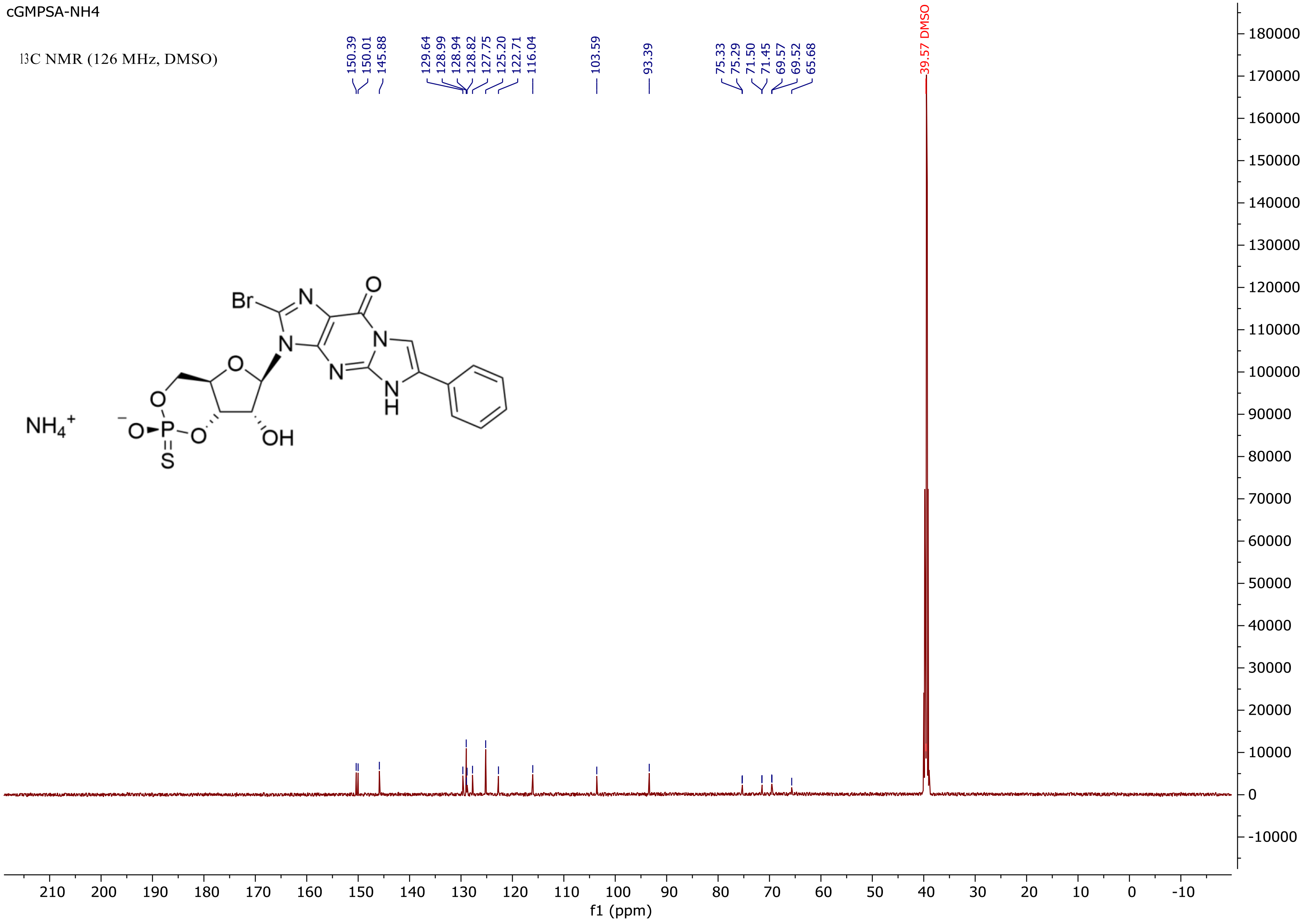

DSC & TGA

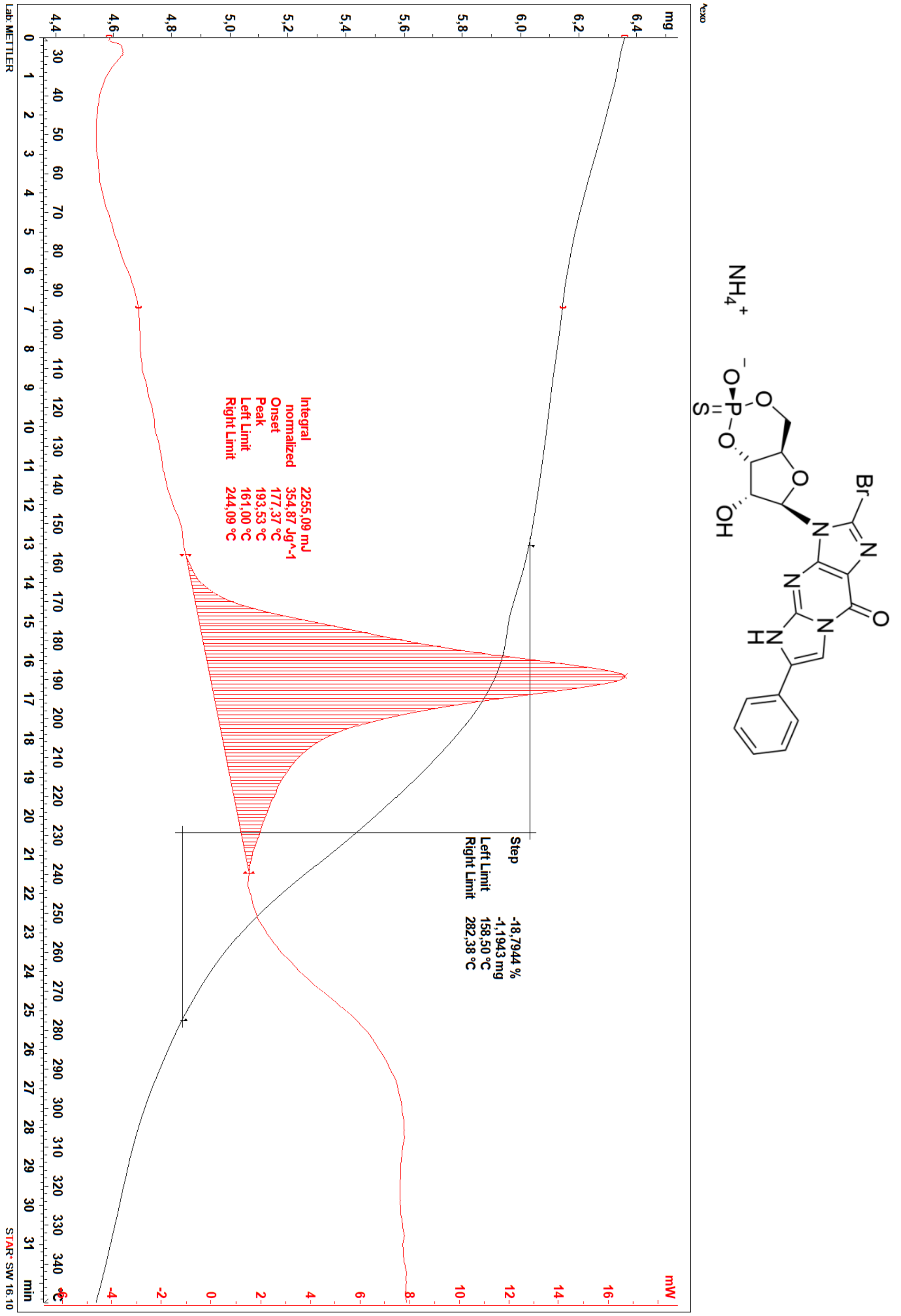
Integral 2255,09 mJ
normalized 354,87 Jg^-1
Onset 177,37 °C
Peak 193,53 °C
Left Limit 161,00 °C
Right Limit 244,09 °C
Step -18,7944 %
-1,1943 mg
Left Limit 158,50 °C
Right Limit 282,38 °C
mg
mW
min
°C
Lab: METTLER
STARe SW 16.10
NH4+
Br
OH

Tris(hydroxymethyl)aminomethane *R*$_P$-8-Bromo-β-phenyl-1,*N*^{2}-ethenoguanosine-3′,5′-cyclicmonophosphorothiotic acid (cGMPSA-Tris)

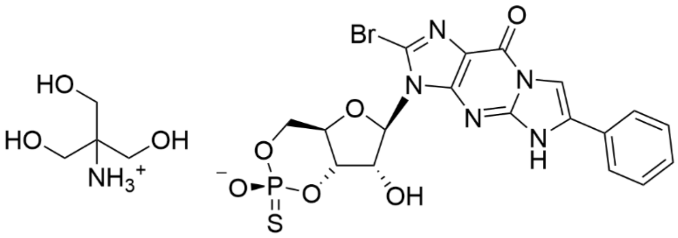

cGMPSA-TEA (200 mg, 0.31 mmol) and tris(hydroxymethyl)aminomethane (1.1 equiv., 41.6 mg, 0.34 mmol) were dissolved in a 1:1 H_2O:MeOH mixture in a round bottom flask. The mixture was concentrated in a rotary evaporator under reduced pressure. The residue was then suspended in an excess of 2-PrOH, evaporated once more, and analysed by ^{1}H-NMR. This was repeated until no triethylamine was observable by ^{1}H-NMR. Yield not available due to material losses to NMR and XRPD analyses. HPLC purity: >98.3%. ^{1}H NMR (500 MHz, DMSO-d_6): δ 8.13 (s, 1H), 7.95–7.89 (m, 2H), 7.48–7.41 (m, 2H), 7.38–7.32 (m, 1H), 5.83 (br s, 1H), 5.69 (d, J = 1.5 Hz, 1H), 5.12–5.06 (m, 1H), 4.99–4.92 (m, 1H), 4.17–4.07 (m, 2H), 4.02–3.95 (m, 1H), 3.49 (s, 6H). ^{13}C NMR (126 MHz, DMScO-d_6): δ 151.0, 150.8, 149.3, 132.7, 128.5, 129.0, 127.2, 125.1, 121.0, 114.1, 102.0, 93.4, 75.2 (d, J_{PC} = 6.4 Hz, 1C), 71.3 (d, J_{PC} = 5.3 Hz, 1C), 69.4 (d, J_{PC} = 7.4 Hz, 1C), 65.6 (d, J_{PC} = 9.1 Hz, 1C), 60.5, 59.7. MS (M – Tris$^+$) *m*/*z*: 537.96 calcd for $C_{18}H_{14}BrN_5O_6PS^-$; found, 537.98 (ES$^–$). DSC (exotherm, onset): 199.2 °C.

HPLC-UV-MS

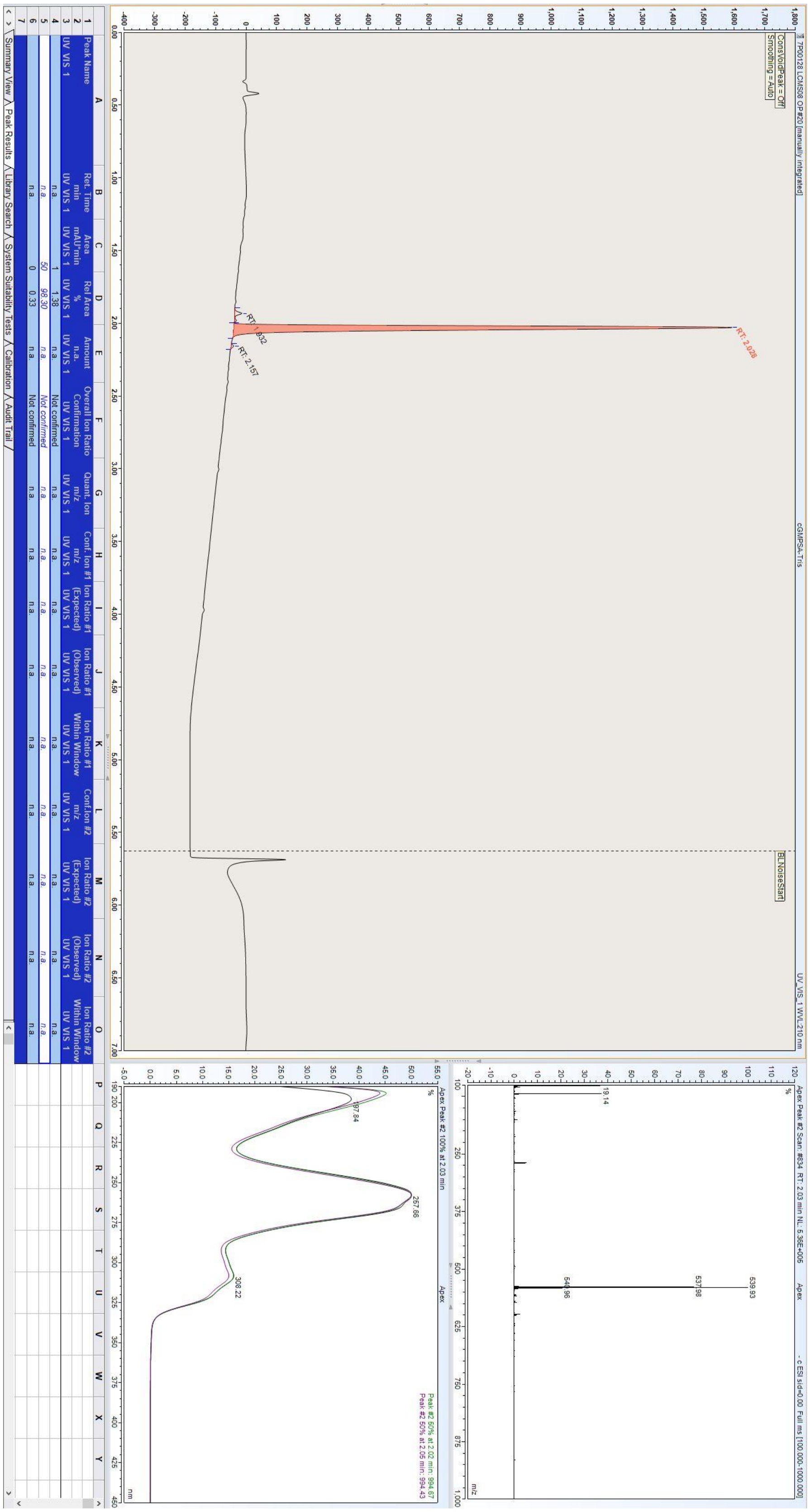

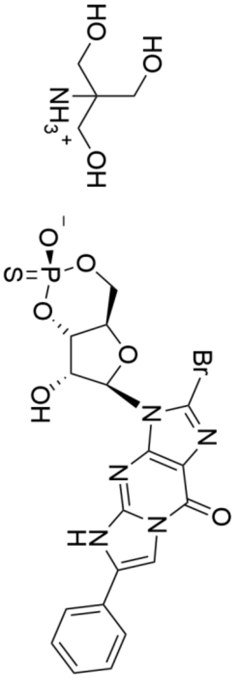

NMR

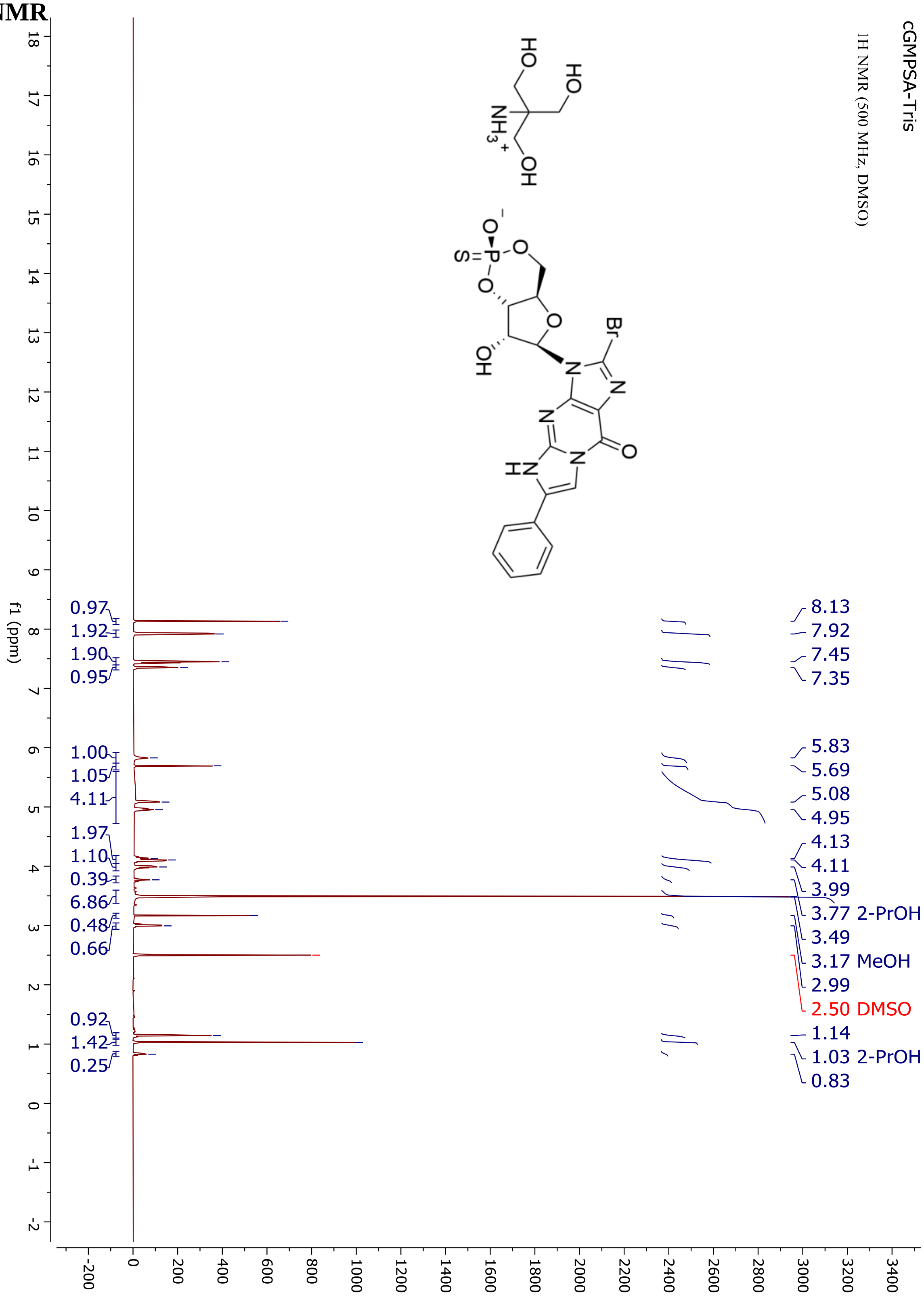

cGMPSA-Tris
1H NMR (500 MHz, DMSO)
8.13
7.92
7.45
7.35
5.83
5.69
5.08
4.95
4.13
4.11
3.99
3.77 2-PrOH
3.49
3.17 MeOH
2.99
2.50 DMSO
1.14
1.03 2-PrOH
0.83
0.97
1.92
1.90
0.95
1.00
1.05
4.11
1.97
1.10
0.39
6.86
0.48
0.66
0.92
1.42
0.25
f1 (ppm)

cGMPSA-Tris

13C NMR (126 MHz, DMSO)

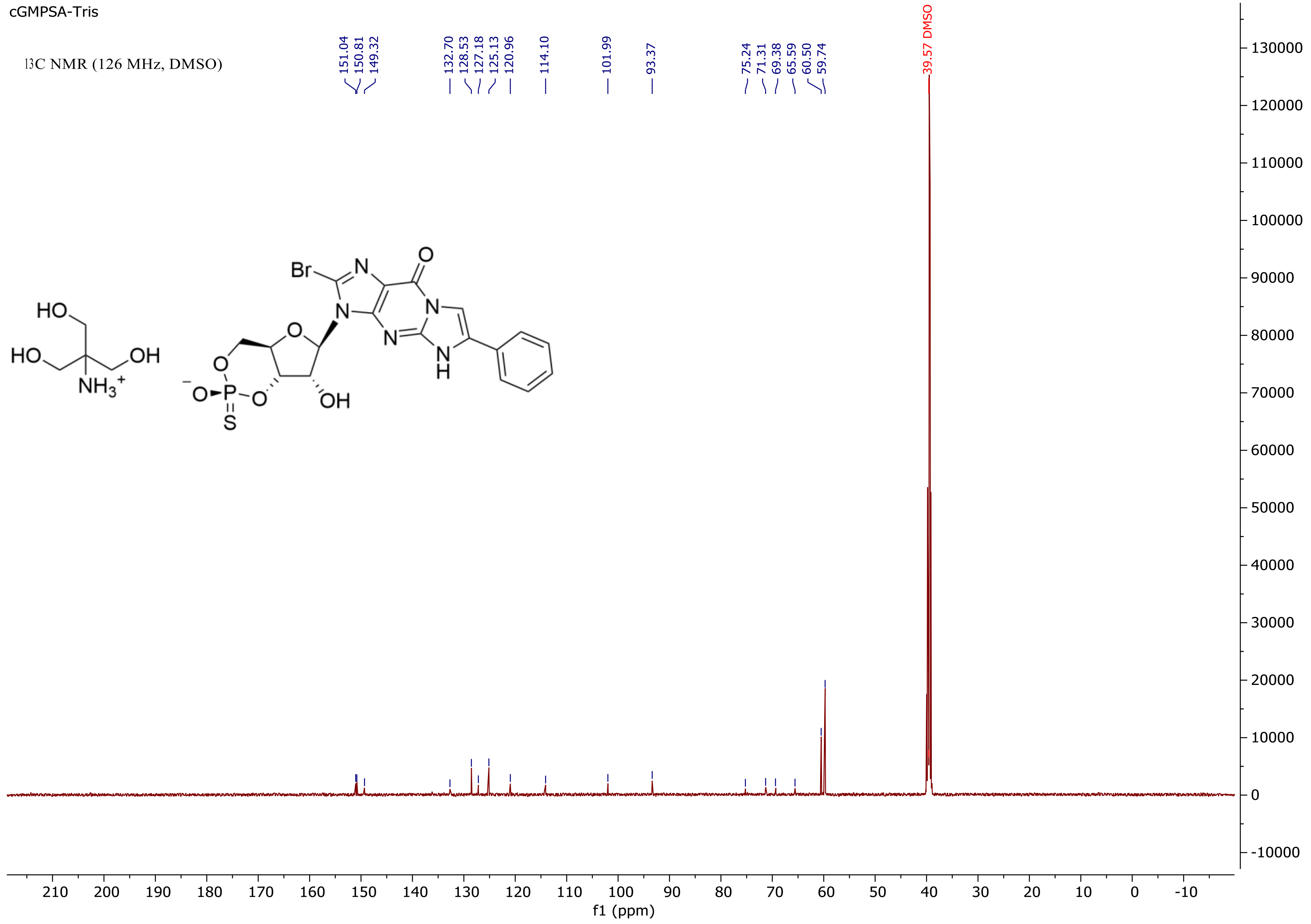

DSC & TGA

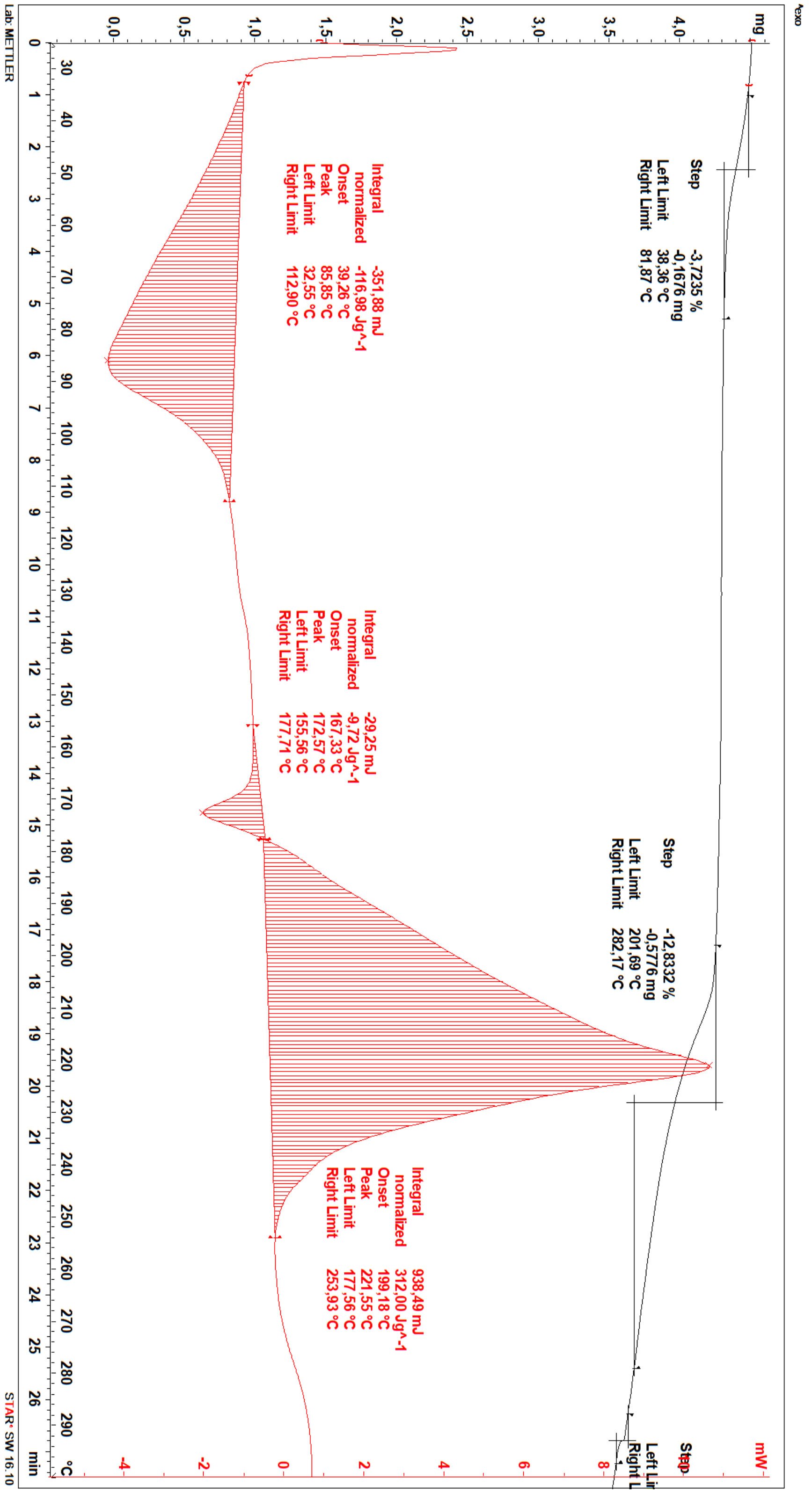
^exo
mg
mW
Step -3,7235 % -0,1676 mg
Left Limit 38,36 °C
Right Limit 81,87 °C
Integral -351,88 mJ
normalized -116,98 Jg^-1
Onset 39,26 °C
Peak 85,85 °C
Left Limit 32,55 °C
Right Limit 112,90 °C
Integral -29,25 mJ
normalized -9,72 Jg^-1
Onset 167,33 °C
Peak 172,57 °C
Left Limit 155,56 °C
Right Limit 177,71 °C
Step -12,8332 % -0,5776 mg
Left Limit 201,69 °C
Right Limit 282,17 °C
Integral 938,49 mJ
normalized 312,00 Jg^-1
Onset 199,18 °C
Peak 221,55 °C
Left Limit 177,56 °C
Right Limit 253,93 °C
Lab: METTLER
STARe SW 16.10
min
°C

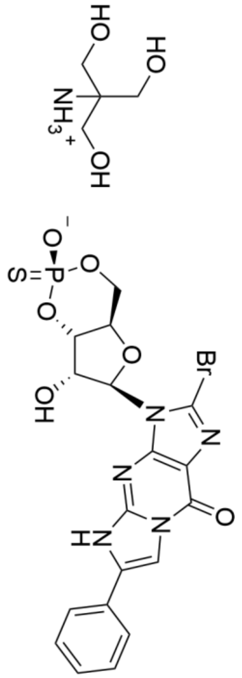

DVS

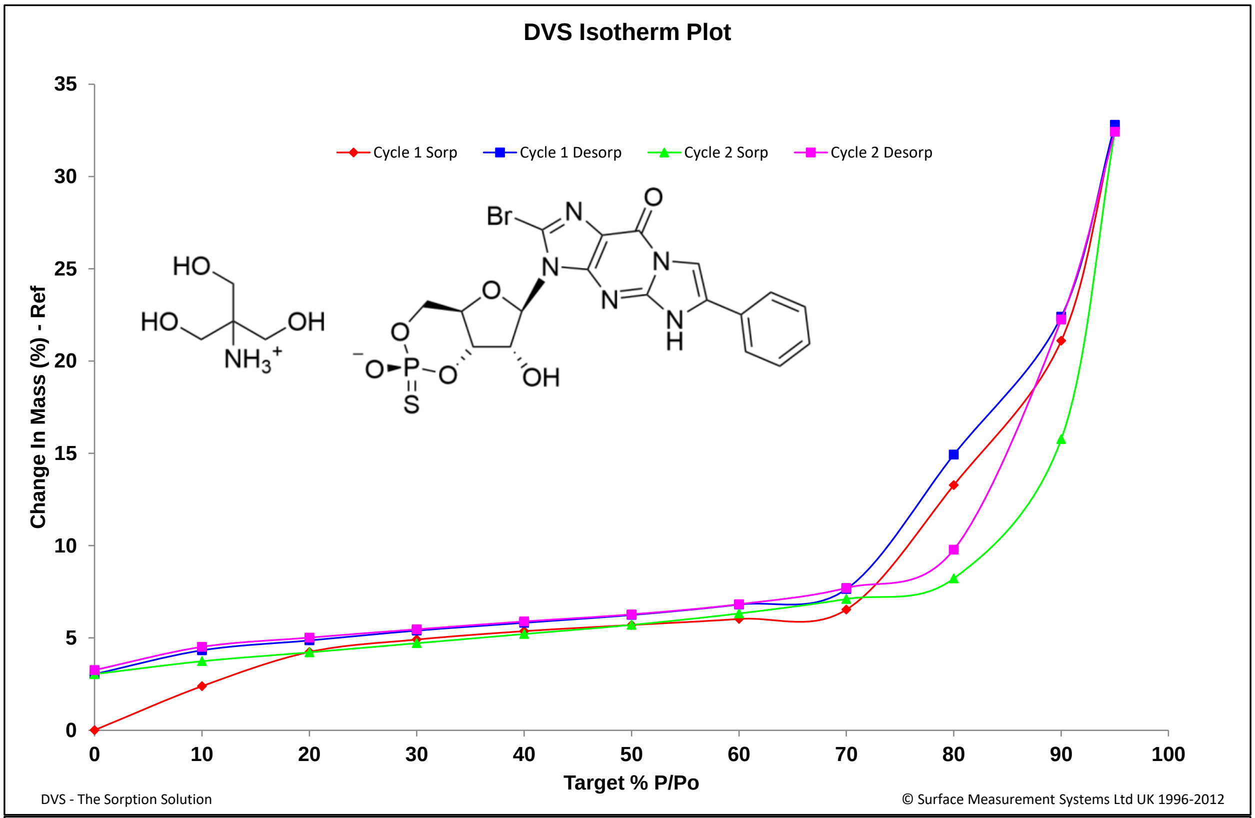
DVS Isotherm Plot
Cycle 1 Sorp
Cycle 1 Desorp
Cycle 2 Sorp
Cycle 2 Desorp
Change In Mass (%) - Ref
Target % P/Po
0
5
10
15
20
25
30
35
0
10
20
30
40
50
60
70
80
90
100
DVS - The Sorption Solution
© Surface Measurement Systems Ltd UK 1996-2012

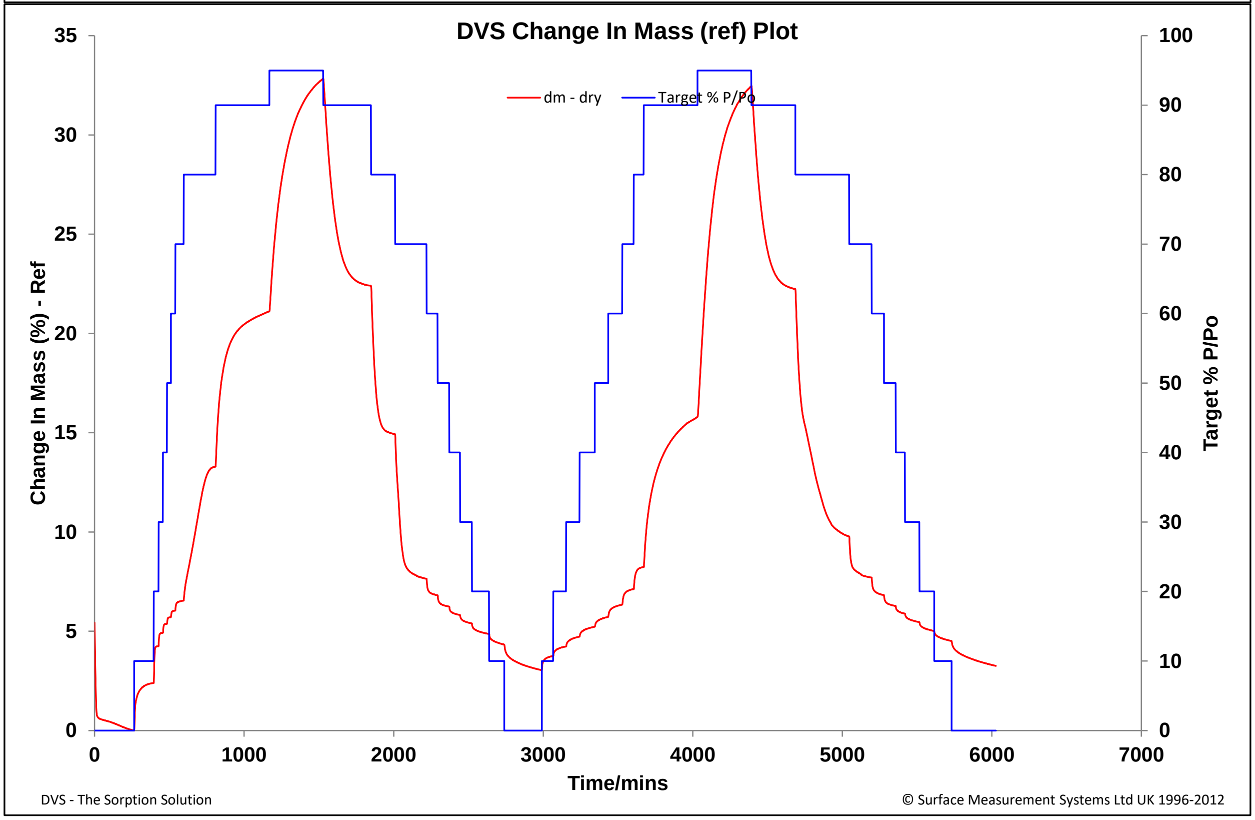
DVS Change In Mass (ref) Plot
dm - dry
Target % P/Po
Change In Mass (%) - Ref
Target % P/Po
Time/mins
0
1000
2000
3000
4000
5000
6000
7000
DVS - The Sorption Solution
© Surface Measurement Systems Ltd UK 1996-2012

XRPD (cGMPSA-NH$_4$ and cGMPSA-Tris)

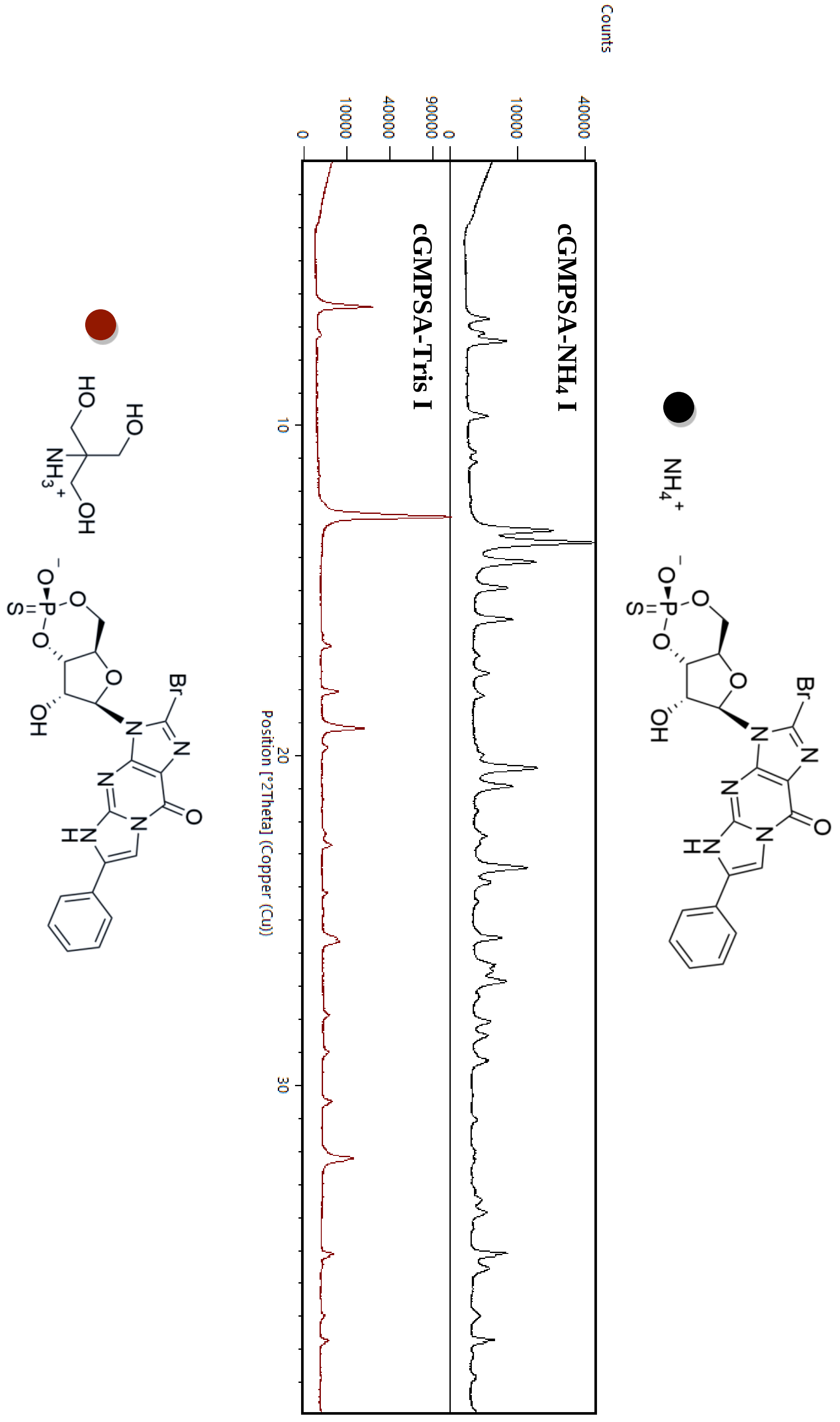

N-(Phenylmethyl)benzeneethanammonium *R*P-8-Bromo-β-phenyl-1,*N*[2]-ethenoguanosine-3′,5′-cyclicmonophosphorothioate (cGMPSA-Bnet)

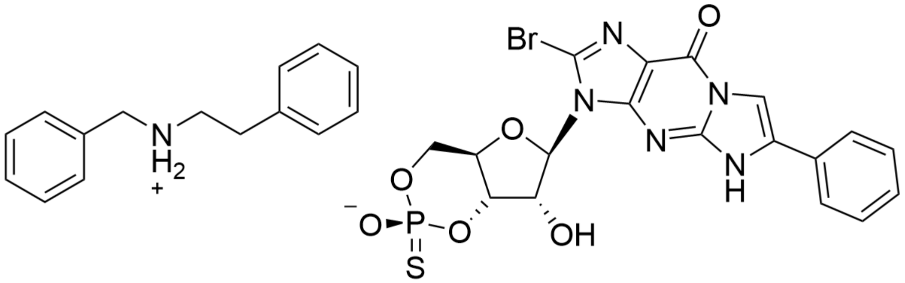

cGMPSA-TEA (12 g, 18.71 mmol) was dissolved in refluxing MeOH (600 mL, 50 volumes). Benethamine (1.1 equiv., 4.35 g, 29.58 mmol) was added to the vessel. After stirring for 1 hour, the mixture was allowed to acclimate to RT and stirred overnight. The suspension was filtered and the solid washed with MeOH (2 x 15 mL) and dried at 50 °C under a vacuum. The resulting solid was also slurried in 2-PrOH for 2 hours, after which they were filtered, washed with 2-PrOH (2 x 15 mL), and dried at 50°C under a vacuum. Yield: 86% (12.15 g, 16.17 mmol). HPLC purity: >99.9%. ^{1}H NMR (500 MHz, DMSO-d_6): δ 13.52 (br s, 1H), 9.08 (br s, 2H), 8.22 (s, 1H), 7.96–7.89 (m, 2H), 7.55–7.35 (m, 8H), 7.34–7.27 (m, 2H), 7.27–7.19 (m, 3H), 5.87 (d, *J* = 4.9 Hz, 1H), 5.75 (d, *J* = 1.6 Hz, 1H), 5.18–5.13 (m, 1H), 4.88–4.80 (m, 1H), 4.24–3.98 (m, 3H), 3.20–3.13 (m, 2H), 3.01–2.93 (m, 2H). ^{13}C NMR (126 MHz, DMScO-d_6): δ 150.4, 150.0, 145.9, 137.1, 132.0, 129.9, 129.6, 129.0, 128.8, 128.7, 128.6, 128.6, 127.8, 125.2, 122.8, 116.1, 103.6, 93.4, 75.3 (d, J_{PC} = 6.4 Hz, 1C), 71.4 (d, J_{PC} = 5.5 Hz, 1C), 69.2 (d, J_{PC} = 7.3 Hz, 1C), 65.4 (d, J_{PC} = 9.2 Hz, 1C), 50.1, 47.7, 31.5. MS (M + H^{+}) *m*/*z*: 539.97 calcd for $C_{18}H_{16}BrN_5O_6PS^{+}$; found, 540.04 (ES^{+}). DSC (exotherm, onset): 200.3 °C.

HPLC-UV-MS

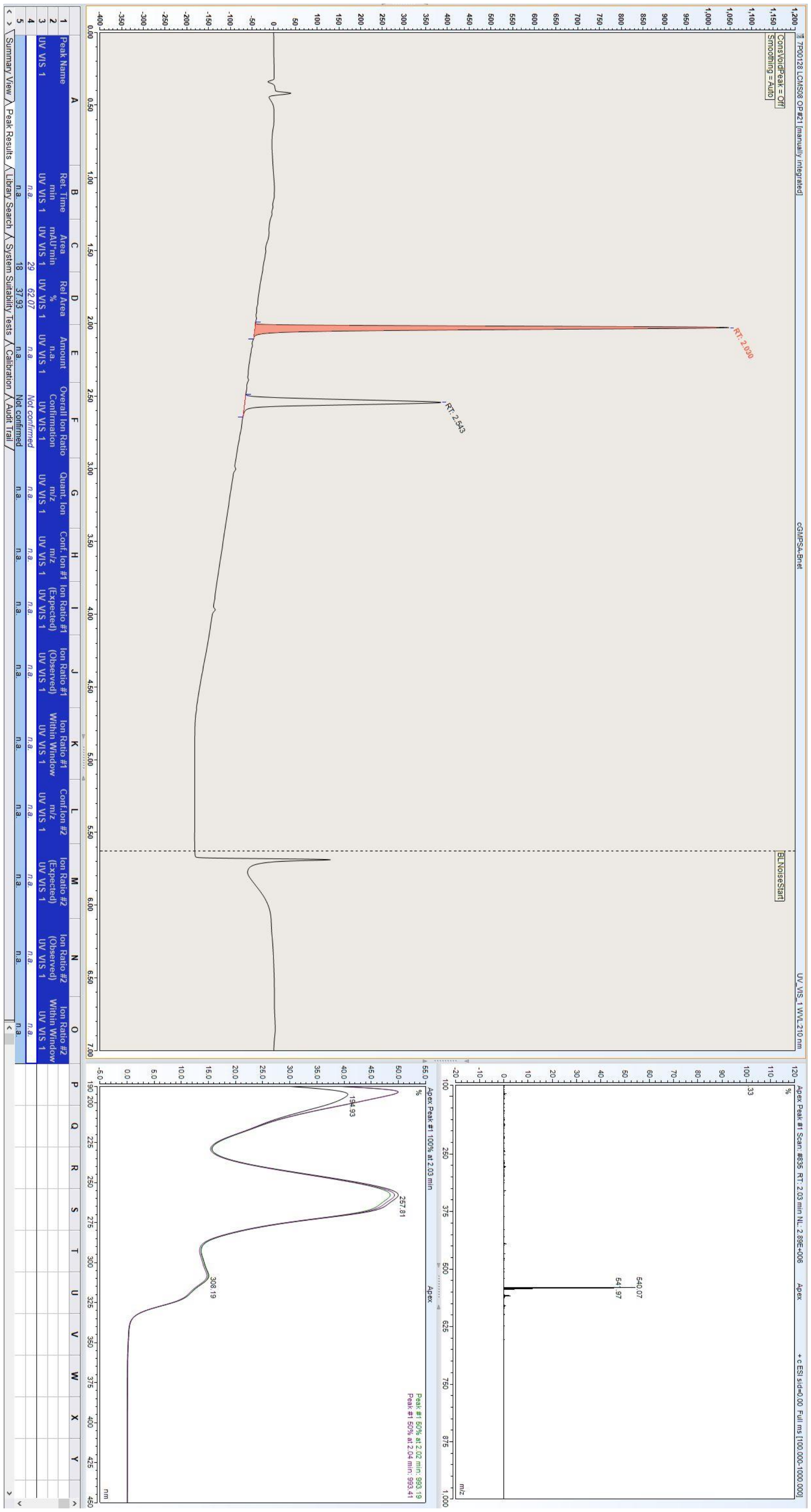

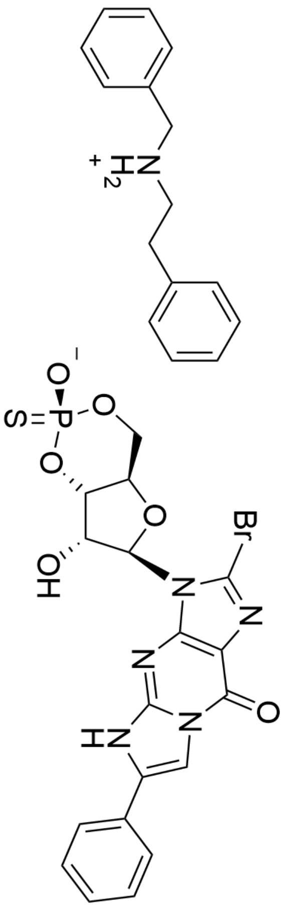

NMR

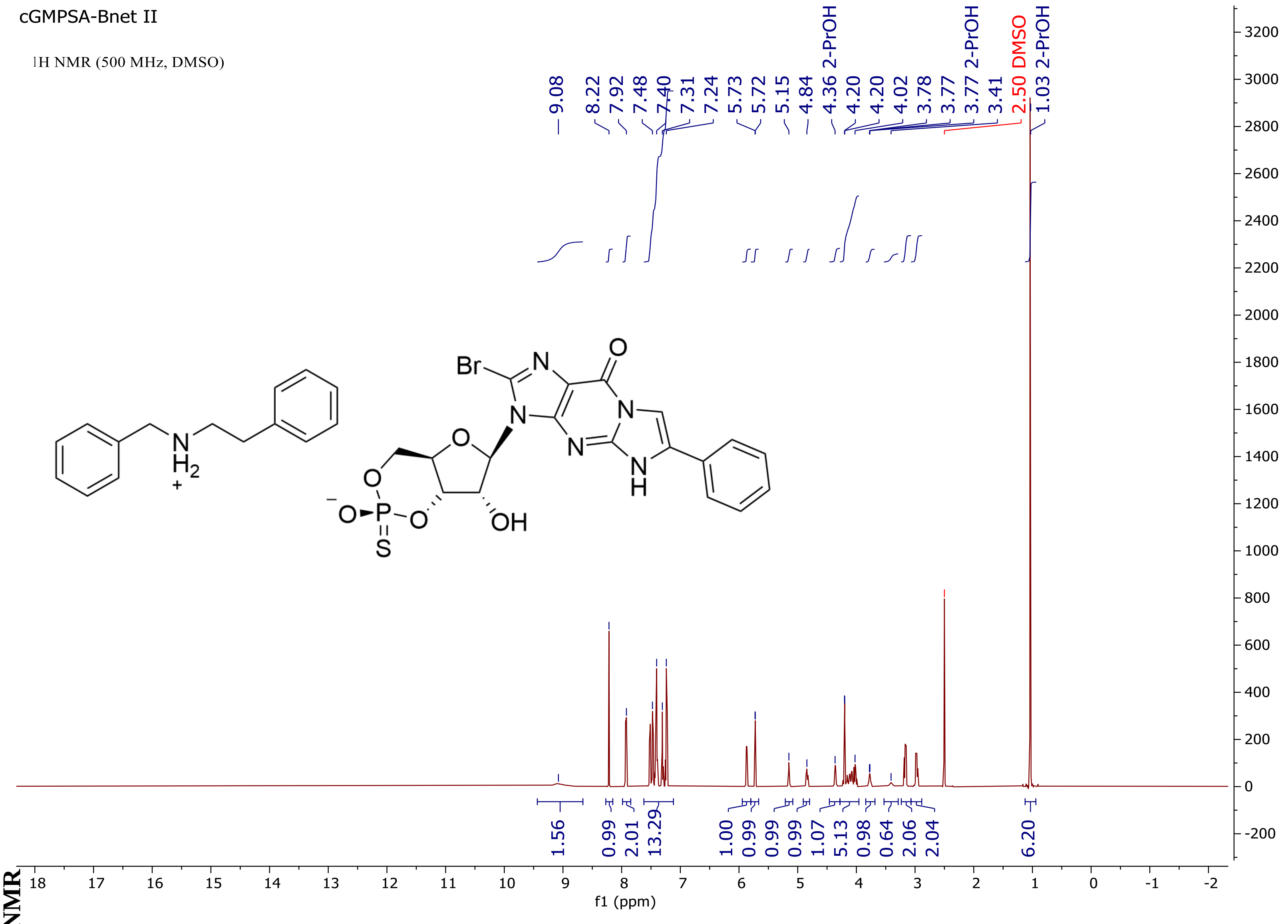
cGMPSA-Bnet II
1H NMR (500 MHz, DMSO)
9.08
8.22
7.92
7.48
7.40
7.31
7.24
5.73
5.72
5.15
4.84
4.36 2-PrOH
4.20
4.20
4.02
3.78
3.77
3.77 2-PrOH
3.41
2.50 DMSO
1.03 2-PrOH
1.56
0.99
2.01
13.29
1.00
0.99
0.99
0.99
1.07
5.13
0.98
0.64
2.06
2.04
6.20
f1 (ppm)
Br
N
O
OH
P
S
H

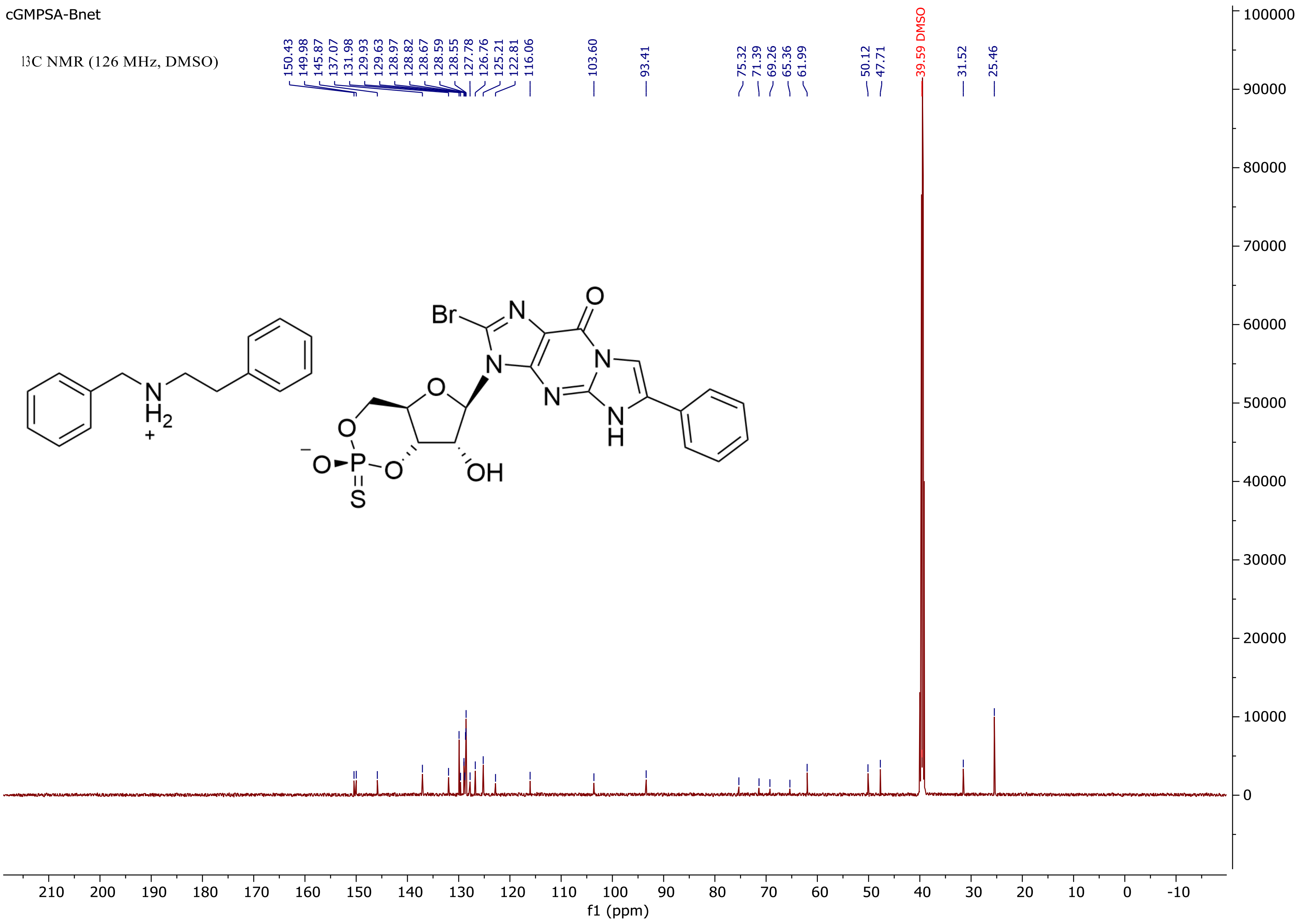
cGMPSA-Bnet
13C NMR (126 MHz, DMSO)
150.43
149.98
145.87
137.07
131.98
129.93
129.63
128.97
128.82
128.67
128.59
128.55
127.78
126.76
125.21
122.81
116.06
103.60
93.41
75.32
71.39
69.26
65.36
61.99
50.12
47.71
39.59 DMSO
31.52
25.46
f1 (ppm)

DSC & TGA

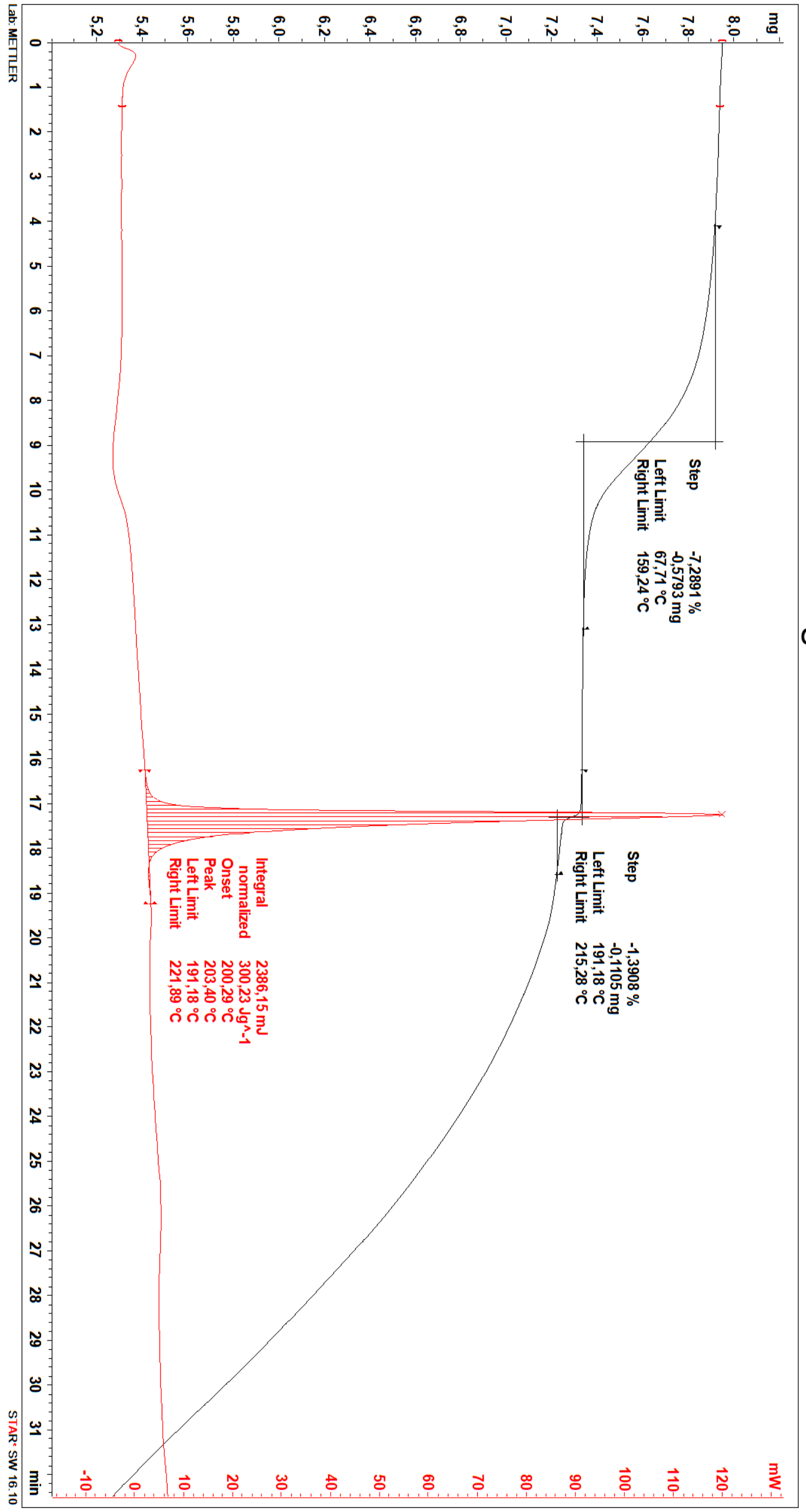

Step
-7,2891 %
-0,5793 mg
Left Limit 67,71 °C
Right Limit 159,24 °C
Step
-1,3908 %
-0,1105 mg
Left Limit 191,18 °C
Right Limit 215,28 °C
Integral 2386,15 mJ
normalized 300,23 Jg^-1
Onset 200,29 °C
Peak 203,40 °C
Left Limit 191,18 °C
Right Limit 221,89 °C
mg
mW
min
Lab: METTLER
STARe SW 16.10

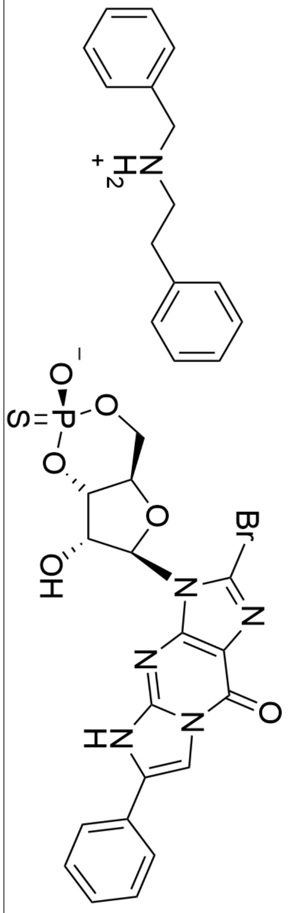

DVS

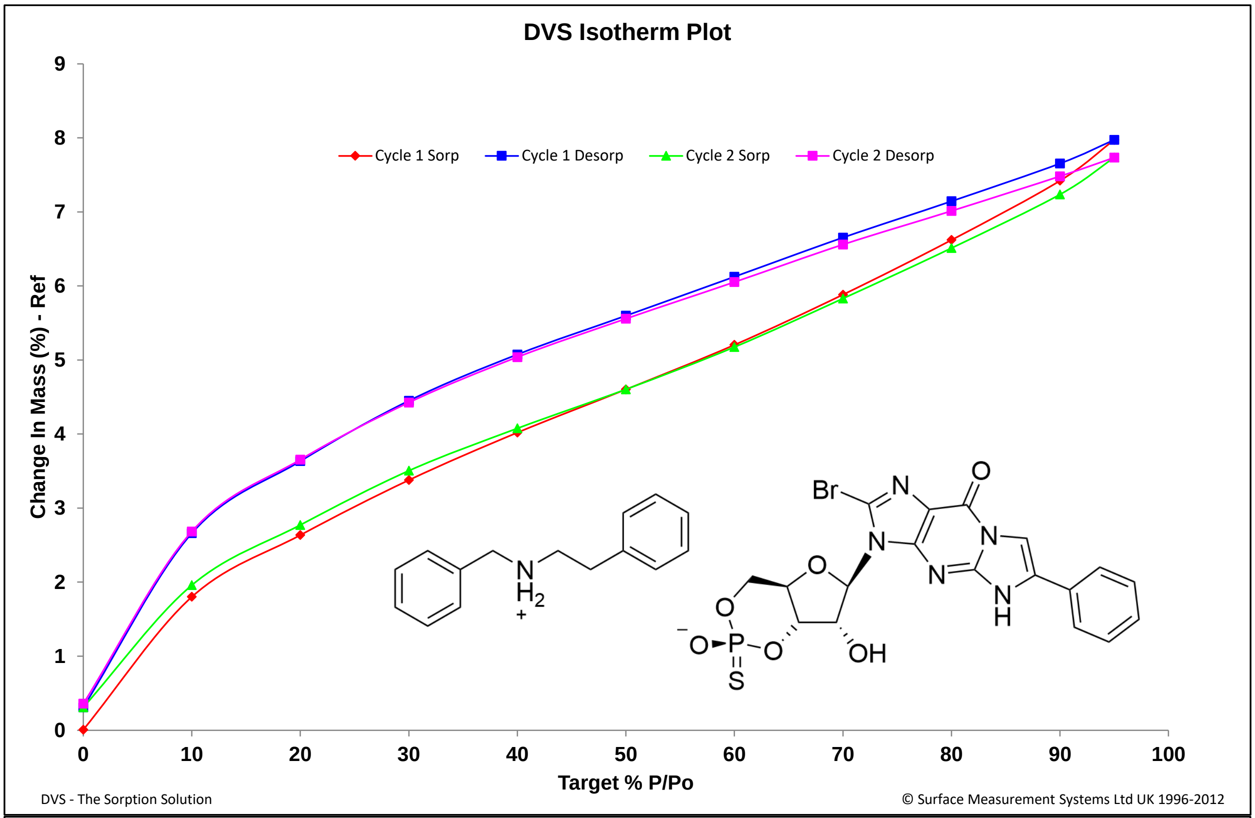

DVS Isotherm Plot
Cycle 1 Sorp
Cycle 1 Desorp
Cycle 2 Sorp
Cycle 2 Desorp
Change In Mass (%) - Ref
Target % P/Po
DVS - The Sorption Solution
© Surface Measurement Systems Ltd UK 1996-2012

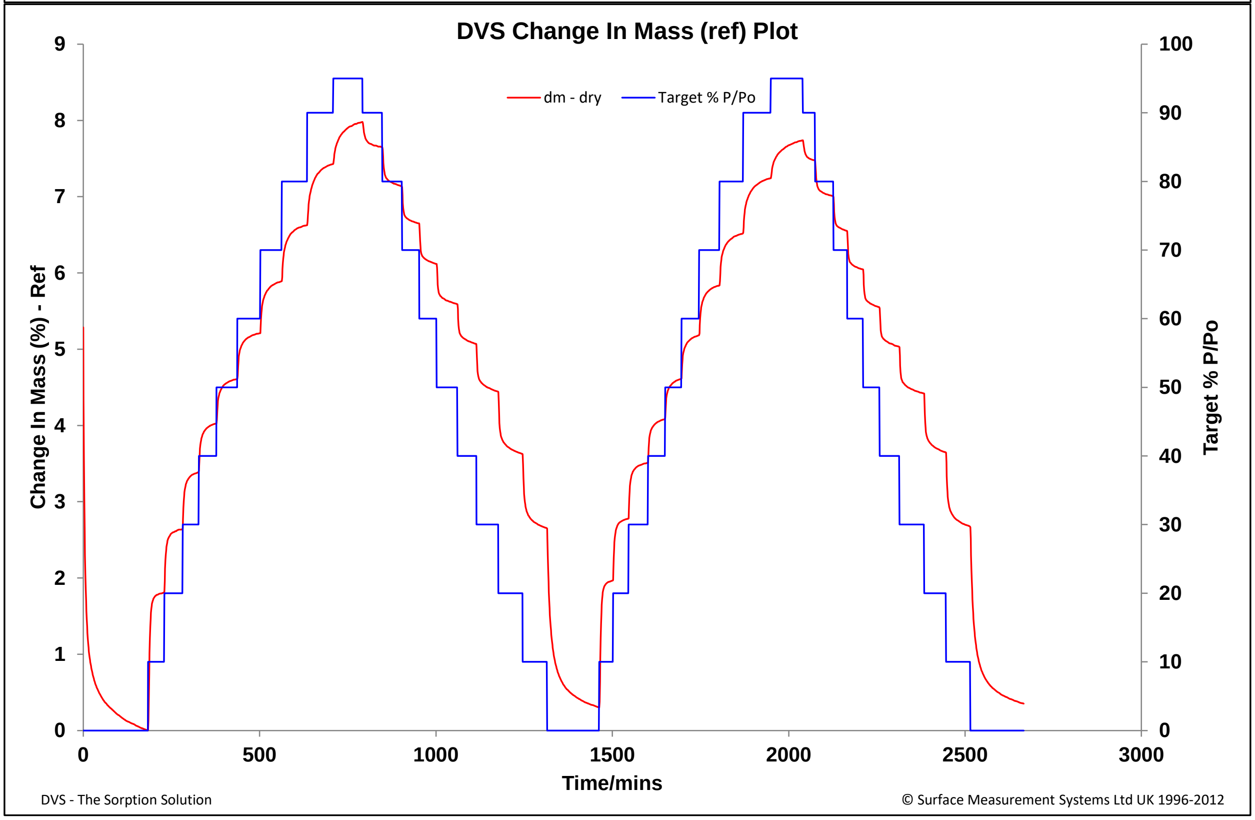

DVS Change In Mass (ref) Plot
dm - dry
Target % P/Po
Change In Mass (%) - Ref
Target % P/Po
Time/mins
DVS - The Sorption Solution
© Surface Measurement Systems Ltd UK 1996-2012

N,N′-Dibenzylethylenediammonium *R*P-8-Bromo-β-phenyl-1,*N*²-ethenoguanosine-3′,5′-cyclicmonophosphorothioate (1:2) (cGMPSA-BZ)

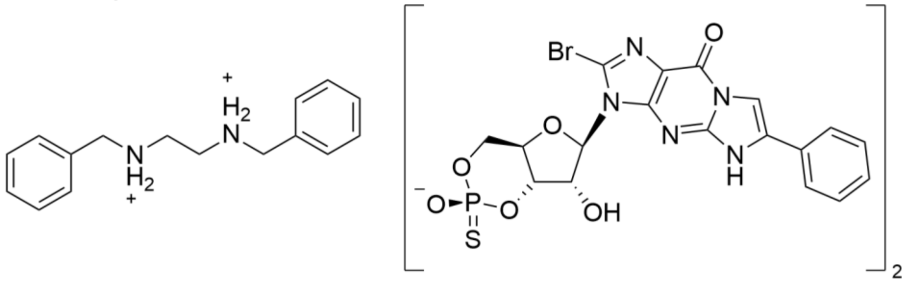

cGMPSA-TEA (14 g, 21.82 mmol) was stirred in refluxing MeOH (350 mL, 25 volumes). A methanolic solution of 0.2 M benzathine diacetate (1.1 equiv., 120 mL, 24.01 mmol) was added dropwise to the vessel. After stirring for 1 hour, the mixture was allowed to acclimate to RT and stirred overnight. The resulting suspension was filtered and the solid washed with MeOH (2 x 15 mL), followed by drying at 50°C under a vacuum. Yield: 72% (10.3 g, 7.86 mmol). HPLC purity: >98.9%. ^{1}H NMR (500 MHz, DMSO-d_6): δ 9.2 (br s, 2H), 8.2 (s, 2H), 7.95–7.87 (m, 4H), 7.55–7.43 (m, 8H), 7.43–7.35 (m, 8H), 5.75 (d, J = 1.6 Hz, 2H), 5.11 (d, J = 5.8 Hz, 2H), 4.95–4.89 (m, 2H), 4.91–4.84 (m, 1H), 4.28–4.00 (m, 6H), 4.23 (d, J = 5.9 Hz, 4H), 3.48 (br s, 4H). ^{13}C NMR (126 MHz, DMSO-d_6): δ 150.4, 150.0, 145.9, 129.8, 129.6, 129.0, 129.0, 128.8, 128.7, 127.7, 125.2, 122.7, 116.0, 103.6, 93.4, 75.4 (d, J_{PC} = 6.4 Hz, 2C), 71.4 (d, J_{PC} = 5.5 Hz, 2C), 69.4 (d, J_{PC} = 7.3 Hz, 2C), 65.6 (d, J_{PC} = 8.3 Hz, 2C), 50.5, 42.7. MS (M + H$^+$) m/z: 539.97 calcd for $C_{18}H_{16}BrN_5O_6PS^+$; found, 539.92 (ES$^+$). DSC (exotherm, onset): 206.5 °C.

HPLC-UV-MS

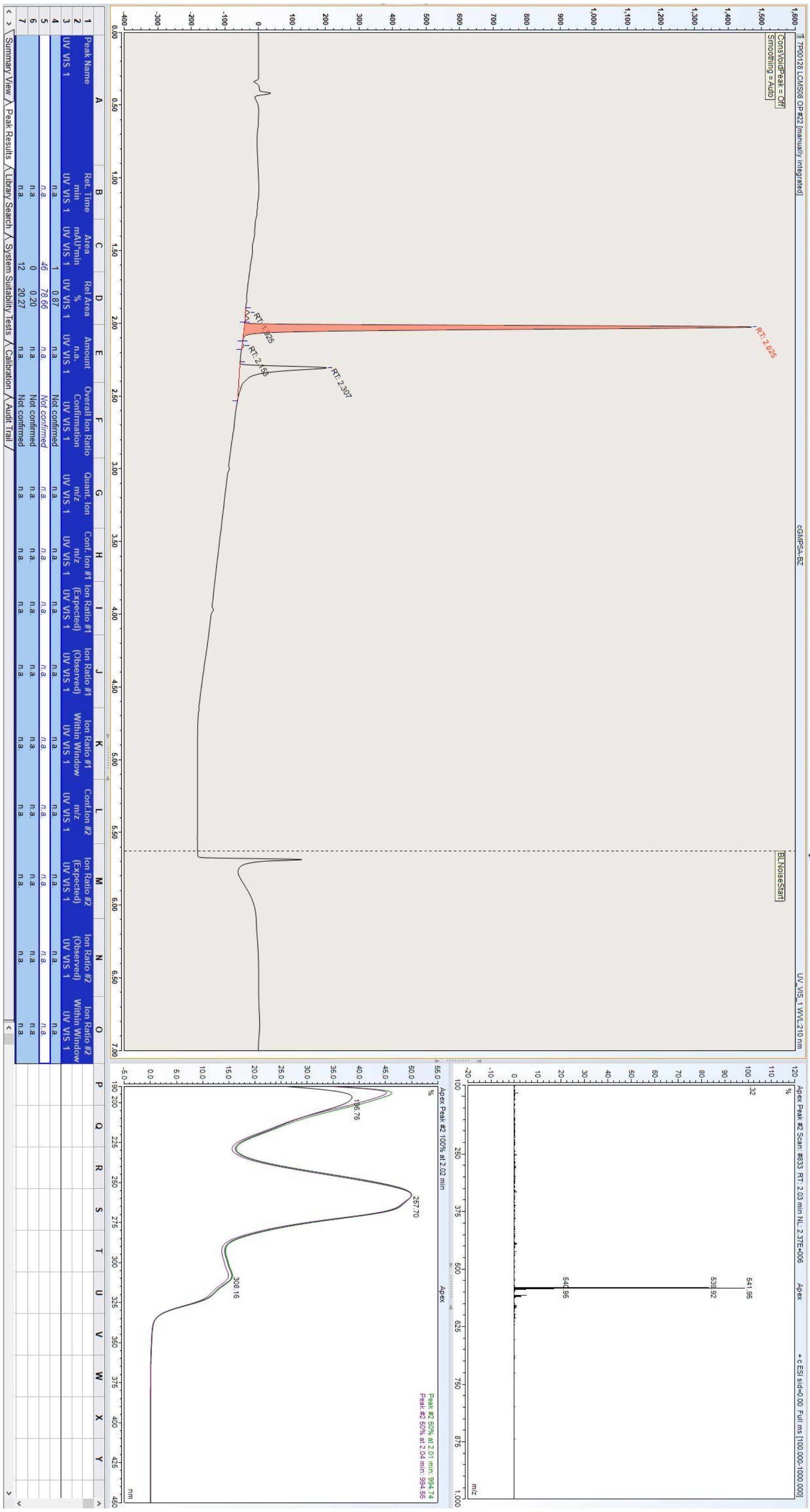

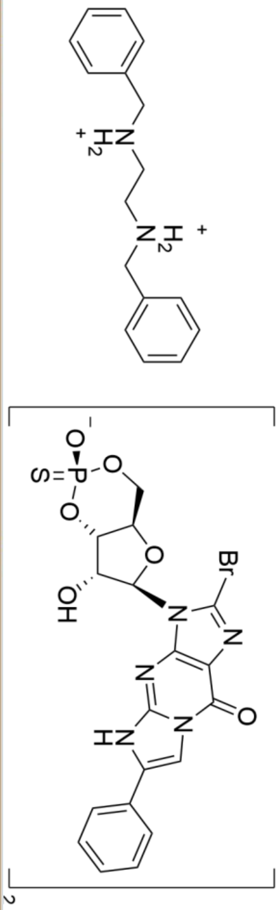

NMR

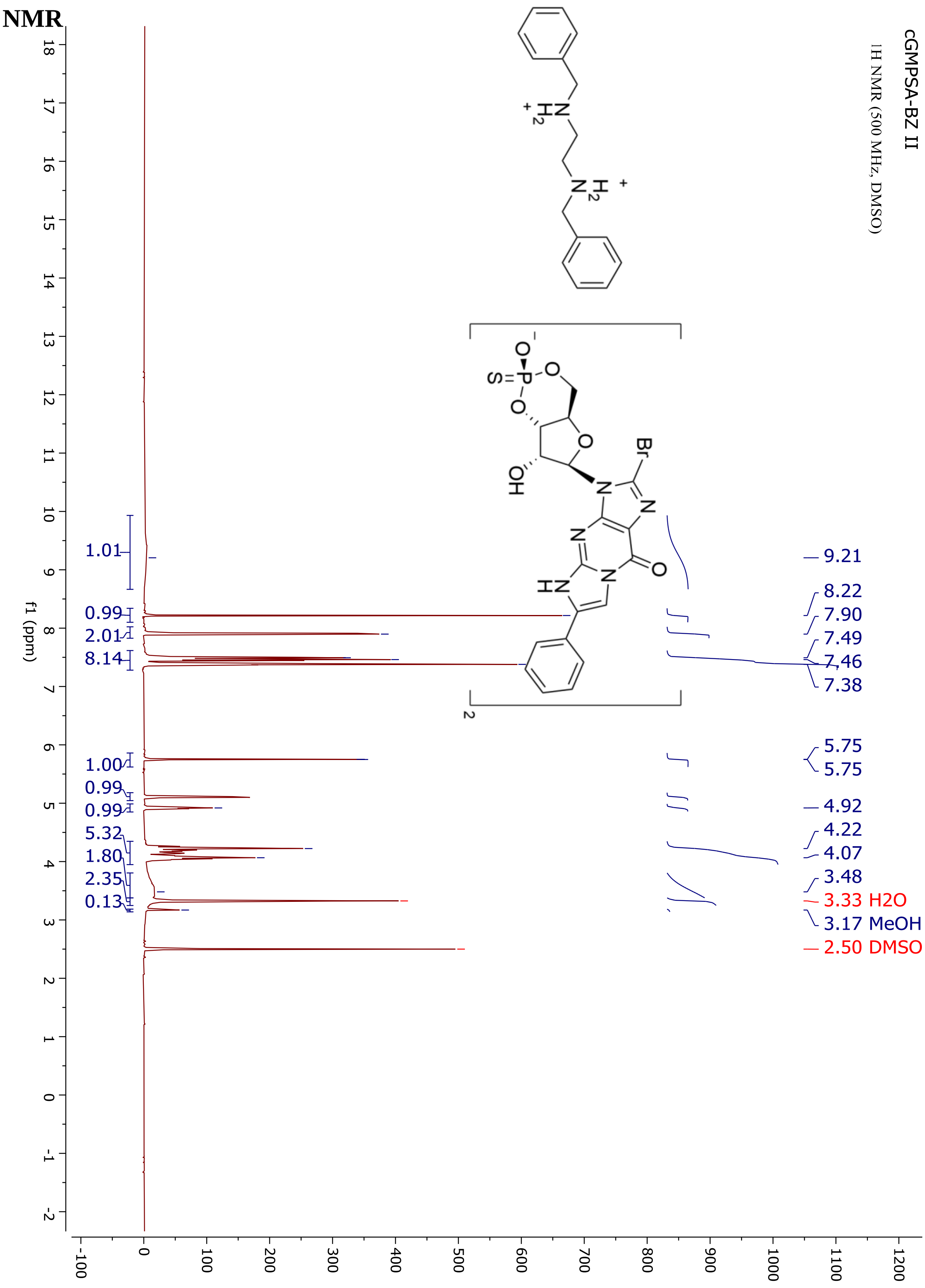
cGMPSA-BZ II
1H NMR (500 MHz, DMSO)
9.21
8.22
7.90
7.49
7.46
7.38
5.75
5.75
4.92
4.22
4.07
3.48
3.33 H2O
3.17 MeOH
2.50 DMSO
1.01
0.99
2.01
8.14
1.00
0.99
0.99
5.32
1.80
2.35
0.13
f1 (ppm)
Br
OH
NH
O
S
P
H2
2

cGMPSA-BZ

13C NMR (126 MHz, DMSO)

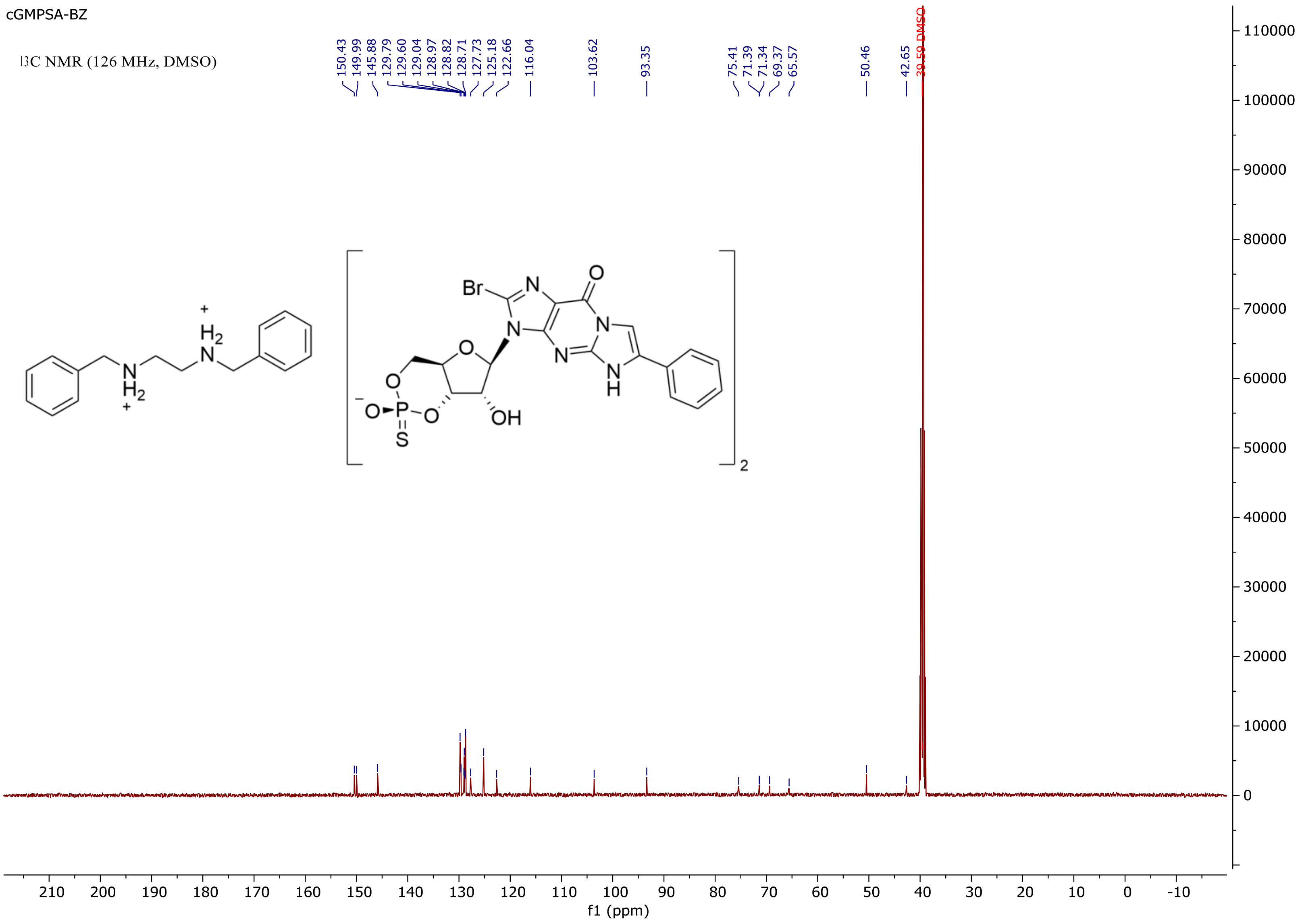

DSC & TGA

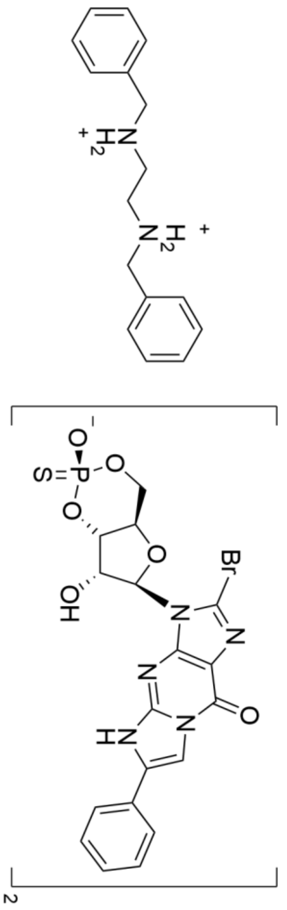

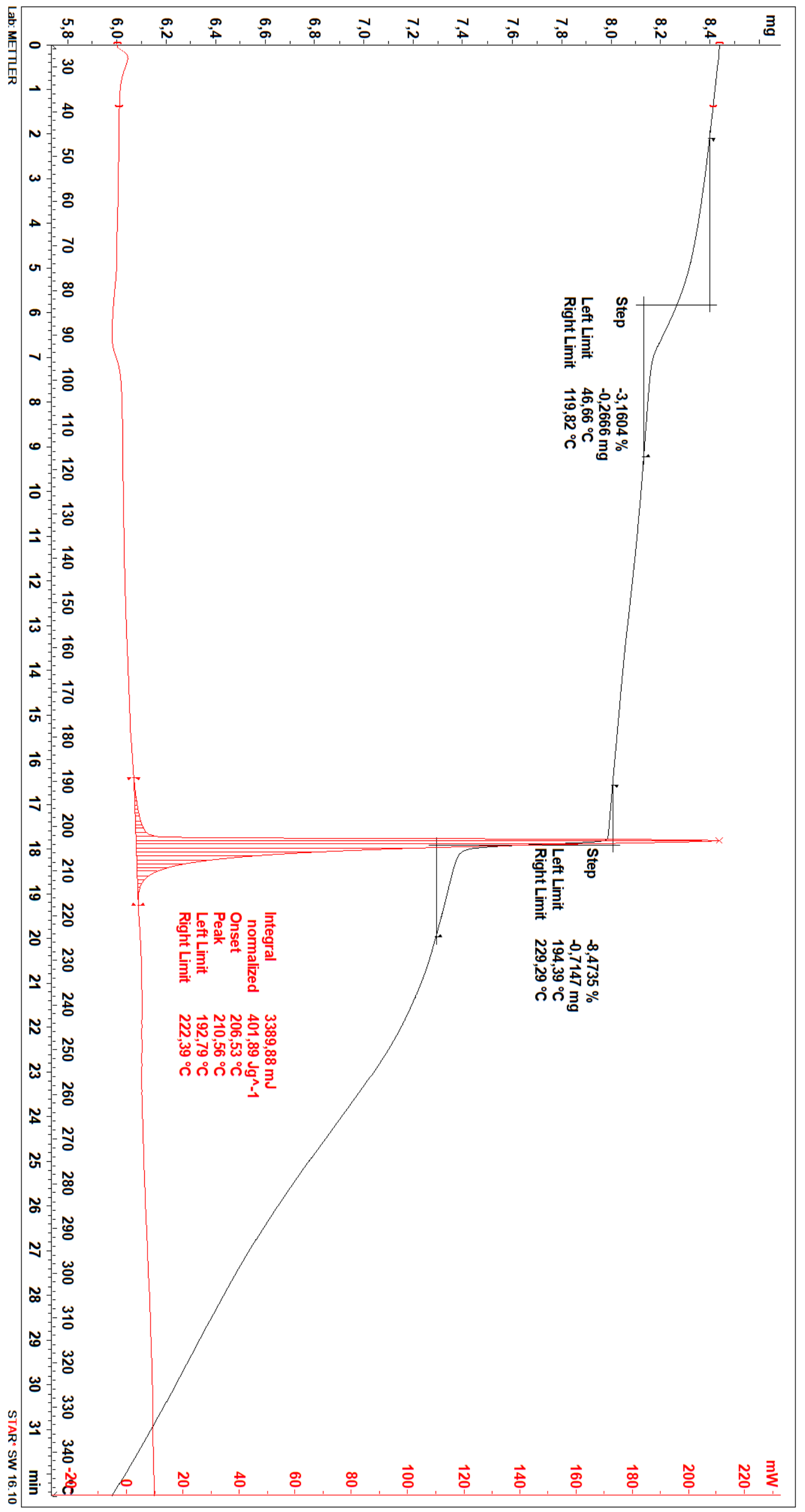

Step -3,1604 %
-0,2666 mg
Left Limit 46,66 °C
Right Limit 119,82 °C
Step -8,4735 %
-0,7147 mg
Left Limit 194,39 °C
Right Limit 229,29 °C
Integral 3389,88 mJ
normalized 401,89 Jg^-1
Onset 206,53 °C
Peak 210,56 °C
Left Limit 192,79 °C
Right Limit 222,39 °C
Lab: METTLER
STARe SW 16.10

DVS

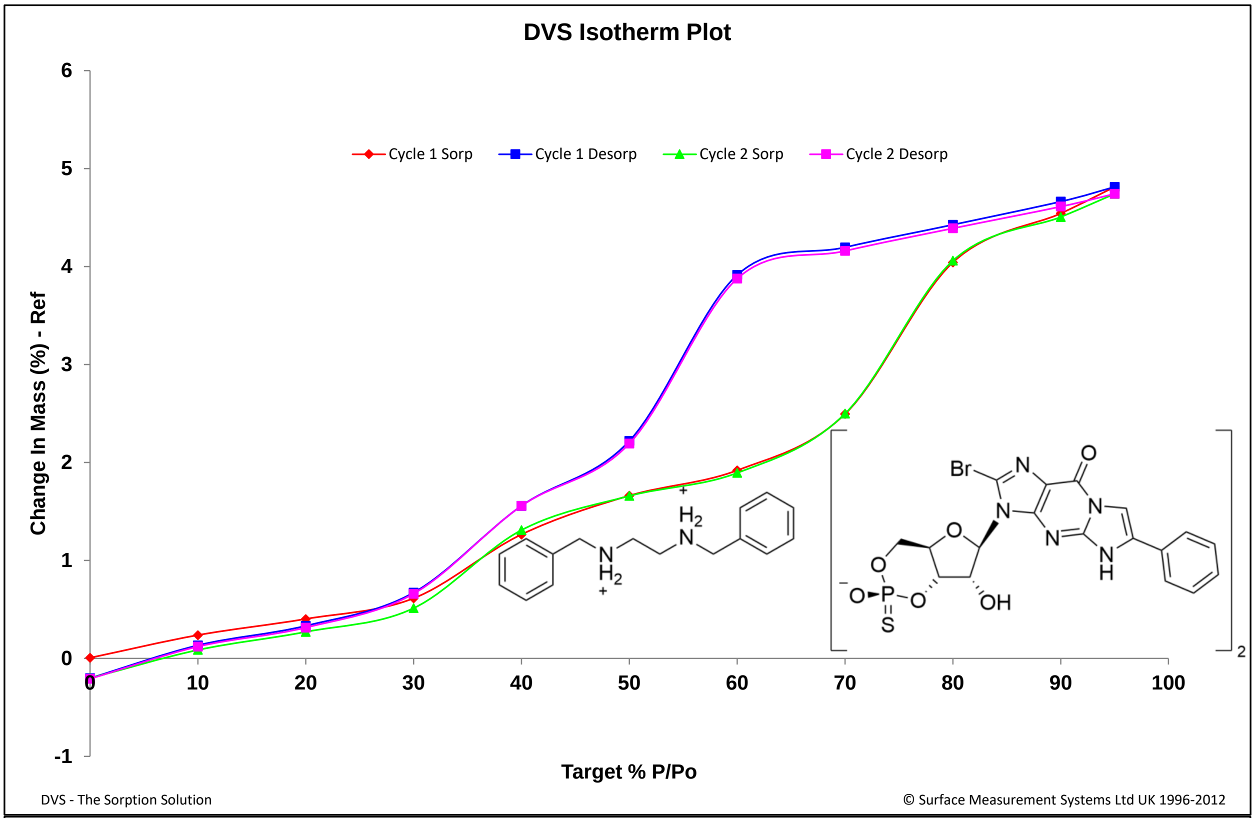
DVS Isotherm Plot
Cycle 1 Sorp
Cycle 1 Desorp
Cycle 2 Sorp
Cycle 2 Desorp
Change In Mass (%) - Ref
Target % P/Po
DVS - The Sorption Solution
© Surface Measurement Systems Ltd UK 1996-2012

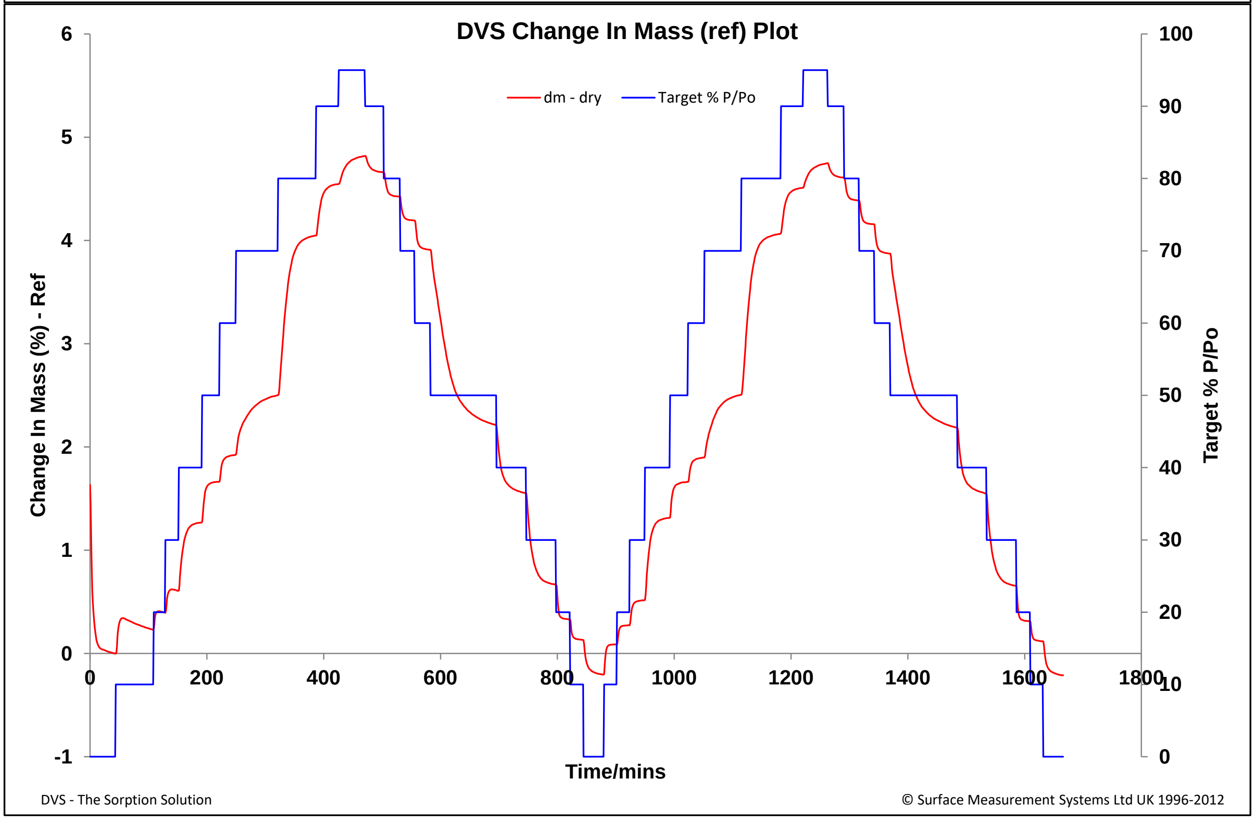
DVS Change In Mass (ref) Plot
dm - dry
Target % P/Po
Change In Mass (%) - Ref
Target % P/Po
Time/mins
DVS - The Sorption Solution
© Surface Measurement Systems Ltd UK 1996-2012

XRPD (cGMPSA-Bnet and cGMPSA-BZ)

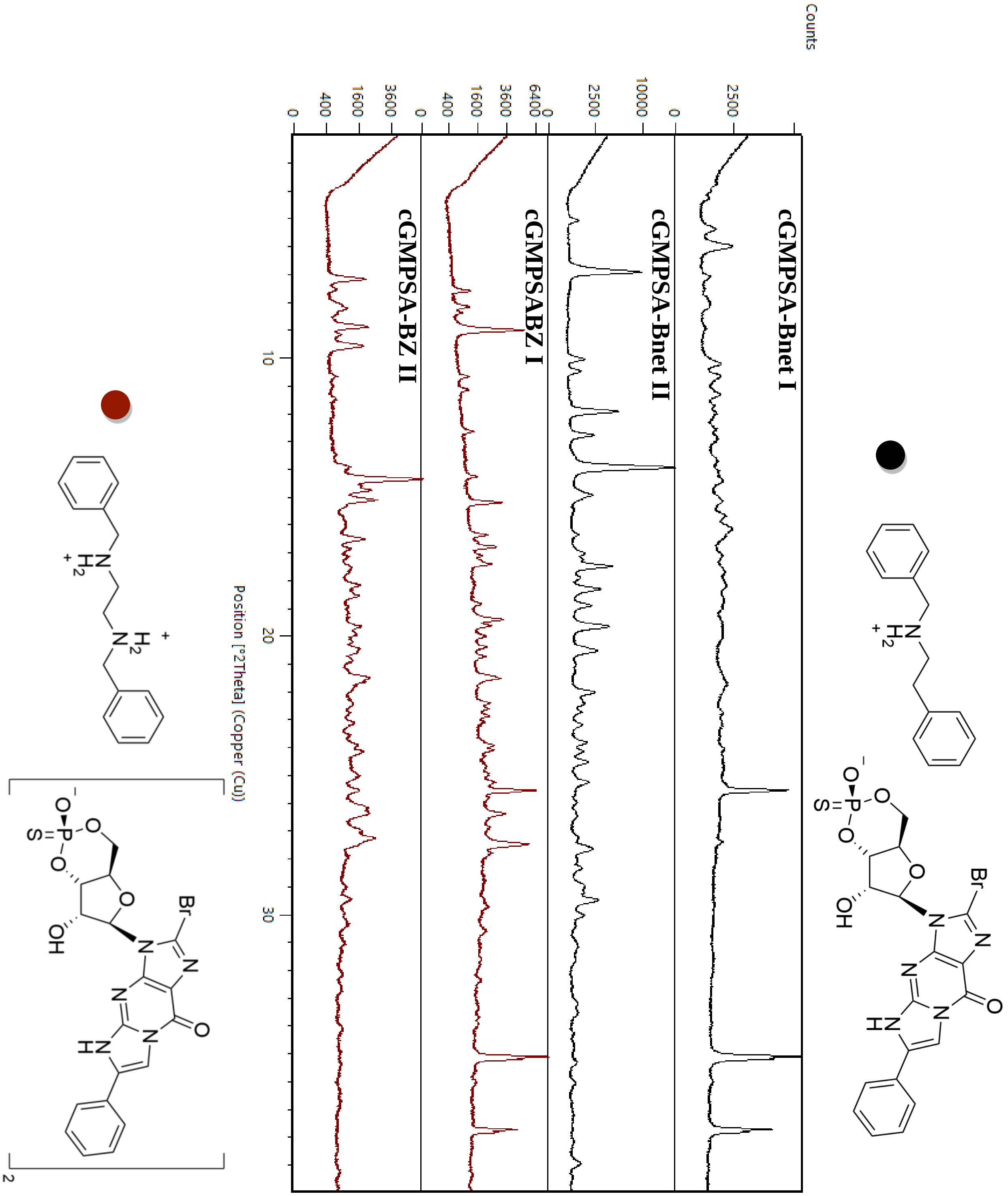

Aqueous solubility measurements

Compound	mg / mL	Standard Deviation	pH of mixture	Standard Deviation
**cGMPSA-TEA**	8.9	1.7	4,38	0.4
**cGMPSA-H**	1.7	0.1	2,47	0.2
**cGMPSA-Na**	38.4	11.1	5,45	0.2
**cGMPSA-Ca**	0.28	0.2	6,88	0.5
**cGMPSA-NH_4**	7.9	8.0	3,16	0.1
**cGMPSA-Tris**	13.5	3.5	8,45	0.1
**cGMPSA-Bnet**	0.05	0.03	7,17	0.04
**cGMPSA-BZ**	0.01	0.01	6,81	0.1

pH-Dependent aqueous solubility measurements.

Compound	0.1M $HCl_{(aq)}$		0.1M $NaOH_{(aq)}$	
	µg / mL (SD)	pH of mixture (SD)	mg / mL (SD)	pH of mixture (SD)
**cGMPSA-TEA**	5.0 (4) [a]	1.1 (0.055) [a]	51.6 (4.671)	10.1 (0.033)
**cGMPSA-H**	0.1 (0.1) [a]	1.0 (0.025) [a]	25.0 (8.130)[c]	13.40 (0.031)[c]
**cGMPSA-Na**	3.0 (1) [a]	1.2 (0.160) [a]	66.4 (2.003)	10.4 (0.120)
**cGMPSA-Ca**	25.0 (17)	1.0 (0.034)	8.4 (1.910)	12.5 (0.066)
**cGMPSA-NH_4**	8.0 (2) [a]	1.1 (0.050) [a]	36.8 (14.041)	9.6 (0.041)
**cGMPSA-Tris**	21.0 (15)	1.1 (0.058)	55.0 (3.959)	9.2 (0.082)
**cGMPSA-Bnet**	4.82 (n.a.) [b]	1.1 (n.a.) [b]	11.6 (2.693)	12.6 (0.066)
**cGMPSA-BZ**	0.22 (n.a.) [b]	1.0 (n.a.) [b]	31.1 (0.812)	10.8 (0.313)

Standard Deviation (SD) shown in parentheses. [a]Only two data points available; [b]Only one data point available. [C]1M NaOH used.

Single Crystal X-ray Diffraction

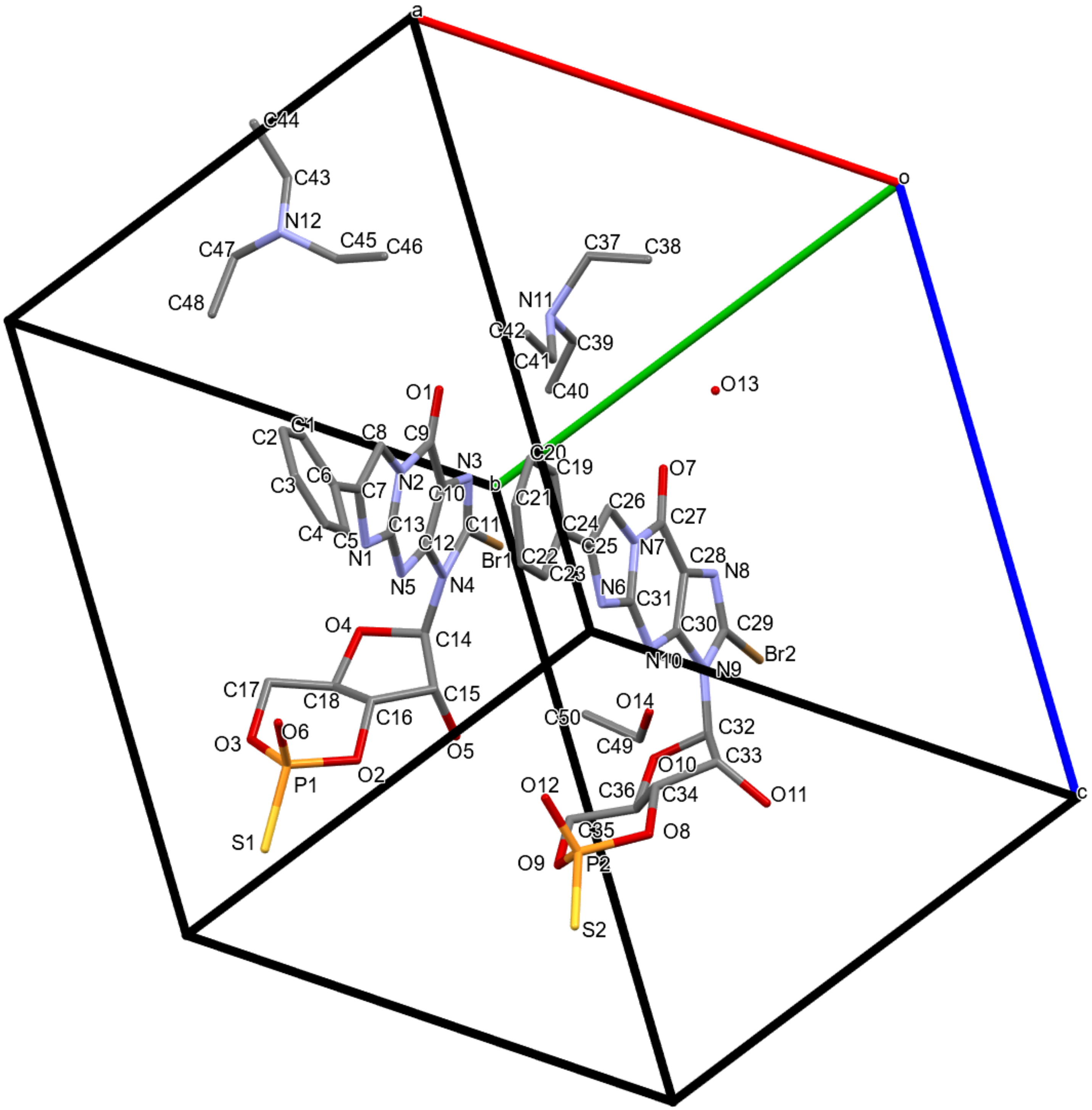

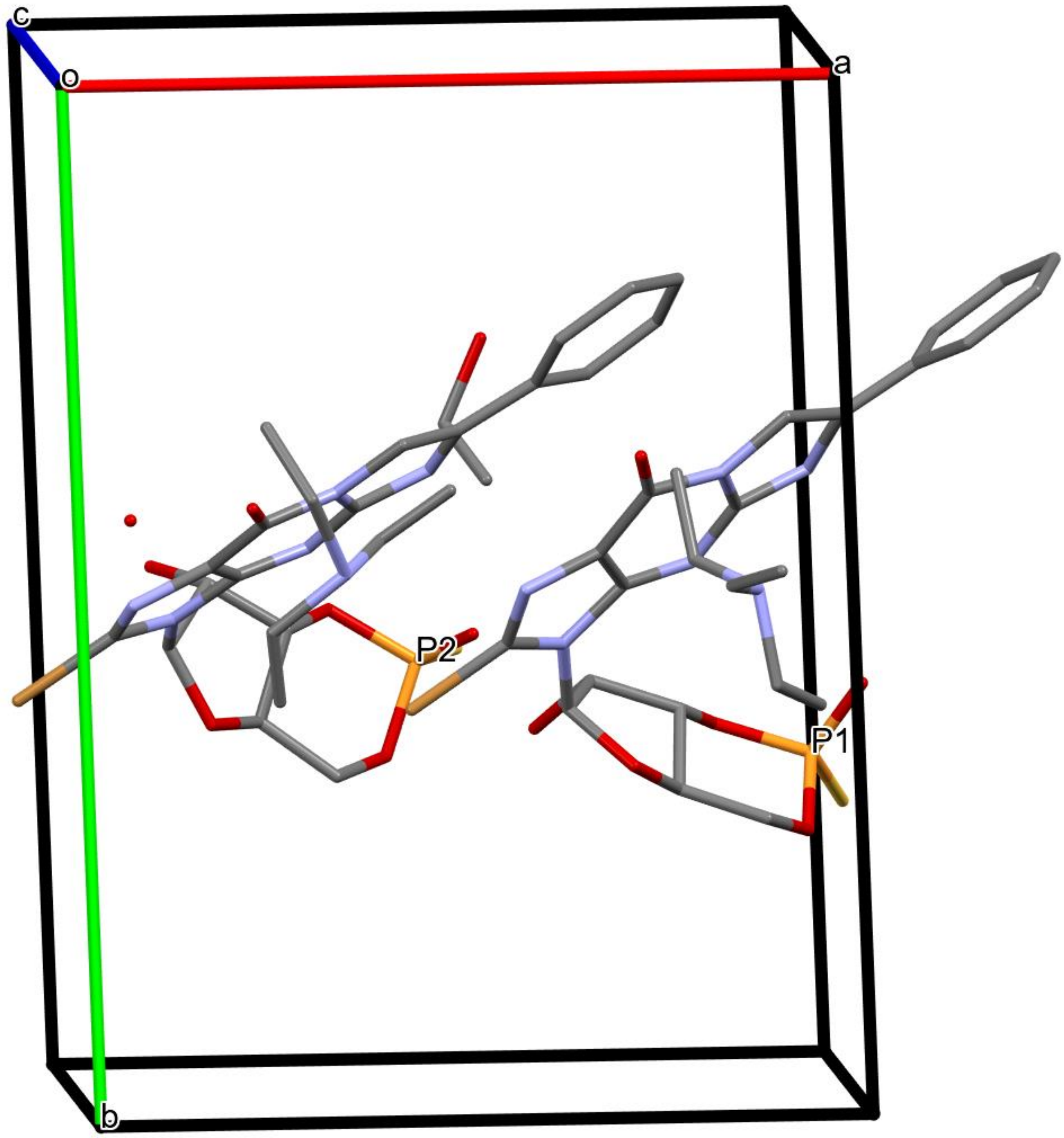
c
o
a
b
P2
P1

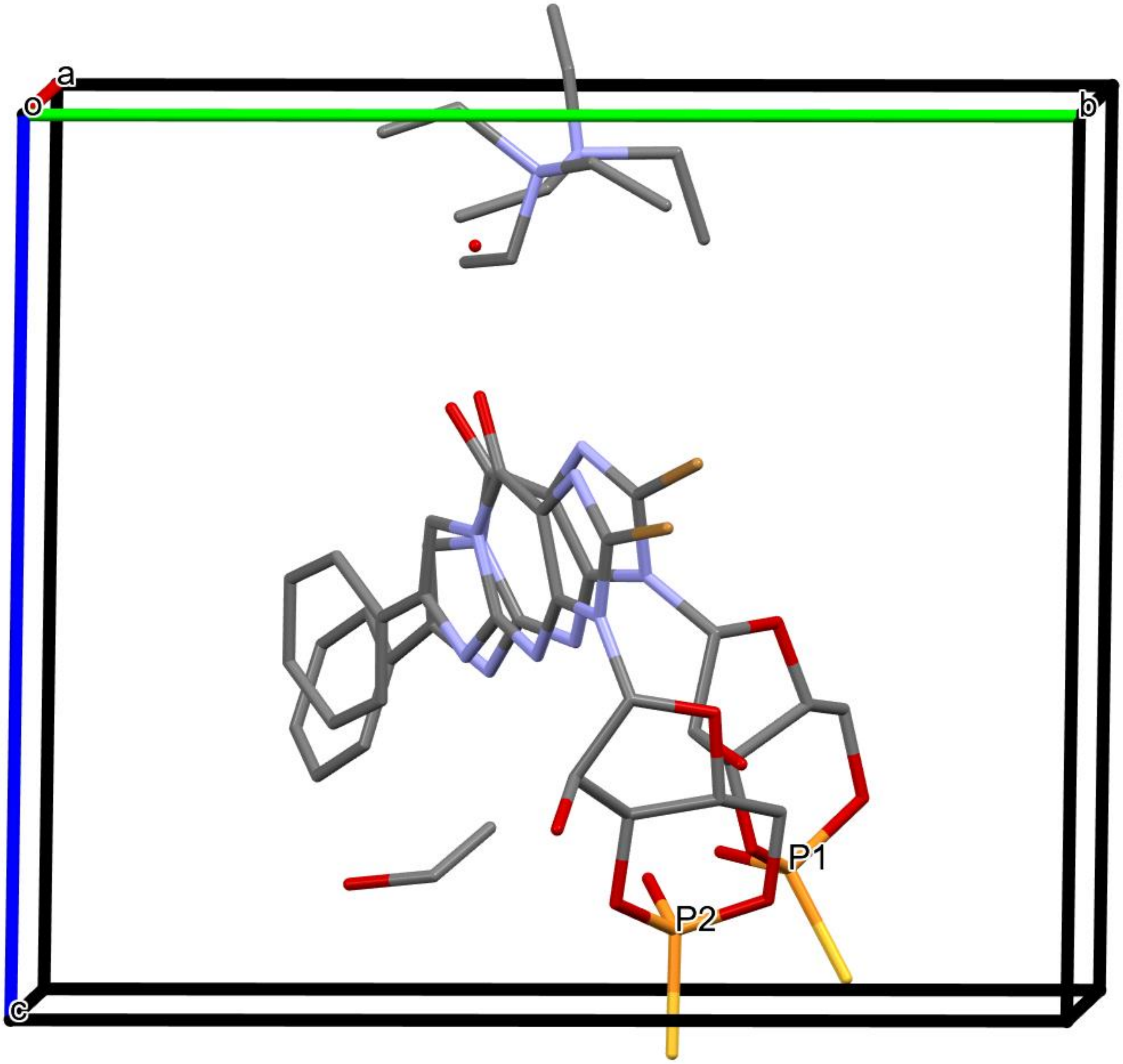
a
o
b
c
P1
P2

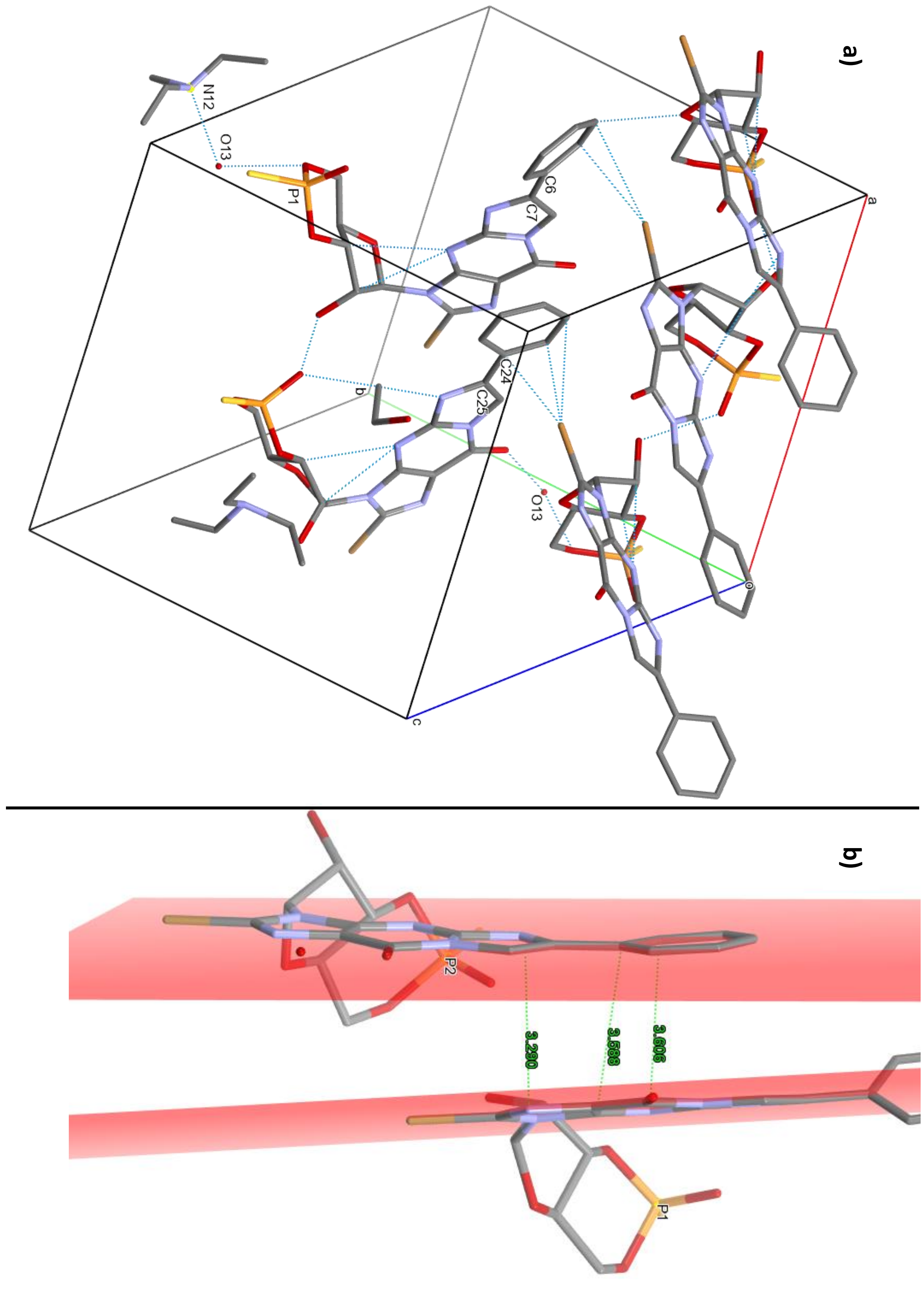
a)
b)
N12
O13
P1
C6
C7
C24
C25
P2
3.290
3.588
3.606

References

[1] O. Pérez, N. Schipper, M. Bollmark, *Org. Process Res. Dev.* **2021**, *25*, 2453-2460.
